# Supplementary material for: Model Optimization and In Silico Analysis of Potential Dipeptidyl Peptidase IV Antagonists from GC-MS Identified Compounds in Nauclea latifolia Leaf Extracts
Source: Int J Mol Sci. 2019 Nov 25;20(23):5913. doi: 10.3390/ijms20235913 (PMC6929178; doi:10.3390/ijms20235913)

Peak#	R.Time	I.Time	F.Time	Area	Area%	Height	Height%	A/H	Mark	Name
26	16.208	15.817	16.250	73694026	8.47	4672086	2.38	15.77	V	1,2,3,5-Cyclohexanetetrol, (1.alpha.,2.beta.
27	16.278	16.250	16.417	31667436	3.64	5612423	2.86	5.64	V	1,2,3,5-Cyclohexanetetrol, (1.alpha.,2.beta.
28	16.592	16.508	16.642	12050977	1.38	2138344	1.09	5.64	V	Tridecanoic acid
29	16.770	16.642	16.800	18151447	2.09	3289426	1.68	5.52	V	Dodecanoic acid
30	16.908	16.800	16.942	19330254	2.22	3572980	1.82	5.41	V	[1,1'-Bicyclopropyl]-2-octanoic acid, 2'-hex
31	16.984	16.942	17.042	21504642	2.47	5986904	3.06	3.59	V	2-O-p-Methylphenyl-1-thio-.beta.-d-glucos
32	17.099	17.042	17.183	26710369	3.07	4339717	2.21	6.15	V	[1,1'-Bicyclopropyl]-2-octanoic acid, 2'-hex
33	17.333	17.308	17.442	23316124	2.68	3506977	1.79	6.65	V	3-O-Methyl-d-glucose
34	17.473	17.442	17.650	8891593	1.02	2056313	1.05	4.32	V	[1,1'-Bicyclopropyl]-2-octanoic acid, 2'-hex
35	18.126	17.942	18.158	94546881	10.86	18962907	9.68	4.99	V	n-Hexadecanoic acid
36	18.195	18.158	18.250	63743335	7.32	25631265	13.08	2.49	V	Hexadecanoic acid, ethyl ester
37	18.291	18.250	18.350	16226477	1.86	3504361	1.79	4.63	V	.gamma.-Sitosterol
38	18.375	18.350	18.433	10312008	1.18	2771191	1.41	3.72	V	.gamma.-Sitosterol
39	18.814	18.750	18.917	9045664	1.04	2313469	1.18	3.91	V	9,9-Dimethoxybicyclo[3.3.1]nona-2,4-dion
40	18.983	18.917	19.117	63043592	7.24	29892676	15.25	2.11	V	Phytol
41	19.319	19.117	19.408	156657817	18.00	26630343	13.59	5.88	V	Ethyl Oleate
42	19.441	19.408	19.700	47999252	5.51	14393576	7.35	3.33	V	Octadecanoic acid, ethyl ester
43	19.824	19.700	19.950	26562080	3.05	2736147	1.40	9.71	V	Androstan-17-one, 16,16-dimethyl-, (5.alph
44	20.088	19.950	20.208	23442194	2.69	2783062	1.42	8.42	V	1-Naphthalenol, decahydro-1,4a-dimethyl-7
45	20.390	20.208	20.583	22336031	2.57	2271101	1.16	9.83	V	Androstan-17-one, 16,16-dimethyl-, (5.alph
46	20.652	20.583	20.675	5393308	0.62	2973493	1.52	1.81	V	Hexadecanoic acid, ethyl ester
47	20.721	20.675	20.767	2487778	0.29	900702	0.46	2.76	V	17-Octadecynoic acid
				870348710	100.00	195958256	100.00			

Library

<< Target >>
Line#:1 R.Time:7.700(Scan#:265) MassPeaks:56
RawMode:Single 7.700(265) BasePeak:55.00(283402)
BG Mode:None Group 1 - Event 1

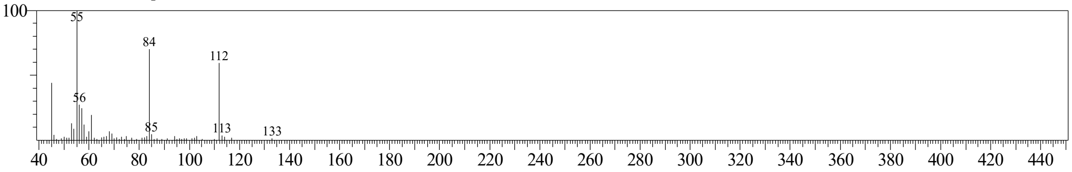

Hit#:1 Entry:2882 Library:NIST11s.lib
SI:79 Formula:C6H8O2 CAS:504-02-9 MolWeight:112 RetIndex:1062
CompName:1,3-Cyclohexanedione $$ Dihydroresorcinol $$ Cyclohexane-1,3-dione $$ Hydroresorcinol $$ Resorcinol, dihydro- $$ 1,3-Benzenediol, dihydro- $

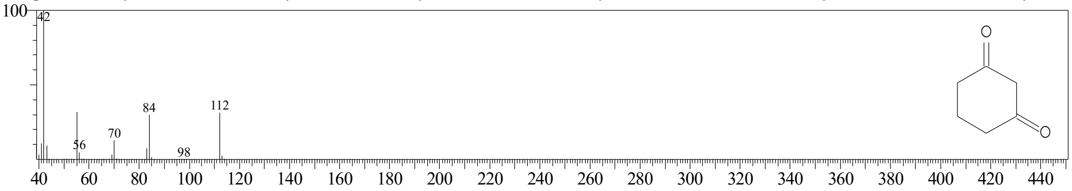

Hit#:2 Entry:2923 Library:NIST11s.lib
SI:78 Formula:C6H12N2 CAS:280-57-9 MolWeight:112 RetIndex:965
CompName:Triethylenediamine $$ 1,4-Diazabicyclo[2.2.2]octane $$ Bicyclo[2.2.2]-1,4-diazaoctane $$ D 33LV $$ Dabco $$ Dabco 33LV $$ N,N'-endo-Ethyler

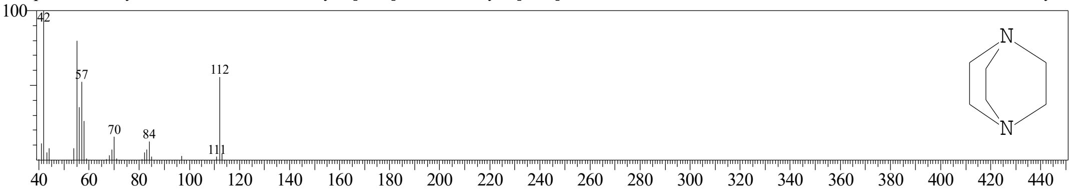

Hit#:3 Entry:2053 Library:NIST11s.lib
SI:78 Formula:C5H10O2 CAS:5057-99-8 MolWeight:102 RetIndex:976
CompName:1,2-Cyclopentanediol, trans- $$ trans-1,2-Cyclopentanediol $$ 1,2-Cyclopentanediol # $$

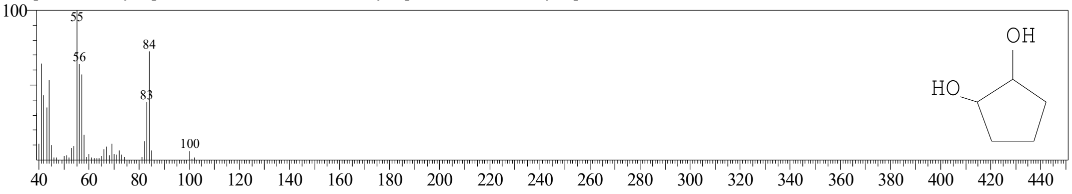

<< Target >>
Line#:1 R.Time:7.700(Scan#:265) MassPeaks:56
RawMode:Single 7.700(265) BasePeak:55.00(283402)
BG Mode:None Group 1 - Event 1

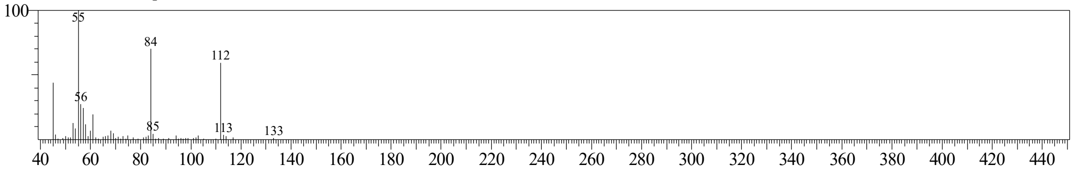

Hit#:4 Entry:3425 Library:NIST11.lib
SI:77 Formula:C6H8O2 CAS:3740-59-8 MolWeight:112 RetIndex:1017
CompName:3,4-Dihydro-6-methyl-2H-pyran-2-one $$ 6-Methyl-3,4-dihydro-2H-pyran-2-one # $$

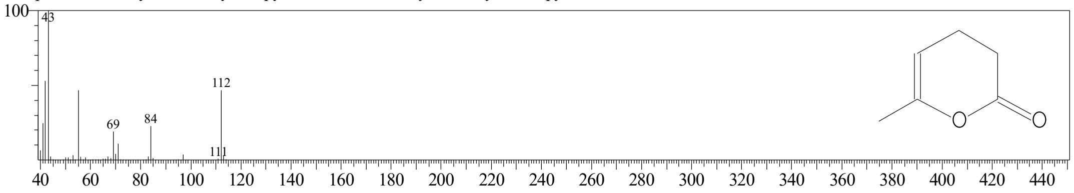

Hit#:5 Entry:7543 Library:NIST11s.lib
SI:77 Formula:C7H14N2O CAS:20917-49-1 MolWeight:142 RetIndex:1407
CompName:Azocine, octahydro-1-nitroso- $$ N-Nitrosoazacyclooctane $$ N-Nitrosoheptamethyleneimine $$ Nhmi $$ Nitrosoheptamethyleneimine $$ Nitroso-

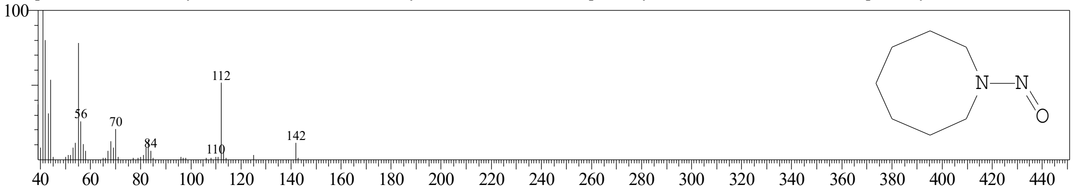

<< Target >>
Line#:2 R.Time:5.700(Scan#:25) MassPeaks:39
RawMode:Single 5.700(25) BasePeak:95.95(179925)
BG Mode:None Group 1 - Event 1

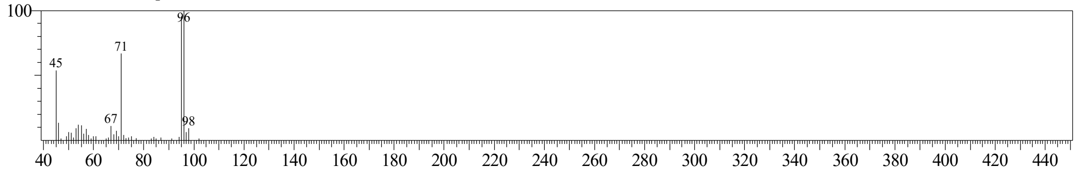

Hit#:1 Entry:61855 Library:NIST11.lib
SI:78 Formula:C11H14O5 CAS:6289-73-2 MolWeight:226 RetIndex:1509
CompName:2-Furanmethanediol, dipropionate $$ 2-Furyl(propionyloxy)methyl propionate # $$

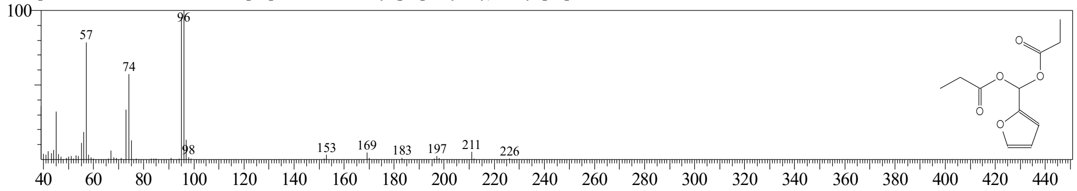

Hit#:2 Entry:1387 Library:NIST11.lib
SI:77 Formula:C5H8N2 CAS:2302-39-8 MolWeight:96 RetIndex:927
CompName:1H-Imidazole, 4,5-dimethyl- $$ Imidazole, 4,5-dimethyl- $$ 4,5-Dimethyl-1H-imidazole # $$

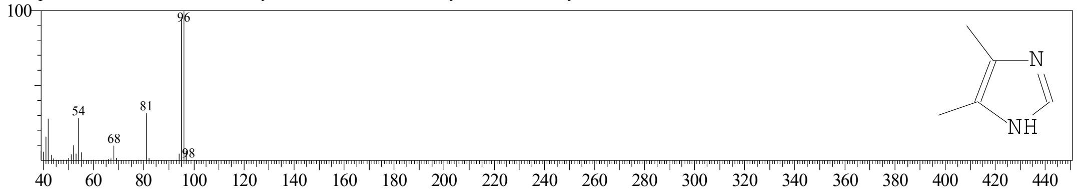

Hit#:3 Entry:1394 Library:NIST11s.lib
SI:77 Formula:C5H8N2 CAS:694-31-5 MolWeight:96 RetIndex:804
CompName:1H-Pyrazole, 1,5-dimethyl- $$ Pyrazole, 1,5-dimethyl- $$ 1,5-Dimethylpyrazole $$ 1,5-Dimethyl-1H-pyrazole $$

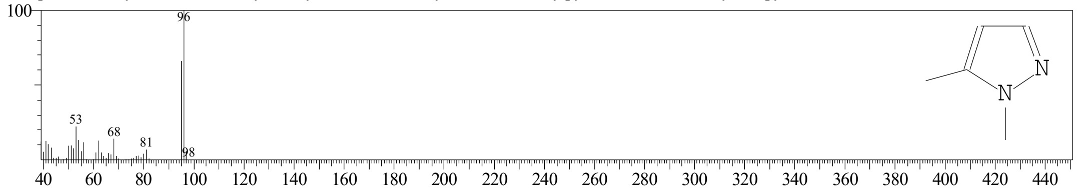

Hit#:4 Entry:17814 Library:NIST11.lib
SI:76 Formula:C6H9N3S CAS:1124-15-8 MolWeight:155 RetIndex:1523
CompName:1H-Pyrazole-1-carbothioamide, 3,5-dimethyl- $$ 3,5-Dimethyl-1H-pyrazole-1-carbothioamide # $$

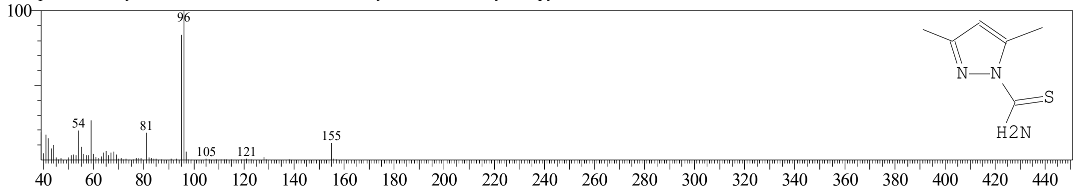

Hit#:5 Entry:51279 Library:NIST11.lib
SI:75 Formula:C10H13NO4 CAS:22882-18-4 MolWeight:211 RetIndex:1926
CompName:2(1H)-Pyridone, 1-(2-deoxy-.alpha.-D-erythro-pentofuranosyl)-

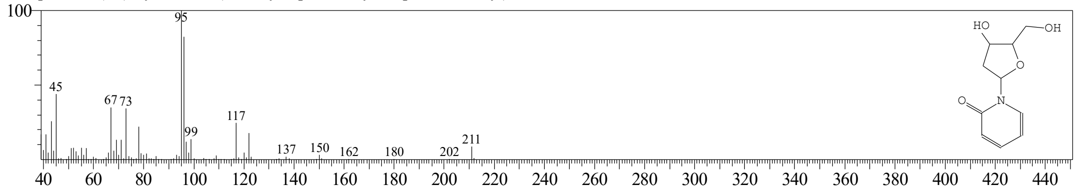

<< Target >>
Line#:3 R.Time:5.900(Scan#:49) MassPeaks:38
RawMode:Single 5.900(49) BasePeak:45.00(81993)
BG Mode:None Group 1 - Event 1

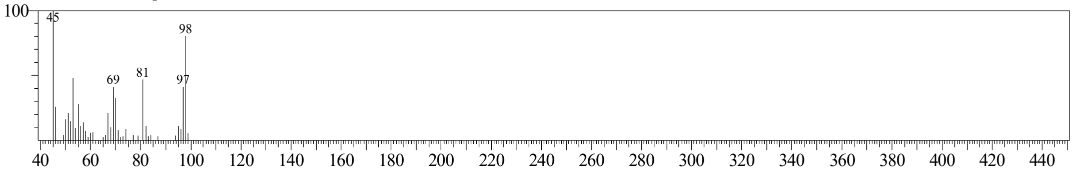

Hit#:1 Entry:1582 Library:NIST11.lib
SI:82 Formula:C5H6O2 CAS:98-00-0 MolWeight:98 RetIndex:885
CompName:2-Furanmethanol $$ Furfuryl alcohol $$ .alpha.-Furfuryl alcohol $$ .alpha.-Furylcarbinol $$ Furfuralcohol $$ Furyl alcohol $$ Furylcarbinol $$ 2-(

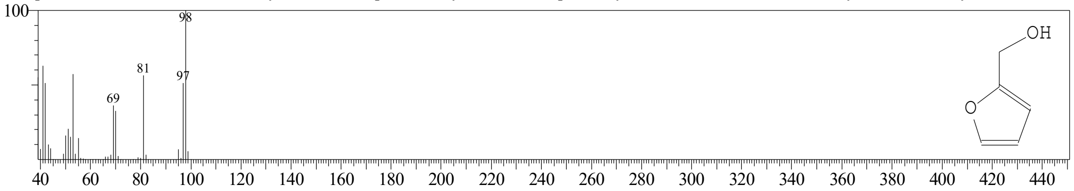

Hit#:2 Entry:1509 Library:NIST11s.lib
SI:81 Formula:C5H6O2 CAS:98-00-0 MolWeight:98 RetIndex:885
CompName:2-Furanmethanol $$ Furfuryl alcohol $$ .alpha.-Furfuryl alcohol $$ .alpha.-Furylcarbinol $$ Furfuralcohol $$ Furyl alcohol $$ Furylcarbinol $$ 2-(

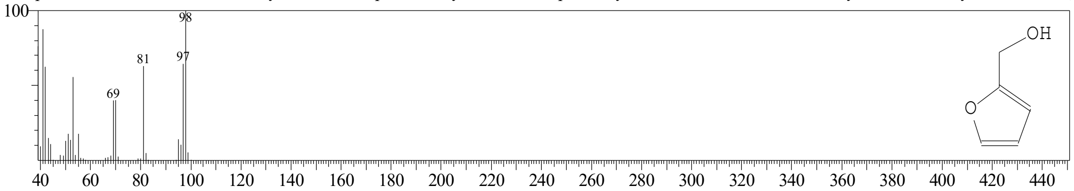

Hit#:3 Entry:1511 Library:NIST11s.lib
SI:81 Formula:C5H6O2 CAS:98-00-0 MolWeight:98 RetIndex:885
CompName:2-Furanmethanol $$ Furfuryl alcohol $$ .alpha.-Furfuryl alcohol $$ .alpha.-Furylcarbinol $$ Furfuralcohol $$ Furyl alcohol $$ Furylcarbinol $$ 2-(

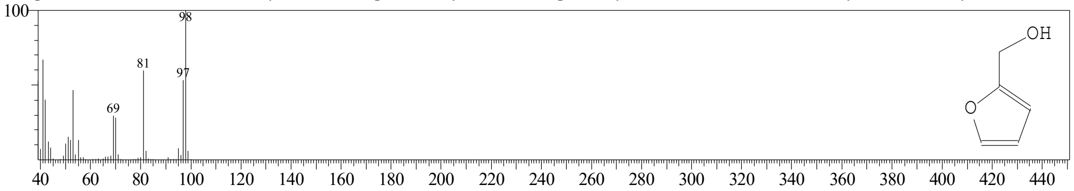

Hit#:4 Entry:1512 Library:NIST11s.lib
SI:80 Formula:C5H6O2 CAS:98-00-0 MolWeight:98 RetIndex:885
CompName:2-Furanmethanol $$ Furfuryl alcohol $$ .alpha.-Furfuryl alcohol $$ .alpha.-Furylcarbinol $$ Furfuralcohol $$ Furyl alcohol $$ Furylcarbinol $$ 2-(

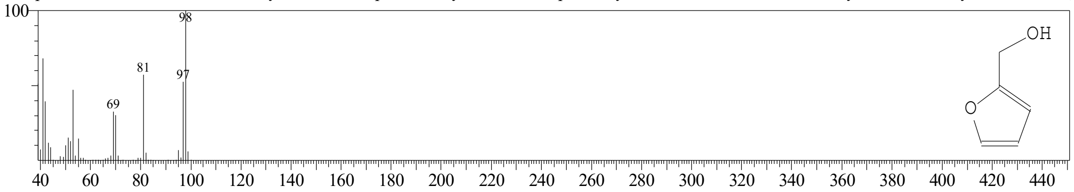

Hit#:5 Entry:1552 Library:NIST11.lib
SI:78 Formula:C4H6N2O CAS:10552-94-0 MolWeight:98 RetIndex:1029
CompName:1H-Pyrrole, 2,5-dihydro-1-nitroso- $$ 3-Pyrroline, 1-nitroso- $$ Nitroso-3-pyrroline $$ 3-Pyrroline, N-nitroso- $$ 2,5-Dihydro-1-nitroso-1H-pyrrol

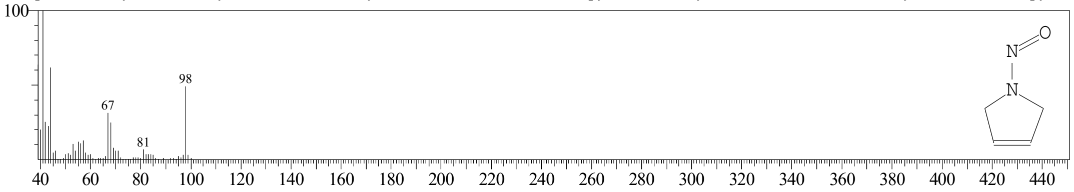

<< Target >>
Line#:4 R.Time:7.450(Scan#:235) MassPeaks:48
RawMode:Single 7.450(235) BasePeak:45.00(131030)
BG Mode:None Group 1 - Event 1

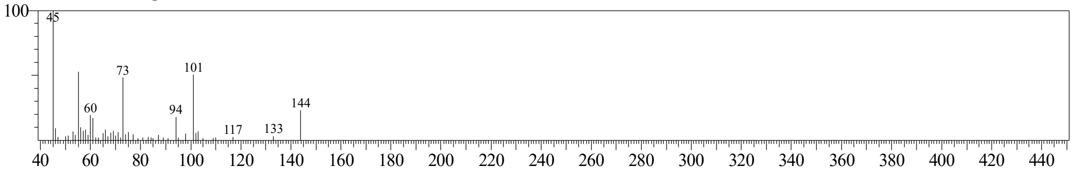

Hit#:1 Entry:8130 Library:NIST11s.lib
SI:75 Formula:C5H6O5 CAS:328-50-7 MolWeight:146 RetIndex:1367
CompName:2-Oxopentanedioic acid $$ 2-Ketoglutaric acid $$ 2-Oxoglutaric acid $$ .alpha.-Ketoglutaric acid $$ Pentanedioic acid, 2-oxo- $$ Glutaric acid, 2-c

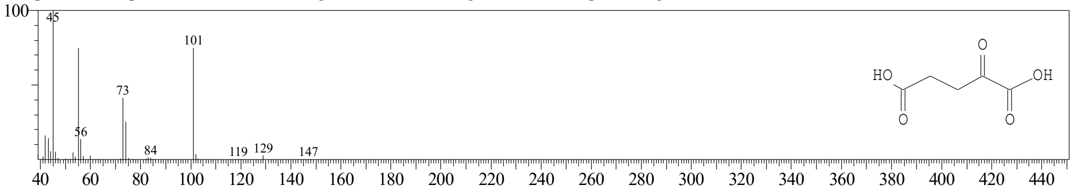

Hit#:2 Entry:19227 Library:NIST11.lib
SI:73 Formula:C8H14OS CAS:104223-43-0 MolWeight:158 RetIndex:1271
CompName:5-Butyldihydro-2(3H)thiophenone

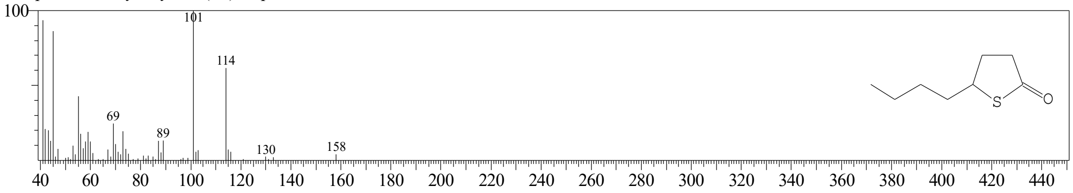

Hit#:3 Entry:18197 Library:NIST11.lib
SI:72 Formula:C7H8O4 CAS:189459-95-8 MolWeight:156 RetIndex:1239
CompName:Butanedioic acid, monopropargyl ester $$ 4-Oxo-4-(2-propynyloxy)butanoic acid # $$

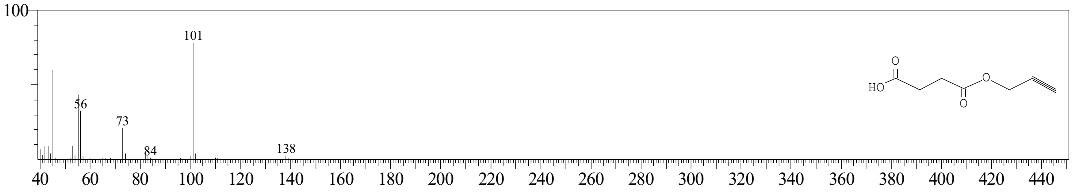

Hit#:4 Entry:5485 Library:NIST11s.lib
SI:72 Formula:C6H15BO2 CAS:53907-92-9 MolWeight:130 RetIndex:0
CompName:Boronic acid, ethyl-, diethyl ester $$ Diethyl ethylboronate # $$

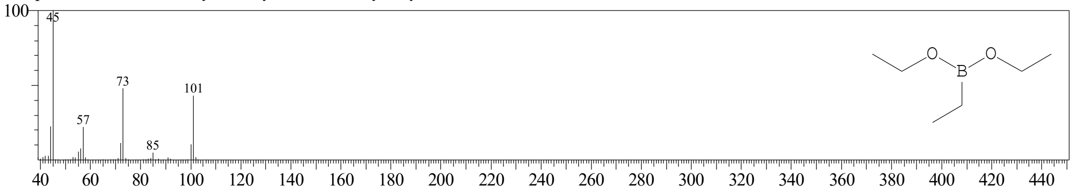

Hit#:5 Entry:13472 Library:NIST11.lib
SI:72 Formula:C5H6O5 CAS:328-50-7 MolWeight:146 RetIndex:1367
CompName:2-Oxopentanedioic acid $$ 2-Ketoglutaric acid $$ 2-Oxoglutaric acid $$ .alpha.-Ketoglutaric acid $$ Pentanedioic acid, 2-oxo- $$ Glutaric acid, 2-c

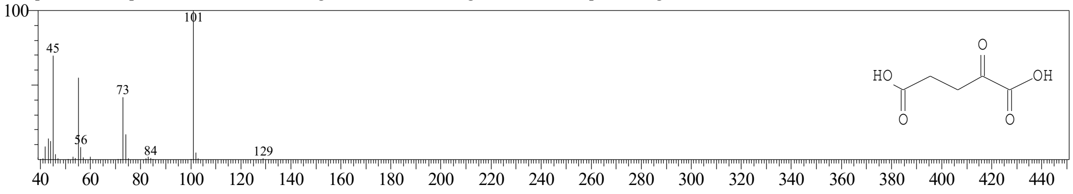

<< Target >>
Line#:5 R.Time:7.708(Scan#:266) MassPeaks:56
RawMode:Single 7.708(266) BasePeak:55.00(292359)
BG Mode:None Group 1 - Event 1

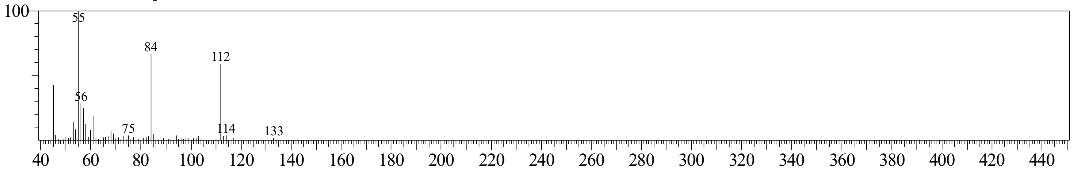

Hit#:1 Entry:2882 Library:NIST11s.lib
SI:78 Formula:C6H8O2 CAS:504-02-9 MolWeight:112 RetIndex:1062
CompName:1,3-Cyclohexanedione $$ Dihydroresorcinol $$ Cyclohexane-1,3-dione $$ Hydroresorcinol $$ Resorcinol, dihydro- $$ 1,3-Benzenediol, dihydro- $

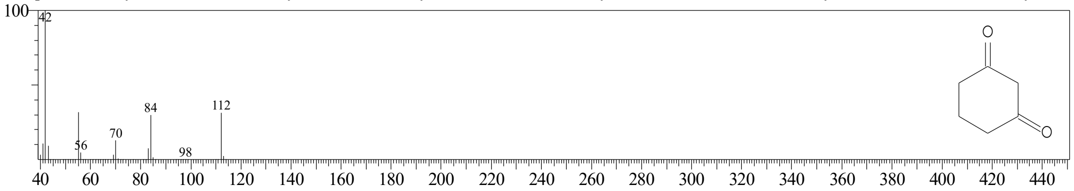

Hit#:2 Entry:2923 Library:NIST11s.lib
SI:78 Formula:C6H12N2 CAS:280-57-9 MolWeight:112 RetIndex:965
CompName:Triethylenediamine $$ 1,4-Diazabicyclo[2.2.2]octane $$ Bicyclo[2.2.2]-1,4-diazaoctane $$ D 33LV $$ Dabco $$ Dabco 33LV $$ N,N'-endo-Ethyler

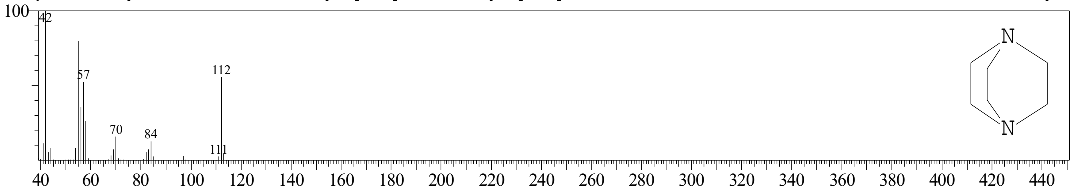

Hit#:3 Entry:2053 Library:NIST11s.lib
SI:78 Formula:C5H10O2 CAS:5057-99-8 MolWeight:102 RetIndex:976
CompName:1,2-Cyclopentanediol, trans- $$ trans-1,2-Cyclopentanediol $$ 1,2-Cyclopentanediol # $$

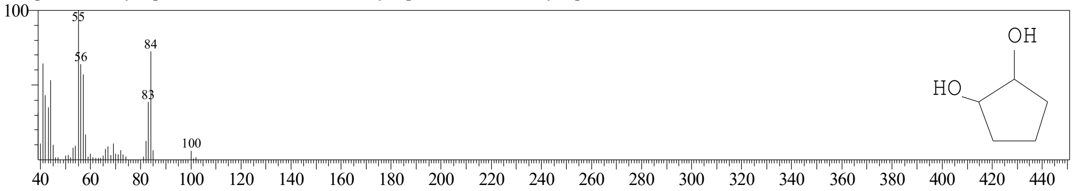

Hit#:4 Entry:2955 Library:NIST11s.lib
SI:78 Formula:C7H12O CAS:589-92-4 MolWeight:112 RetIndex:952
CompName:Cyclohexanone, 4-methyl- $$ 4-Methylcyclohexanone $$ p-Methyl cyclohexanone $$ 4-Methyl-1-cyclohexanone $$ Methyl-4 cyclohexanone-1 $$

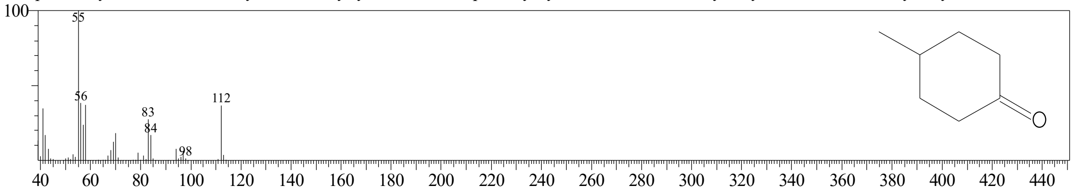

Hit#:5 Entry:3425 Library:NIST11.lib
SI:77 Formula:C6H8O2 CAS:3740-59-8 MolWeight:112 RetIndex:1017
CompName:3,4-Dihydro-6-methyl-2H-pyran-2-one $$ 6-Methyl-3,4-dihydro-2H-pyran-2-one # $$

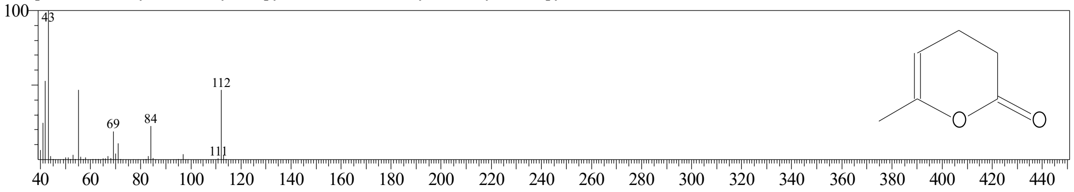

<< Target >>
Line#:6 R.Time:8.317(Scan#:339) MassPeaks:65
RawMode:Single 8.317(339) BasePeak:61.00(206081)
BG Mode:None Group 1 - Event 1

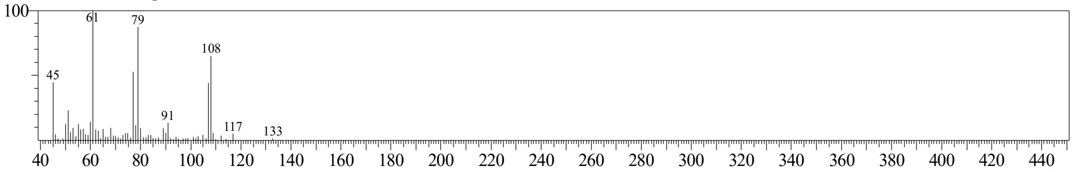

Hit#:1 Entry:94554 Library:NIST11.lib
SI:66 Formula:C13H18O6 CAS:0-00-0 MolWeight:270 RetIndex:2387
CompName:4-Benzyloxy-6-hydroxymethyl-tetrahydropyran-2,3,5-triol $$ 3-O-Benzylhexopyranose # $$

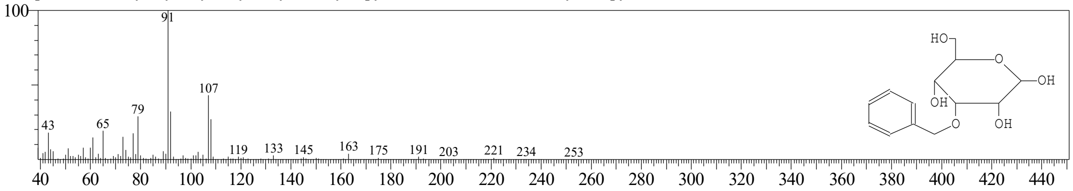

Hit#:2 Entry:60478 Library:NIST11.lib
SI:66 Formula:C12H16O2S CAS:62170-10-9 MolWeight:224 RetIndex:1753
CompName:S-Butyl phenoxythioacetate $$ S-Butyl phenoxyethanethioate # $$

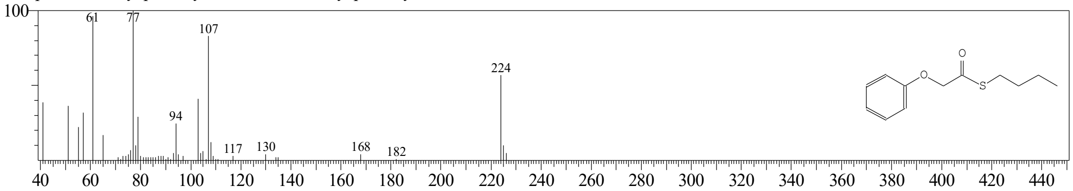

Cannot read library data

Hit#:4 Entry:45076 Library:NIST11.lib
SI:65 Formula:C11H26OSi CAS:167567-66-0 MolWeight:202 RetIndex:1075
CompName:1-Di(t-butyl)silyloxypropane $$ Di(tert-butyl)(propoxy)silane # $$

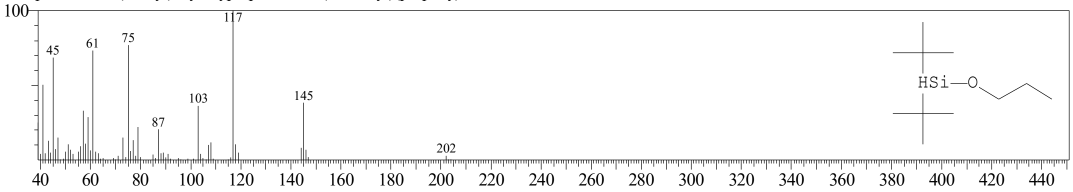

Hit#:5 Entry:2749 Library:NIST11.lib
SI:65 Formula:C3H8S2 CAS:1618-26-4 MolWeight:108 RetIndex:821
CompName:2,4-Dithiapentane $$ CH3SCH2SCH3 $$ Bis(methylthio)methane $$ Formaldehyde dimethyl mercaptal $$ Methane, bis(methylthio)- $$ Bis(methy

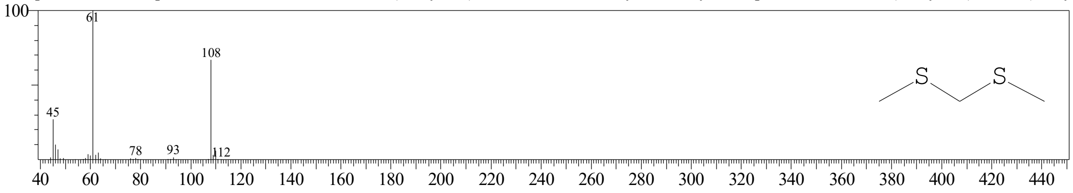

<< Target >>
Line#:7 R.Time:9.083(Scan#:431) MassPeaks:56
RawMode:Single 9.083(431) BasePeak:126.00(183738)
BG Mode:None Group 1 - Event 1

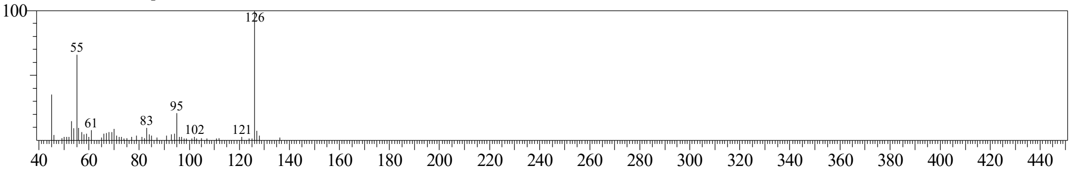

Hit#:1 Entry:10247 Library:NIST11s.lib
SI:81 Formula:C8H16N2O CAS:20917-50-4 MolWeight:156 RetIndex:1527
CompName:1H-Azonine, octahydro-1-nitroso- $$ N-Nitrosoazacyclononane $$ N-Nitrosooctamethyleneimine $$ Octahydro-1-nitroso-1H-azonine $$ 1-Nitroso

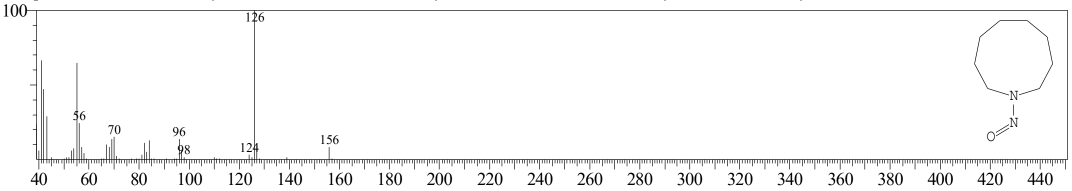

Hit#:2 Entry:18369 Library:NIST11.lib
SI:81 Formula:C8H16N2O CAS:20917-50-4 MolWeight:156 RetIndex:1527
CompName:1H-Azonine, octahydro-1-nitroso- $$ N-Nitrosoazacyclononane $$ N-Nitrosooctamethyleneimine $$ Octahydro-1-nitroso-1H-azonine $$ 1-Nitroso

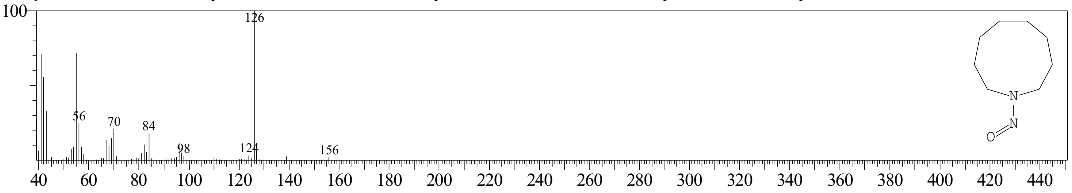

Hit#:3 Entry:24025 Library:NIST11.lib
SI:79 Formula:C11H21N CAS:108144-20-3 MolWeight:167 RetIndex:1217
CompName:Cyclohexanamine, N-3-butenyl-N-methyl- $$ N-(3-Butenyl)-N-methylcyclohexanamine # $$

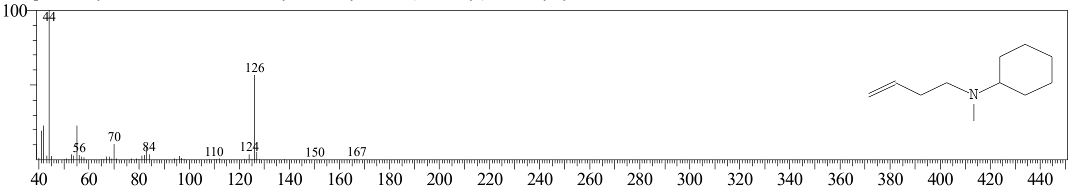

Hit#:4 Entry:26260 Library:NIST11.lib
SI:78 Formula:C7H9NO4 CAS:0-00-0 MolWeight:171 RetIndex:1401
CompName:5-Isoxazolepropanoic acid, 3-methoxy-

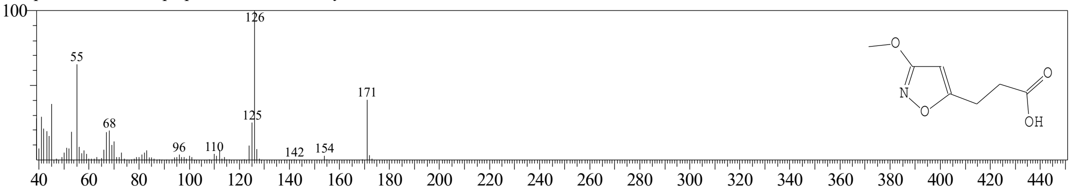

Hit#:5 Entry:4792 Library:NIST11s.lib
SI:76 Formula:C7H10O2 CAS:21835-01-8 MolWeight:126 RetIndex:1072
CompName:2-Cyclopenten-1-one, 3-ethyl-2-hydroxy- $$ 3-Ethyl-2-hydroxy-2-cyclopenten-1-one $$ 2-Cyclopenten-1-one, 2-hydroxy-3-ethyl $$ 2-Hydroxy-3-

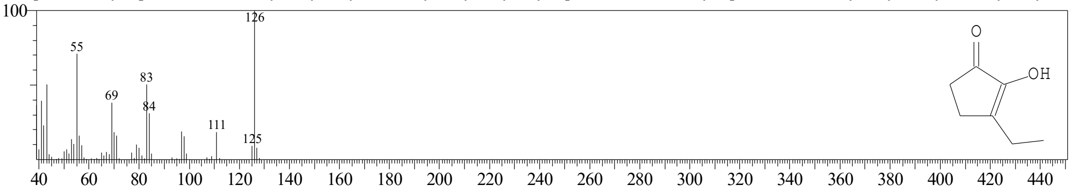

<< Target >>
Line#:8 R.Time:10.067(Scan#:549) MassPeaks:67
RawMode:Single 10.067(549) BasePeak:144.00(250134)
BG Mode:None Group 1 - Event 1

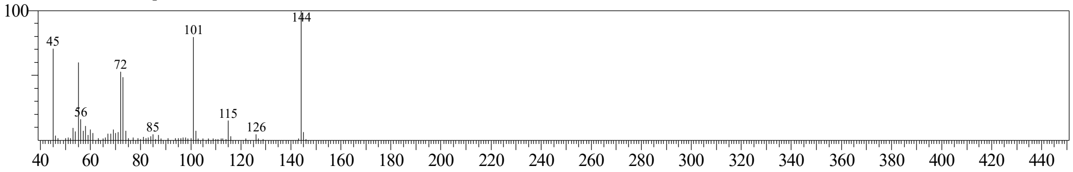

Hit#:1 Entry:12699 Library:NIST11.lib
SI:89 Formula:C6H8O4 CAS:28564-83-2 MolWeight:144 RetIndex:1269
CompName:4H-Pyran-4-one, 2,3-dihydro-3,5-dihydroxy-6-methyl- $$ 3,5-Dihydroxy-6-methyl-2,3-dihydro-4H-pyran-4-one $$ 2,3-dihydro-3,5-dihydroxy--6-m

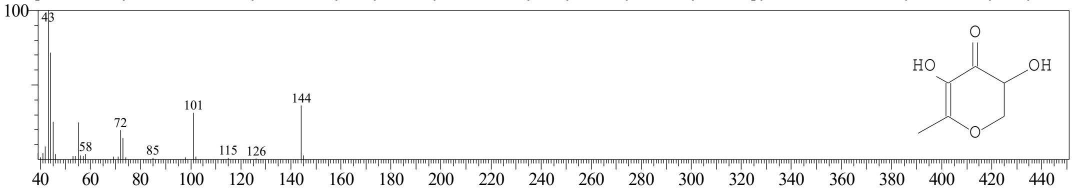

Hit#:2 Entry:7819 Library:NIST11s.lib
SI:86 Formula:C6H8O4 CAS:28564-83-2 MolWeight:144 RetIndex:1269
CompName:4H-Pyran-4-one, 2,3-dihydro-3,5-dihydroxy-6-methyl- $$ 3,5-Dihydroxy-6-methyl-2,3-dihydro-4H-pyran-4-one $$ 2,3-dihydro-3,5-dihydroxy--6-m

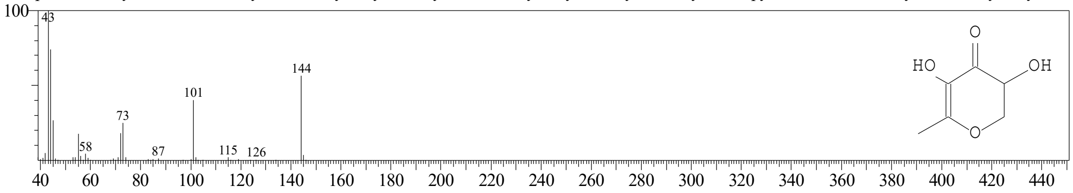

Hit#:3 Entry:45473 Library:NIST11.lib
SI:77 Formula:C9H17NO4 CAS:76683-94-8 MolWeight:203 RetIndex:1352
CompName:N-(1-Methoxycarbonyl-1-methylethyl)-4-methyl-2-aza-1,3-dioxane $$ Methyl 2-methyl-2-(4-methyl-1,3,2-dioxazinan-2-yl)propanoate # $$

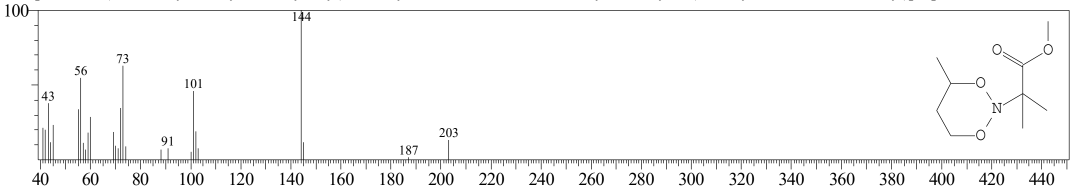

Hit#:4 Entry:4533 Library:NIST11.lib
SI:76 Formula:C6H12O2 CAS:3299-32-9 MolWeight:116 RetIndex:761
CompName:1,3-Dioxolane, 2,4,5-trimethyl- $$ 2,4,5-Trimethyl-1,3-dioxolane # $$

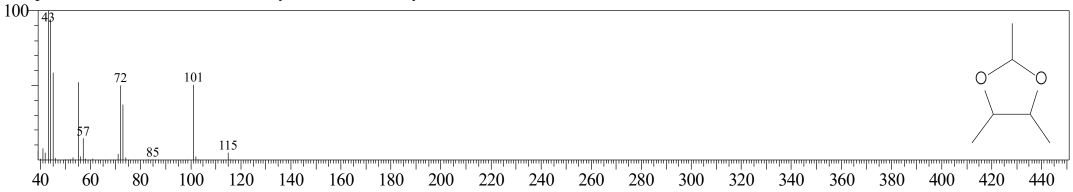

Hit#:5 Entry:13038 Library:NIST11.lib
SI:75 Formula:C8H16S CAS:17912-23-1 MolWeight:144 RetIndex:1120
CompName:2-n-Propylthiane $$ 2-Propyltetrahydro-2H-thiopyran $$ 2-Propyl-thiacyclohexane $$ Thiane, 2-propyl $$

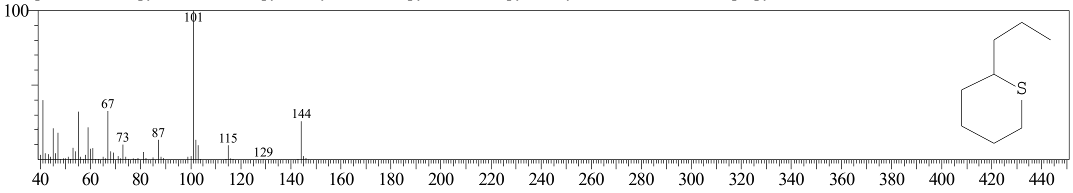

<< Target >>
Line#:9 R.Time:10.467(Scan#:597) MassPeaks:64
RawMode:Single 10.467(597) BasePeak:105.00(88407)
BG Mode:None Group 1 - Event 1

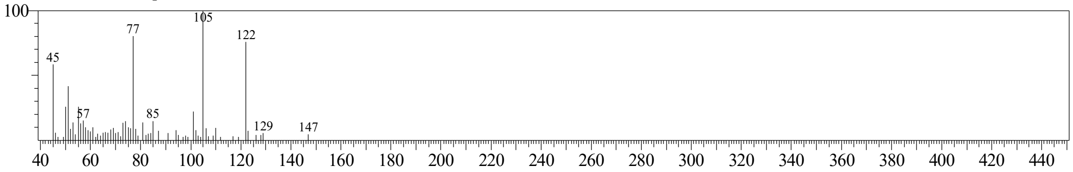

Hit#:1 Entry:4279 Library:NIST11s.lib
SI:75 Formula:C7H6O2 CAS:65-85-0 MolWeight:122 RetIndex:1150
CompName:Benzoic acid $$ Benzenecarboxylic acid $$ Benzeneformic acid $$ Benzenemethanoic acid $$ Benzoesaeure GK $$ Benzoesaeure GV $$ Carboxyb

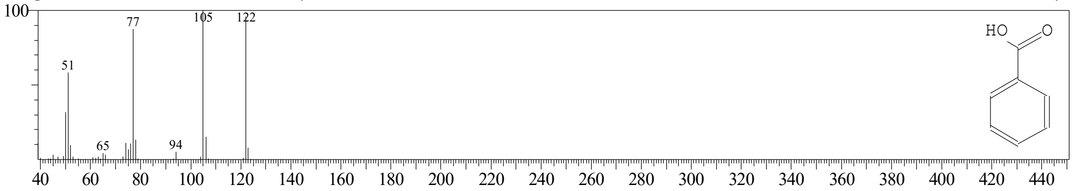

Hit#:2 Entry:174968 Library:NIST11.lib
SI:75 Formula:C20H20N2O6 CAS:0-00-0 MolWeight:384 RetIndex:3297
CompName:Hexanediamide, N,N'-di-benzoyloxy-

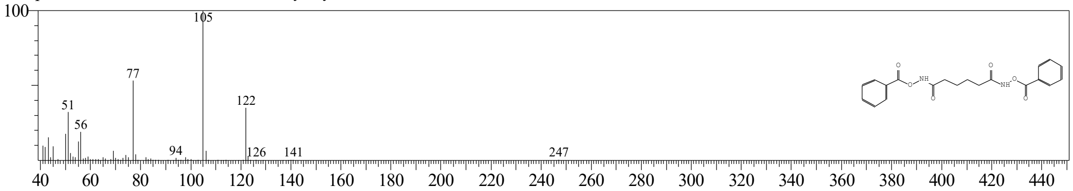

Hit#:3 Entry:4280 Library:NIST11s.lib
SI:74 Formula:C7H6O2 CAS:65-85-0 MolWeight:122 RetIndex:1150
CompName:Benzoic acid $$ Benzenecarboxylic acid $$ Benzeneformic acid $$ Benzenemethanoic acid $$ Benzoesaeure GK $$ Benzoesaeure GV $$ Carboxyb

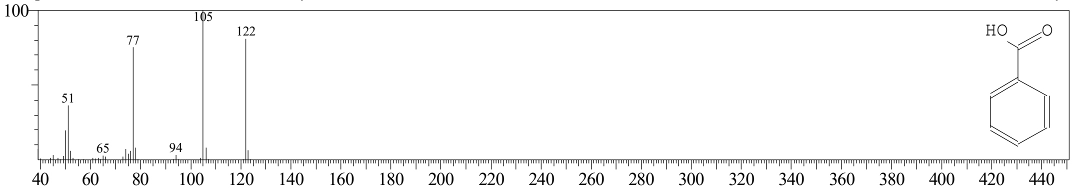

Hit#:4 Entry:181394 Library:NIST11.lib
SI:74 Formula:C21H22N2O6 CAS:0-00-0 MolWeight:398 RetIndex:3397
CompName:Heptanediamide, N,N'-di-benzoyloxy-

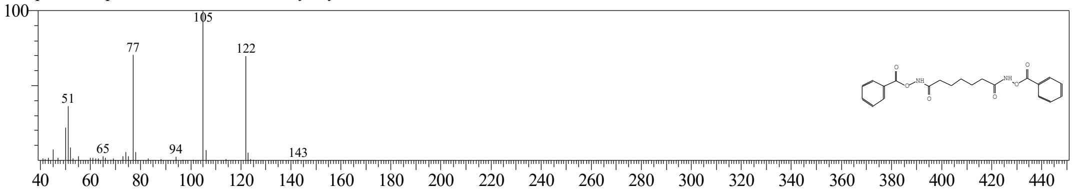

Hit#:5 Entry:173955 Library:NIST11.lib
SI:74 Formula:C20H18N2O6 CAS:0-00-0 MolWeight:382 RetIndex:3294
CompName:Cyclobutane-1,1-dicarboxamide, N,N'-di-benzoyloxy-

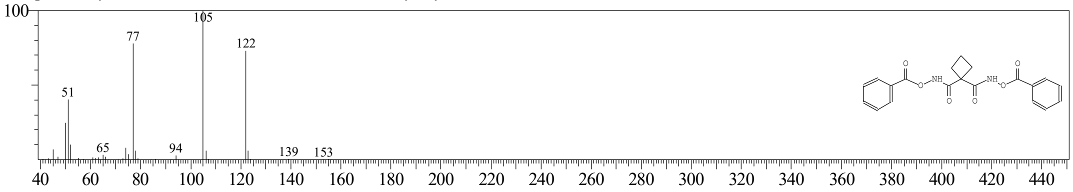

<< Target >>
Line#:10 R.Time:10.775(Scan#:634) MassPeaks:76
RawMode:Single 10.775(634) BasePeak:59.00(50630)
BG Mode:None Group 1 - Event 1

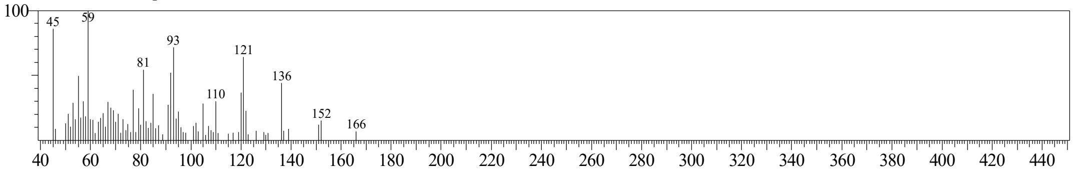

Hit#:1 Entry:17520 Library:NIST11.lib
SI:73 Formula:C10H18O CAS:98-55-5 MolWeight:154 RetIndex:1143
CompName:.alpha.-Terpineol $$ 3-Cyclohexene-1-methanol, .alpha.,.alpha.4-trimethyl- $$ p-Menth-1-en-8-ol $$ Terpineol schlechthin $$ Terpineol, .alpha. $$.

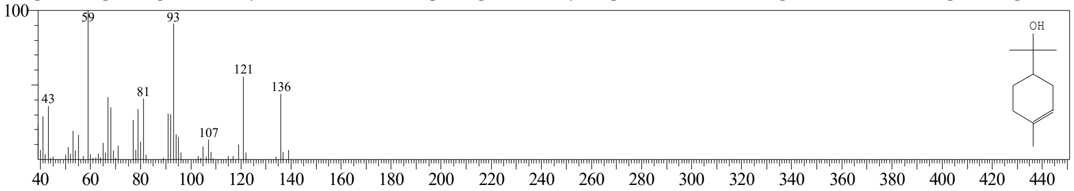

Hit#:2 Entry:17452 Library:NIST11.lib
SI:73 Formula:C10H18O CAS:38092-33-0 MolWeight:154 RetIndex:1100
CompName:trans-2,7-Dimethyl-3,6-octadien-2-ol $$ (3E)-2,7-Dimethyl-3,6-octadien-2-ol $$ 3,6-Octadien-2-ol, 2,7-dimethyl, (E) $$ 3,6-Octadien-2-ol, 2,7-dim

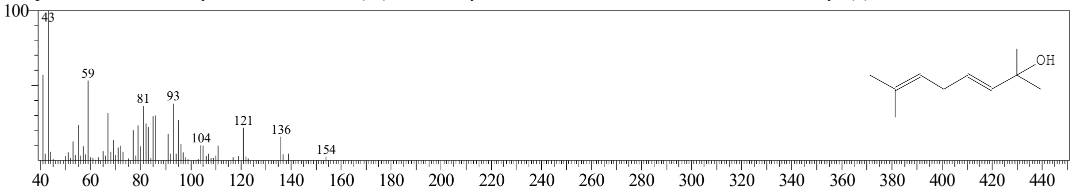

Hit#:3 Entry:17614 Library:NIST11.lib
SI:72 Formula:C10H18O CAS:4017-88-3 MolWeight:154 RetIndex:1125
CompName:Bicyclo[4.1.0]heptan-3-ol, 4,7,7-trimethyl-, [1R-(1.alpha.,3.alpha.,4.beta.,6.alpha.)]- $$ 4-Caranol, (1S,3R,4R,6R)-(-)- $$ 4,7,7-Trimethylbicyclo[4

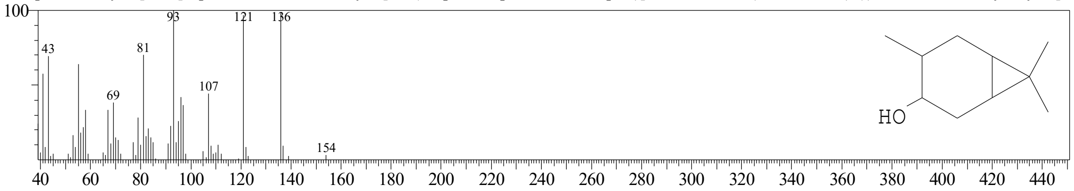

Hit#:4 Entry:17613 Library:NIST11.lib
SI:72 Formula:C10H18O CAS:38748-96-8 MolWeight:154 RetIndex:1125
CompName:Bicyclo[4.1.0]heptan-3-ol, 4,7,7-trimethyl-, (1.alpha.,3.alpha.,4.beta.,6.alpha.)- $$ 4,7,7-Trimethylbicyclo[4.1.0]heptan-3-ol, (1.alpha.,3.alpha.,4.be

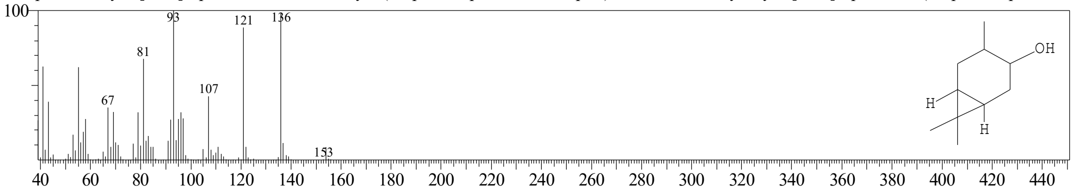

Hit#:5 Entry:17674 Library:NIST11.lib
SI:71 Formula:C10H18O CAS:4017-93-0 MolWeight:154 RetIndex:1125
CompName:Bicyclo[4.1.0]heptan-3-ol, 4,7,7-trimethyl-, [1R-(1.alpha.,3.beta.,4.beta.,6.alpha.)]- $$ 4-Caranol, (1S,3R,4S,6R)-(+)- $$ 4,7,7-Trimethylbicyclo[4.

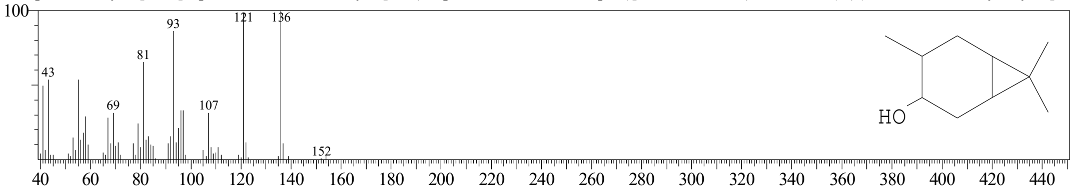

<< Target >>
Line#:11 R.Time:10.950(Scan#:655) MassPeaks:72
RawMode:Single 10.950(655) BasePeak:45.00(166054)
BG Mode:None Group 1 - Event 1

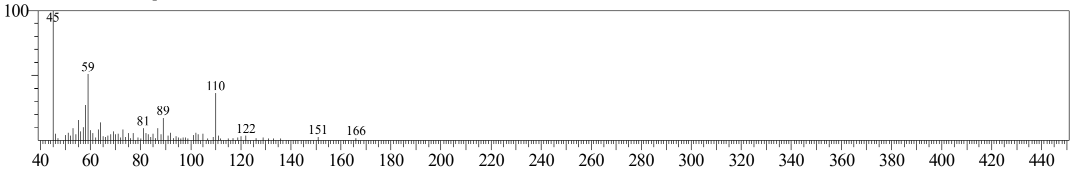

Hit#:1 Entry:14396 Library:NIST11.lib
SI:71 Formula:C7H16O3 CAS:37587-78-3 MolWeight:148 RetIndex:961
CompName:1-(Methoxymethoxy)-3-methyl-3-hydroxybutane $$ 4-(Methoxymethoxy)-2-methyl-2-butanol # $$

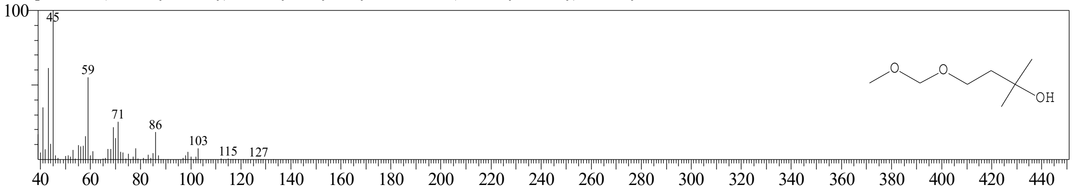

Hit#:2 Entry:69533 Library:NIST11.lib
SI:70 Formula:C9H19NO6 CAS:13096-67-8 MolWeight:237 RetIndex:1858
CompName:5-O-Methyl-d-gluconic acid dimethylamide

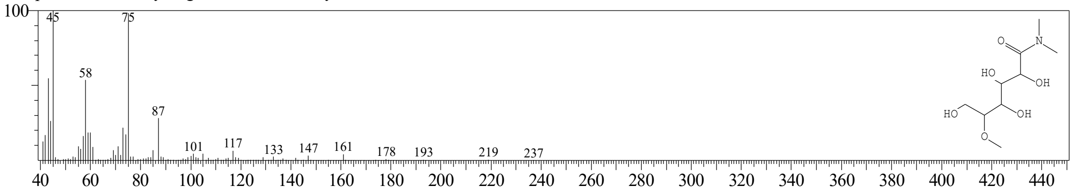

Hit#:3 Entry:22003 Library:NIST11.lib
SI:69 Formula:C7H16O4 CAS:112-35-6 MolWeight:164 RetIndex:1187
CompName:Ethanol, 2-[2-(2-methoxyethoxy)ethoxy]- $$ Dowanol TMAT $$ Methoxytriethylene glycol $$ Methoxytriglycol $$ Methyltrioxitol $$ Triethylene

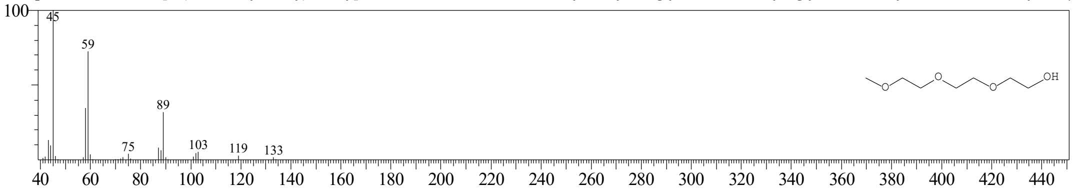

Hit#:4 Entry:163040 Library:NIST11.lib
SI:69 Formula:C20H42O5 CAS:5274-68-0 MolWeight:362 RetIndex:2556
CompName:Tetraethylene glycol monododecyl ether $$ Tetraethylene glycol mono-n-dodecyl ether $$ 3,6,9,12-Tetraoxatetracosan-1-ol $$ n-Dodecyl tetraethyle

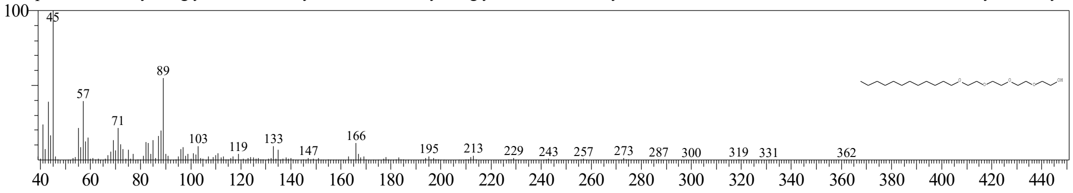

Hit#:5 Entry:29963 Library:NIST11.lib
SI:69 Formula:C8H18O4 CAS:3011-85-6 MolWeight:178 RetIndex:992
CompName:Butane, 1,2,3,4-tetramethoxy- $$ 1,2,3,4-Tetramethoxybutane # $$

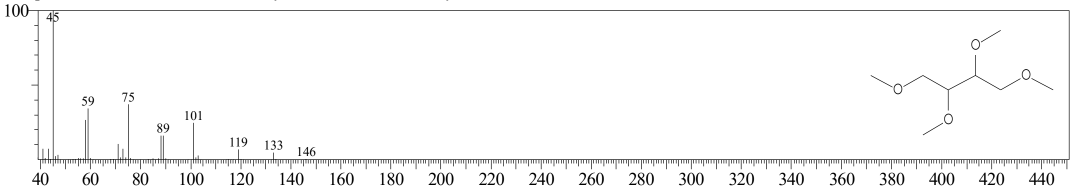

<< Target >>
Line#:12 R.Time:11.083(Scan#:671) MassPeaks:81
RawMode:Single 11.083(671) BasePeak:120.00(391295)
BG Mode:None Group 1 - Event 1

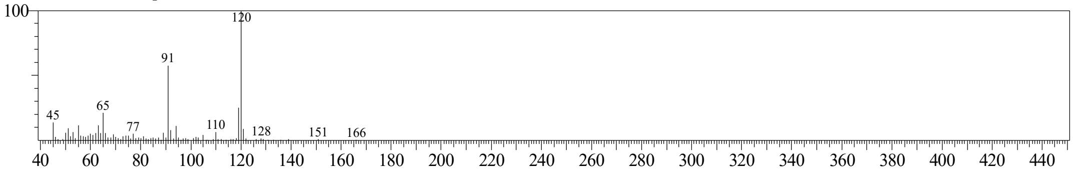

Hit#:1 Entry:4095 Library:NIST11s.lib
SI:85 Formula:C8H8O CAS:496-16-2 MolWeight:120 RetIndex:1036
CompName:Benzofuran, 2,3-dihydro- $$ Coumaran $$ Dihydrobenzofuran $$ Dihydrocoumarone $$ Kumaran $$ 2,3-Dihydrobenzofuran $$ 2,3-Dihydro-1-ben

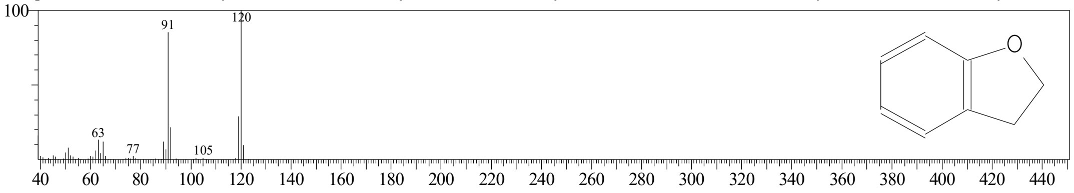

Hit#:2 Entry:5306 Library:NIST11.lib
SI:84 Formula:C8H8O CAS:496-16-2 MolWeight:120 RetIndex:1036
CompName:Benzofuran, 2,3-dihydro- $$ Coumaran $$ Dihydrobenzofuran $$ Dihydrocoumarone $$ Kumaran $$ 2,3-Dihydrobenzofuran $$ 2,3-Dihydro-1-ben

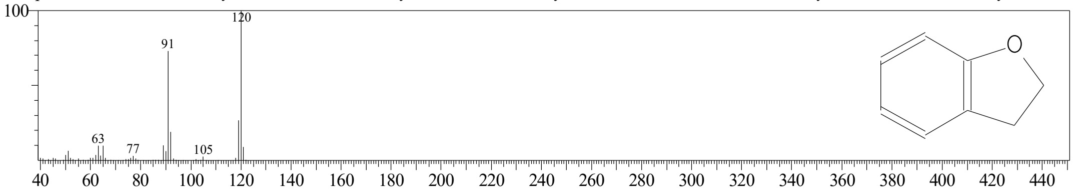

Hit#:3 Entry:4094 Library:NIST11s.lib
SI:81 Formula:C8H8O CAS:766-94-9 MolWeight:120 RetIndex:959
CompName:Benzene, (ethenyloxy)- $$ Ether, phenyl vinyl $$ (Vinyloxy)benzene $$ Phenoxyethene $$ Phenoxyethylene $$ Phenyl vinyl ether $$ Vinyl phenyl e

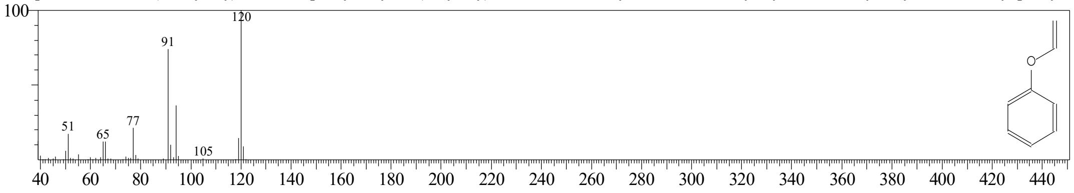

Hit#:4 Entry:14596 Library:NIST11.lib
SI:81 Formula:C10H12O CAS:5459-40-5 MolWeight:148 RetIndex:1172
CompName:4-Ethoxystyrene $$ p-Ethoxystyrene $$ Benzene, 1-ethenyl-4-ethoxy- $$ 1-Ethoxy-4-vinylbenzene # $$ p-Vinylphenetole $$

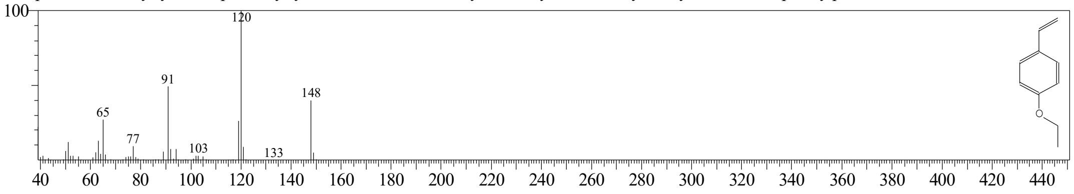

Hit#:5 Entry:5304 Library:NIST11.lib
SI:79 Formula:C8H8O CAS:766-94-9 MolWeight:120 RetIndex:959
CompName:Benzene, (ethenyloxy)- $$ Ether, phenyl vinyl $$ (Vinyloxy)benzene $$ Phenoxyethene $$ Phenoxyethylene $$ Phenyl vinyl ether $$ Vinyl phenyl e

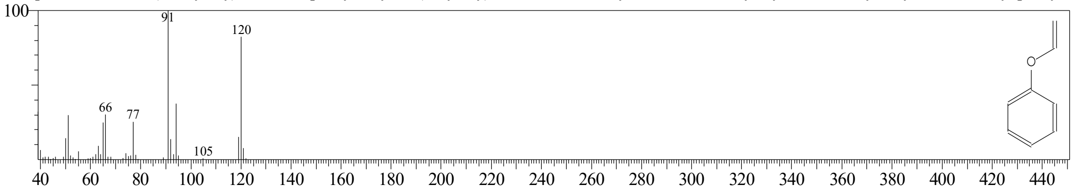

<< Target >>
Line#:13 R.Time:11.217(Scan#:687) MassPeaks:78
RawMode:Single 11.217(687) BasePeak:96.95(514053)
BG Mode:None Group 1 - Event 1

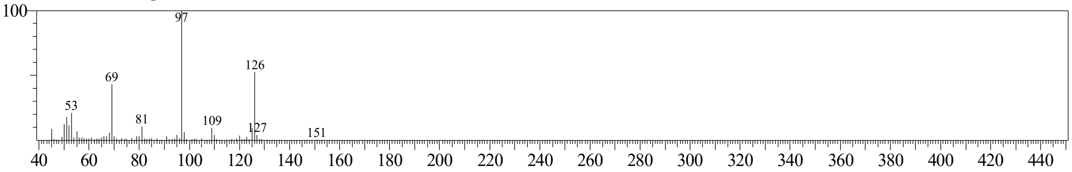

Hit#:1 Entry:6369 Library:NIST11.lib
SI:86 Formula:C6H6O3 CAS:67-47-0 MolWeight:126 RetIndex:1163
CompName:5-Hydroxymethylfurfural $$ 2-Furancarboxaldehyde, 5-(hydroxymethyl)- $$ 2-Furaldehyde, 5-(hydroxymethyl)- $$ HMF $$ 5-(Hyddroxymethyl)fu

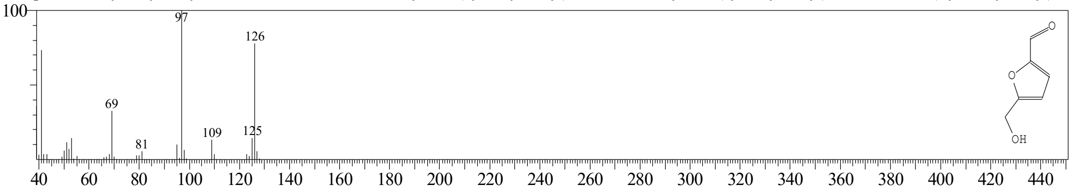

Hit#:2 Entry:4729 Library:NIST11s.lib
SI:85 Formula:C6H6O3 CAS:67-47-0 MolWeight:126 RetIndex:1163
CompName:5-Hydroxymethylfurfural $$ 2-Furancarboxaldehyde, 5-(hydroxymethyl)- $$ 2-Furaldehyde, 5-(hydroxymethyl)- $$ HMF $$ 5-(Hyddroxymethyl)fu

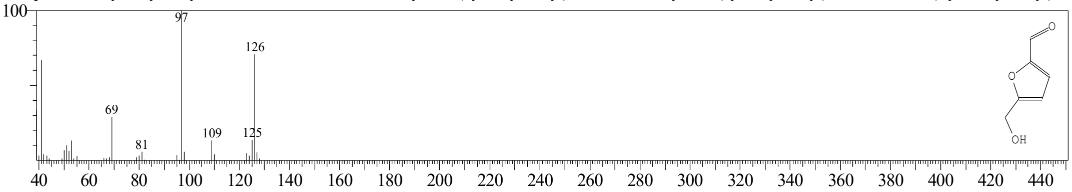

Hit#:3 Entry:6505 Library:NIST11.lib
SI:78 Formula:C8H14O CAS:17325-90-5 MolWeight:126 RetIndex:915
CompName:4-Hexen-3-one, 4,5-dimethyl- $$ 4,5-Dimethyl-4-hexen-3-one # $$

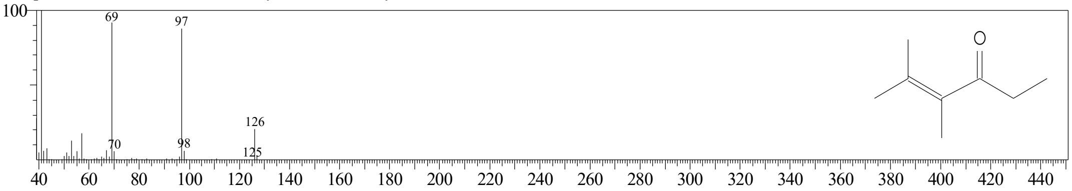

Hit#:4 Entry:6506 Library:NIST11.lib
SI:78 Formula:C8H14O CAS:22319-31-9 MolWeight:126 RetIndex:938
CompName:4-Hepten-3-one, 4-methyl- $$ (4E)-4-Methyl-4-hepten-3-one # $$

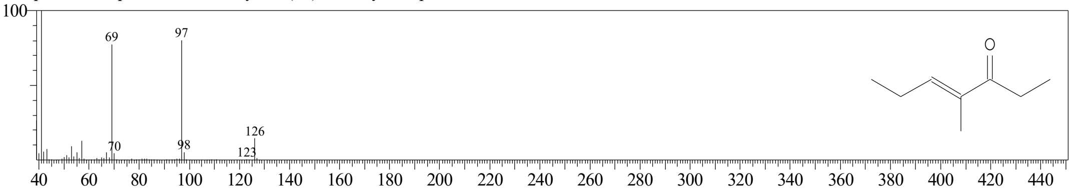

Hit#:5 Entry:4793 Library:NIST11s.lib
SI:78 Formula:C7H10S CAS:1551-27-5 MolWeight:126 RetIndex:993
CompName:Thiophene, 2-propyl- $$ 2-Propylthiophene $$ 2-n-Propylthiophene $$

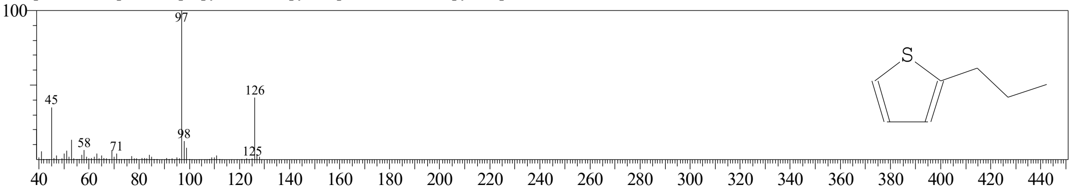

<< Target >>
Line#:14 R.Time:12.325(Scan#:820) MassPeaks:92
RawMode:Single 12.325(820) BasePeak:150.00(611714)
BG Mode:None Group 1 - Event 1

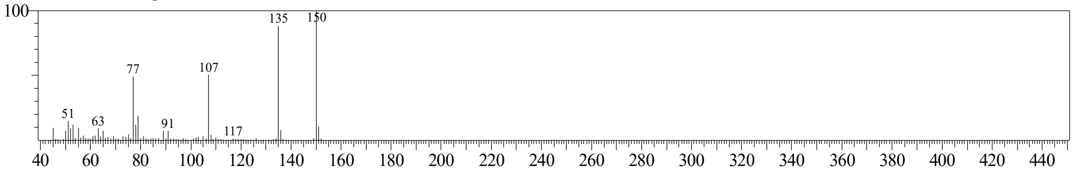

Hit#:1 Entry:15257 Library:NIST11.lib
SI:89 Formula:C9H10O2 CAS:7786-61-0 MolWeight:150 RetIndex:1293
CompName:2-Methoxy-4-vinylphenol $$ Phenol, 4-ethenyl-2-methoxy- $$ Phenol, 2-methoxy-4-vinyl- $$ 4-Hydroxy-3-methoxystyrene $$ p-Vinylguaiacol $$ 4

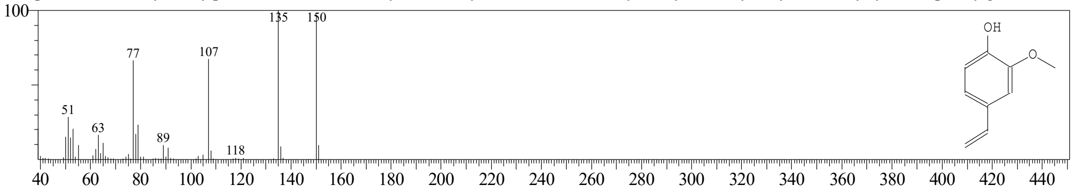

Hit#:2 Entry:8936 Library:NIST11s.lib
SI:81 Formula:C9H10O2 CAS:876-02-8 MolWeight:150 RetIndex:1363
CompName:4-Hydroxy-3-methylacetophenone $$ 4'-Hydroxy-3'-methylacetophenone $$ Ethanone, 1-(4-hydroxy-3-methylphenyl)- $$ 1-(4-Hydroxy-3-methylph

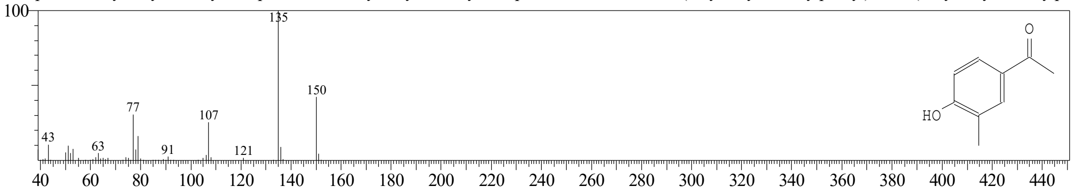

Hit#:3 Entry:8932 Library:NIST11s.lib
SI:81 Formula:C9H10O2 CAS:875-59-2 MolWeight:150 RetIndex:1363
CompName:4-Hydroxy-2-methylacetophenone $$ 2-Methyl-4-hydroxyacetophenone $$ Ethanone, 1-(4-hydroxy-2-methylphenyl)- $$ 1-(4-Hydroxy-2-methylpho

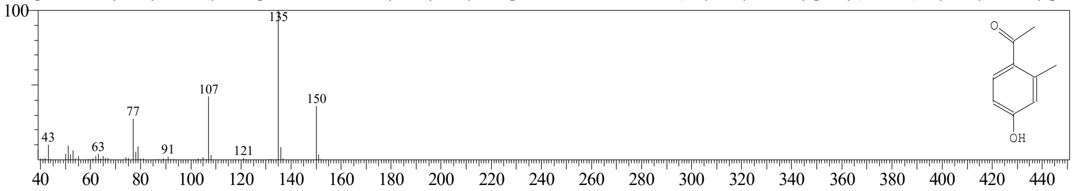

Hit#:4 Entry:8934 Library:NIST11s.lib
SI:81 Formula:C9H10O2 CAS:1450-72-2 MolWeight:150 RetIndex:1363
CompName:Ethanone, 1-(2-hydroxy-5-methylphenyl)- $$ 1-Hydroxy-2-acetyl-4-methylbenzene $$ 2-Hydroxy-5-methylacetophenone $$ 2'-Hydroxy-5'-methylac

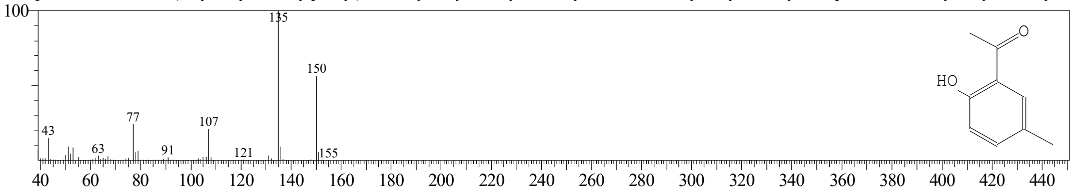

Hit#:5 Entry:15258 Library:NIST11.lib
SI:80 Formula:C9H10O2 CAS:586-37-8 MolWeight:150 RetIndex:1218
CompName:3-Methoxyacetophenone $$ Ethanone, 1-(3-methoxyphenyl)- $$ Acetophenone, 3'-methoxy- $$ 3-Acetylanisole $$ 1-Acetyl-3-methoxybenzene $$ r

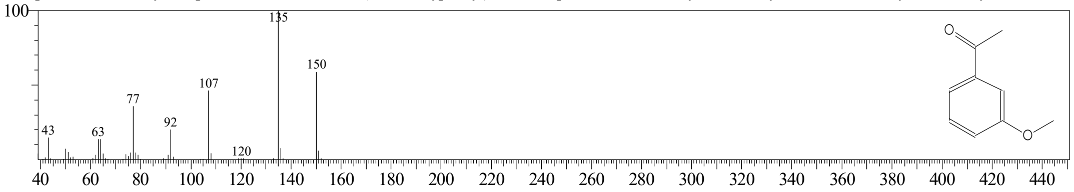

<< Target >>
Line#:15 R.Time:13.300(Scan#:937) MassPeaks:110
RawMode:Single 13.300(937) BasePeak:126.00(104366)
BG Mode:None Group 1 - Event 1

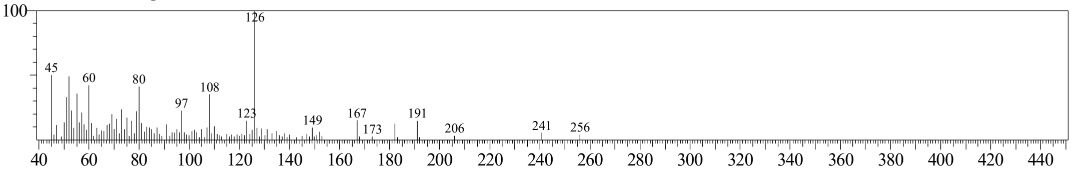

Hit#:1 Entry:4731 Library:NIST11s.lib
SI:68 Formula:C6H6O3 CAS:533-73-3 MolWeight:126 RetIndex:1342
CompName:1,2,4-Benzenetriol $$ 1,3,4-Benzenetriol $$ 2,5-Dihydroxyphenol $$ Hydroxyhydroquinone $$ 2-Hydroxy-1,4-hydroquinone $$ 1,2,4-Trihydroxyb

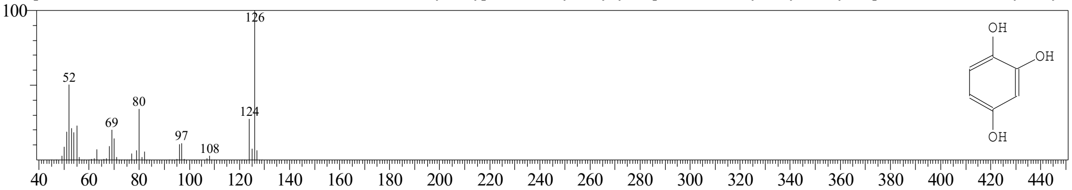

Hit#:2 Entry:17145 Library:NIST11.lib
SI:64 Formula:C8H10O3 CAS:85-42-7 MolWeight:154 RetIndex:1378
CompName:1,3-Isobenzofurandione, hexahydro- $$ 1,2-Cyclohexanedicarboxylic anhydride $$ Araldite HT 907 $$ Hexahydrophthalic acid anhydride $$ Hexah

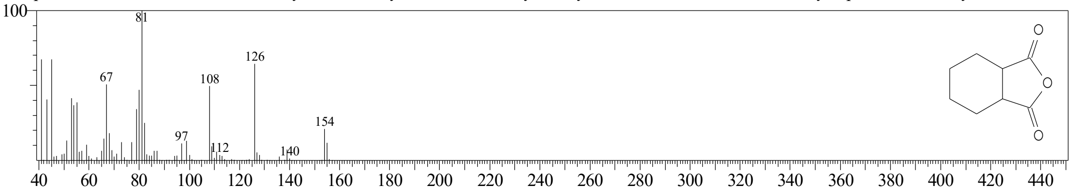

Hit#:3 Entry:6522 Library:NIST11.lib
SI:64 Formula:C8H14O CAS:3197-68-0 MolWeight:126 RetIndex:1053
CompName:Ethanol, 1-(1-cyclohexenyl)- $$ 1-(1-Cyclohexen-1-yl)ethanol # $$

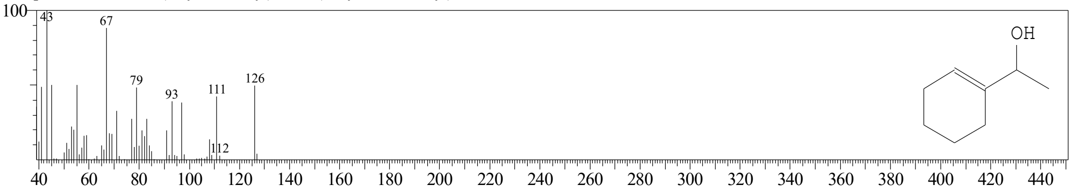

Hit#:4 Entry:12726 Library:NIST11.lib
SI:64 Formula:C6H8O4 CAS:0-00-0 MolWeight:144 RetIndex:1293
CompName:2,3-Dimethylfumaric acid $$ (2E)-2,3-Dimethyl-2-butenedioic acid # $$

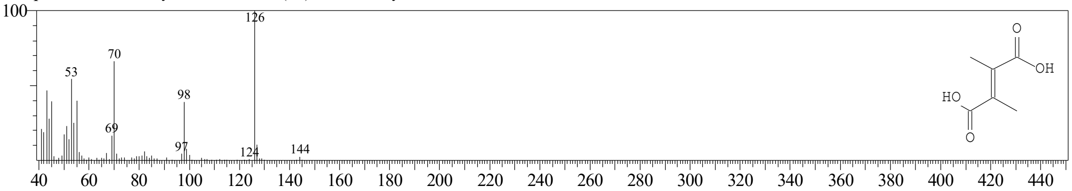

Hit#:5 Entry:43722 Library:NIST11.lib
SI:64 Formula:C10H16O4 CAS:0-00-0 MolWeight:200 RetIndex:1651
CompName:4a-(1-Hydroxy-ethyl)-hexahydrobenzo[1,3]dioxin-4-one $$ 4a-(1-Hydroxyethyl)hexahydro-4H-1,3-benzodioxin-4-one # $$

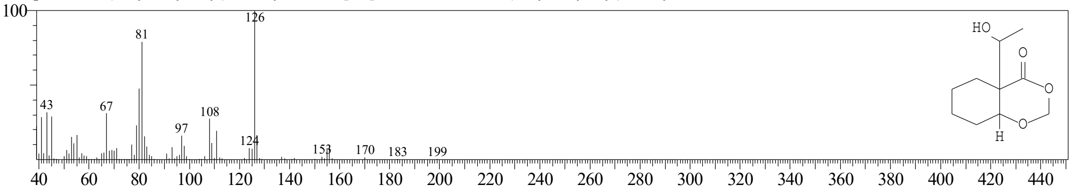

<< Target >>
Line#:16 R.Time:13.917(Scan#:1011) MassPeaks:115
RawMode:Single 13.917(1011) BasePeak:136.00(407423)
BG Mode:None Group 1 - Event 1

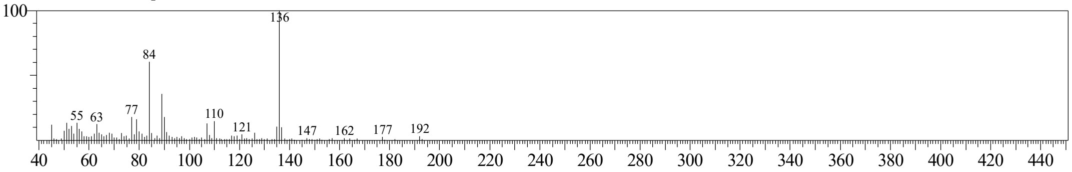

Hit#:1 Entry:11540 Library:NIST11s.lib
SI:70 Formula:C9H8O3 CAS:7310-95-4 MolWeight:164 RetIndex:1617
CompName:2-Hydroxy-5-methylisophthalaldehyde $$ 1,3-Benzenedicarboxaldehyde, 2-hydroxy-5-methyl- $$

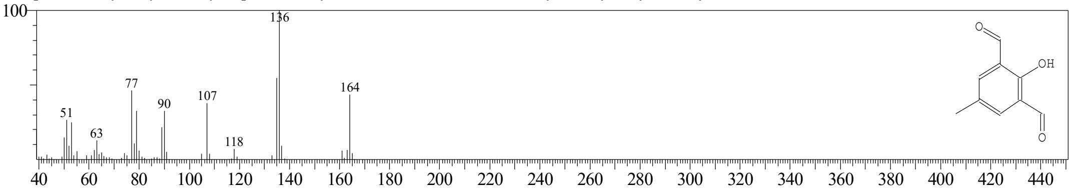

Hit#:2 Entry:22854 Library:NIST11.lib
SI:68 Formula:C10H15NO CAS:39590-27-7 MolWeight:165 RetIndex:1425
CompName:2-Ethoxyphenethylamine $$ 2-(2-Ethoxyphenyl)ethanamine # $$

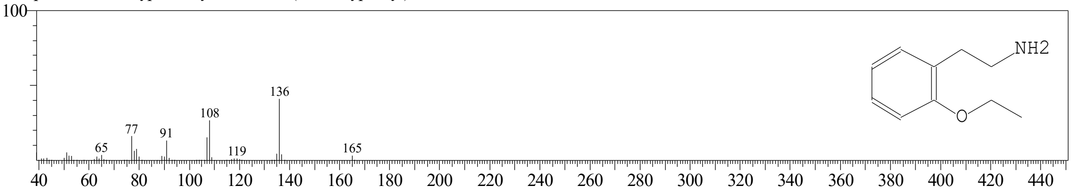

Hit#:3 Entry:24227 Library:NIST11.lib
SI:68 Formula:C8H8O4 CAS:2411-83-8 MolWeight:168 RetIndex:1502
CompName:Benzoic acid, 2,3-dihydroxy-, methyl ester $$ o-Pyrocatechuic acid, methyl ester $$ Methyl 2,3-dihydroxybenzoate $$

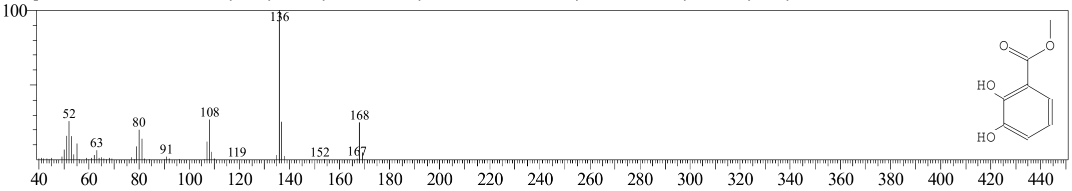

Hit#:4 Entry:9482 Library:NIST11.lib
SI:68 Formula:C7H8N2O CAS:0-00-0 MolWeight:136 RetIndex:1204
CompName:3H-Cyclopenta[c]pyridazin-3-one, 2,5,6,7-tetrahydro-

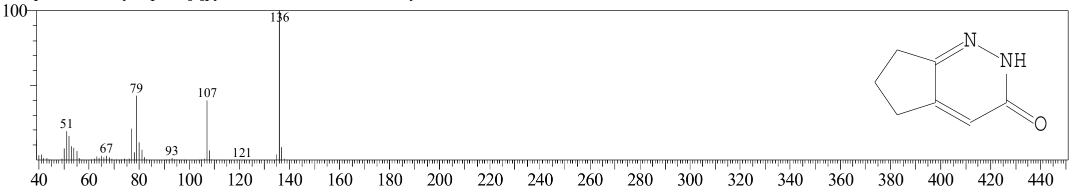

Hit#:5 Entry:9536 Library:NIST11.lib
SI:68 Formula:C8H8O2 CAS:90111-15-2 MolWeight:136 RetIndex:1316
CompName:3-Hydroxy-2-methylbenzaldehyde

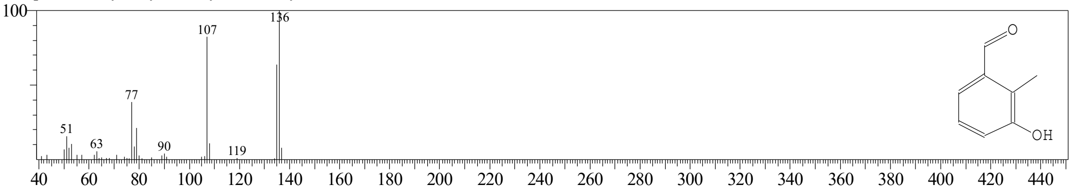

<< Target >>
Line#:17 R.Time:14.433(Scan#:1073) MassPeaks:123
RawMode:Single 14.433(1073) BasePeak:191.05(194669)
BG Mode:None Group 1 - Event 1

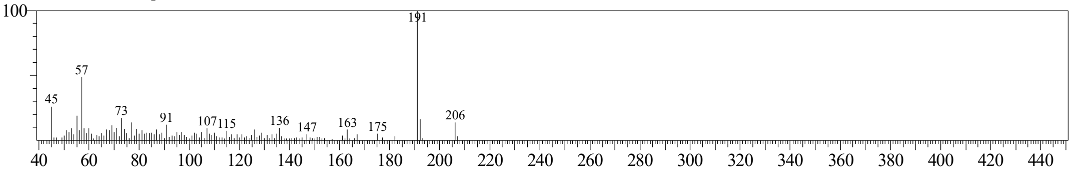

Hit#:1 Entry:163932 Library:NIST11.lib
SI:68 Formula:C14H20O9S CAS:0-00-0 MolWeight:364 RetIndex:3173
CompName:3-Tosyl sedoheptulose $$ 3-O-[(4-Methylphenyl)sulfonyl]hept-2-ulopyranose # $$

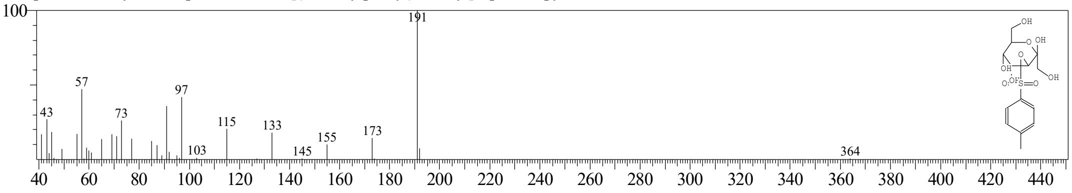

Hit#:2 Entry:18376 Library:NIST11s.lib
SI:67 Formula:C14H22O CAS:128-39-2 MolWeight:206 RetIndex:1555
CompName:Phenol, 2,6-bis(1,1-dimethylethyl)- $$ Phenol, 2,6-di-tert-butyl- $$ 2,6-Bis(tert-butyl)phenol $$ 2,6-Bis(1,1-dimethylethyl)phenol $$ 2,6-Di-tert-bu

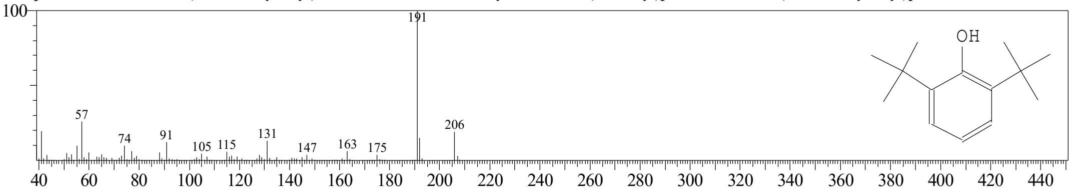

Hit#:3 Entry:18381 Library:NIST11s.lib
SI:66 Formula:C14H22O CAS:1138-52-9 MolWeight:206 RetIndex:1555
CompName:Phenol, 3,5-bis(1,1-dimethylethyl)- $$ Phenol, 3,5-di-tert-butyl- $$ 3,5-Di-tert-butylphenol $$ Phenol, 3,5-bis(t-butyl) $$ 3,5-Di-t-butylphenol $$ 3

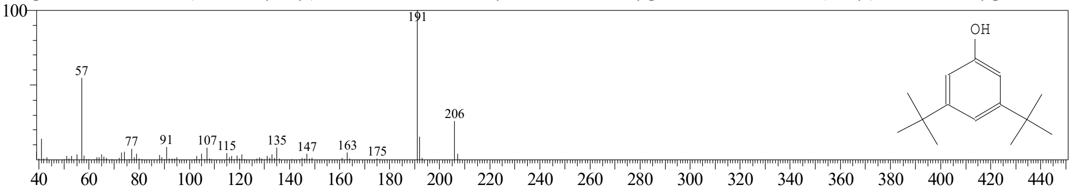

Hit#:4 Entry:18382 Library:NIST11s.lib
SI:66 Formula:C14H22O CAS:1138-52-9 MolWeight:206 RetIndex:1555
CompName:Phenol, 3,5-bis(1,1-dimethylethyl)- $$ Phenol, 3,5-di-tert-butyl- $$ 3,5-Di-tert-butylphenol $$ Phenol, 3,5-bis(t-butyl) $$ 3,5-Di-t-butylphenol $$ 3

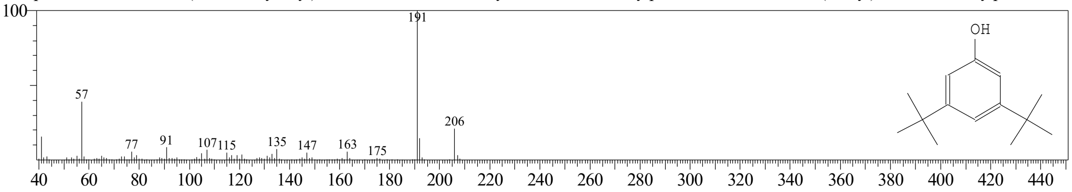

Hit#:5 Entry:18377 Library:NIST11s.lib
SI:65 Formula:C14H22O CAS:96-76-4 MolWeight:206 RetIndex:1555
CompName:Phenol, 2,4-bis(1,1-dimethylethyl)- $$ Phenol, 2,4-di-tert-butyl- $$ 2,4-Di-tert-butylphenol $$ 2,4-di-t-Butylphenol $$ 1-Hydroxy-2,4-di-tert-butyll

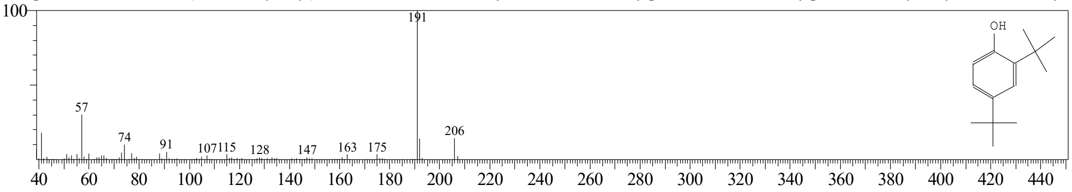

<< Target >>
Line#:18 R.Time:14.667(Scan#:1101) MassPeaks:128
RawMode:Single 14.667(1101) BasePeak:127.00(152826)
BG Mode:None Group 1 - Event 1

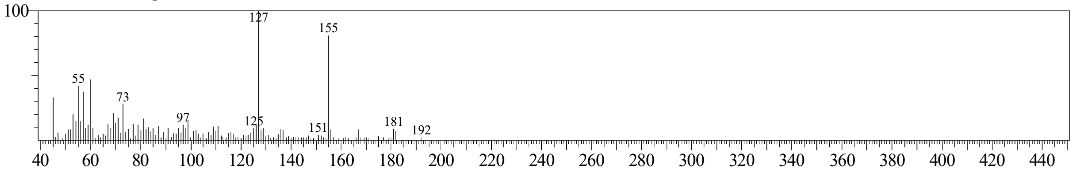

Hit#:1 Entry:24277 Library:NIST11s.lib
SI:72 Formula:C16H30O3 CAS:623-66-5 MolWeight:270 RetIndex:1915
CompName:Caprylic anhydride $$ Octanoic acid, anhydride $$ Octanoic anhydride $$

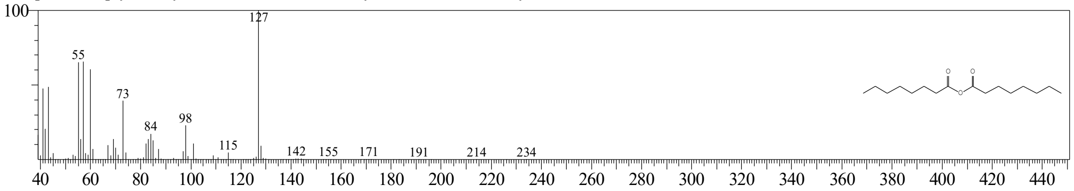

Hit#:2 Entry:63632 Library:NIST11.lib
SI:71 Formula:C13H24O3 CAS:2649-93-6 MolWeight:228 RetIndex:1669
CompName:Azelaaldehydic acid, butyl ester $$ Butyl 9-oxononanoate # $$

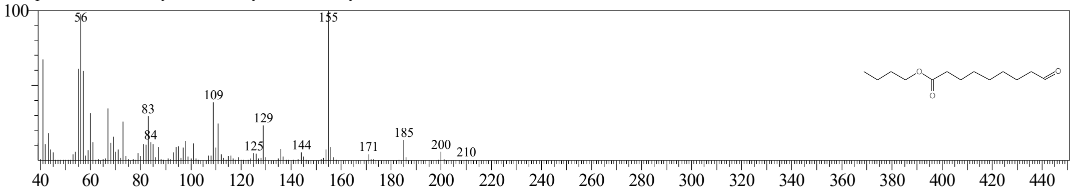

Hit#:3 Entry:53448 Library:NIST11.lib
SI:71 Formula:C12H22O3 CAS:34208-02-1 MolWeight:214 RetIndex:1506
CompName:Nonanoic acid, 9-oxo-, 1-methylethyl ester $$ Isopropyl 9-oxononanoate # $$

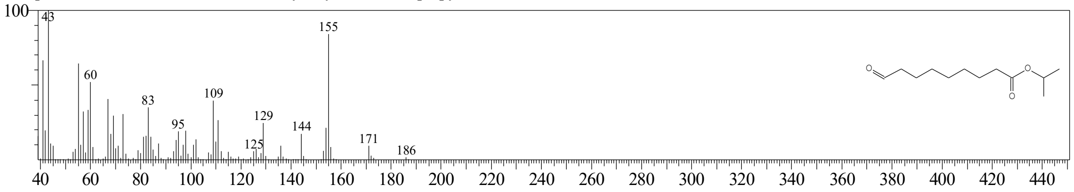

Hit#:4 Entry:43702 Library:NIST11.lib
SI:70 Formula:C10H16O4 CAS:13687-98-4 MolWeight:200 RetIndex:1354
CompName:2H-Pyran-2-carboxylic acid, 6-ethoxy-3,6-dihydro-, ethyl ester $$ Ethyl 6-ethoxy-3,6-dihydro-2H-pyran-2-carboxylate # $$

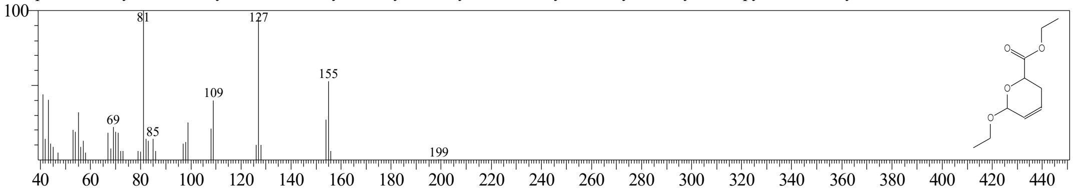

Hit#:5 Entry:34004 Library:NIST11.lib
SI:70 Formula:C11H24Si CAS:98213-23-1 MolWeight:184 RetIndex:1033
CompName:Silane, triethyl-1-pentenyl- $$ Triethyl[(1E)-1-pentenyl]silane # $$

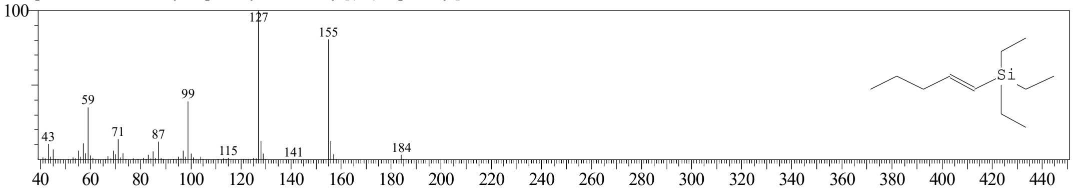

<< Target >>
Line#:19 R.Time:14.750(Scan#:1111) MassPeaks:124
RawMode:Single 14.750(1111) BasePeak:111.00(82951)
BG Mode:None Group 1 - Event 1

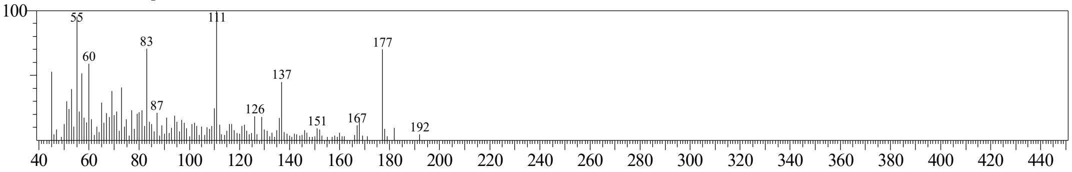

Hit#:1 Entry:6538 Library:NIST11.lib
SI:67 Formula:C8H14O CAS:0-00-0 MolWeight:126 RetIndex:1024
CompName:4,4-Dimethyl-cyclohex-2-en-1-ol $$ 4,4-Dimethyl-2-cyclohexen-1-ol # $$

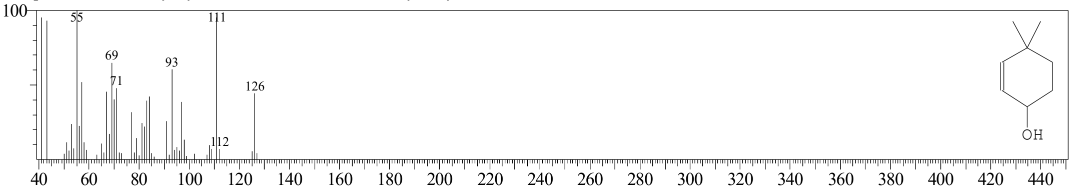

Hit#:2 Entry:81228 Library:NIST11.lib
SI:66 Formula:C16H28O2 CAS:459-67-6 MolWeight:252 RetIndex:1993
CompName:11-(2-Cyclopenten-1-yl)undecanoic acid, (+)- $$ Hydnocarpic acid $$ 2-Cyclopentene-1-undecanoic acid $$ Isolated from chaulmoogra oil $$ 11-(2

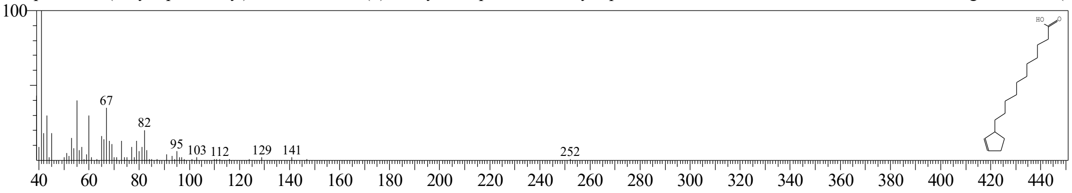

Hit#:3 Entry:82667 Library:NIST11.lib
SI:65 Formula:C16H30O2 CAS:6053-49-2 MolWeight:254 RetIndex:2011
CompName:Cyclopentaneundecanoic acid $$ 11-Cyclopentylundecanoic acid # $$

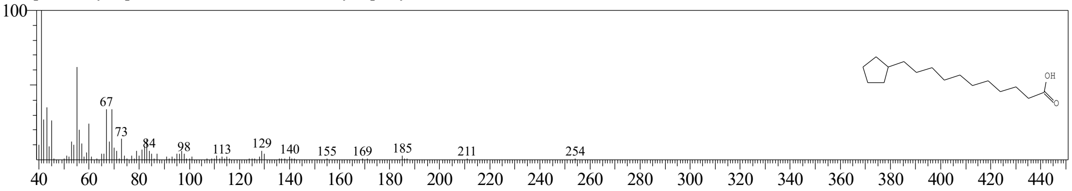

Hit#:4 Entry:51953 Library:NIST11.lib
SI:65 Formula:C11H16O4 CAS:117132-08-8 MolWeight:212 RetIndex:1610
CompName:9,9-Dimethoxybicyclo[3.3.1]nona-2,4-dione $$ 9,9-Dimethoxybicyclo[3.3.1]nonane-2,4-dione # $$

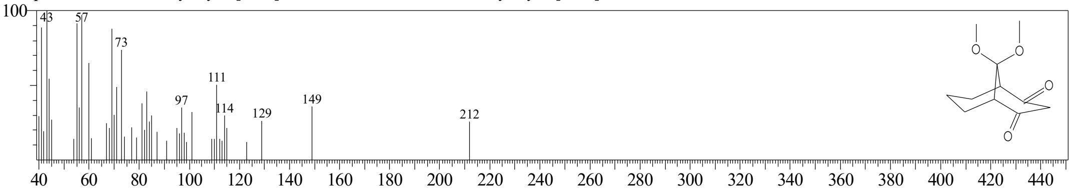

Hit#:5 Entry:25037 Library:NIST11s.lib
SI:63 Formula:C18H34O2 CAS:112-80-1 MolWeight:282 RetIndex:2175
CompName:Oleic Acid $$ 9-Octadecenoic acid (Z)- $$ .DELTA.9-cis-Oleic acid $$ cis-Oleic Acid $$ cis-9-Octadecenoic Acid $$ Emersol 211 $$ Emersol 220 \

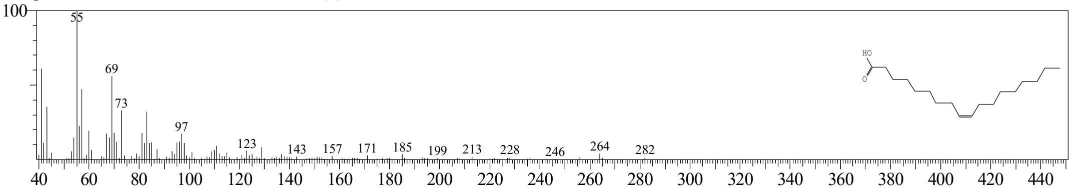

<< Target >>
Line#:20 R.Time:14.867(Scan#:1125) MassPeaks:120
RawMode:Single 14.867(1125) BasePeak:111.00(97066)
BG Mode:None Group 1 - Event 1

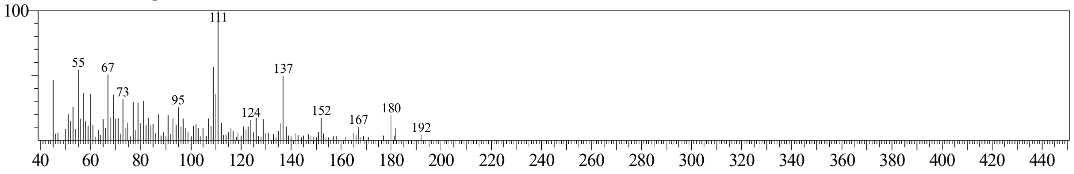

Hit#:1 Entry:17641 Library:NIST11.lib
SI:72 Formula:C10H18O CAS:6909-22-4 MolWeight:154 RetIndex:1125
CompName:5-Caranol, trans,trans-(+)- $$ 4,7,7-Trimethylbicyclo[4.1.0]heptan-2-ol # $$

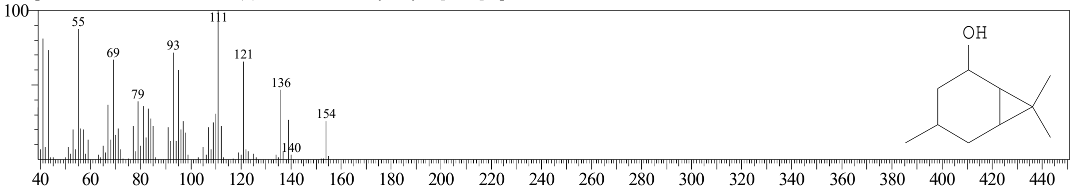

Hit#:2 Entry:25933 Library:NIST11.lib
SI:72 Formula:C10H18O2 CAS:13837-85-9 MolWeight:170 RetIndex:1326
CompName:Bicyclo[2.2.1]heptane-2,3-diol, 1,7,7-trimethyl-, (2-endo,3-exo)- $$ 2,3-Bornanediol, endo-2,exo-3- $$ 1,7,7-Trimethylbicyclo[2.2.1]heptane-2,3-d

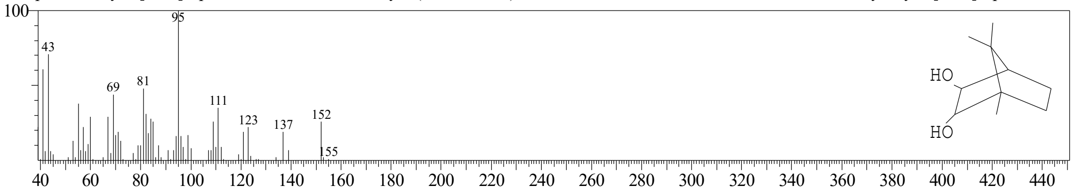

Hit#:3 Entry:17610 Library:NIST11.lib
SI:71 Formula:C10H18O CAS:6909-21-3 MolWeight:154 RetIndex:1125
CompName:5-Caranol, (1S,3R,5S,6R)-(-)- $$ 4,7,7-Trimethylbicyclo[4.1.0]heptan-2-ol # $$

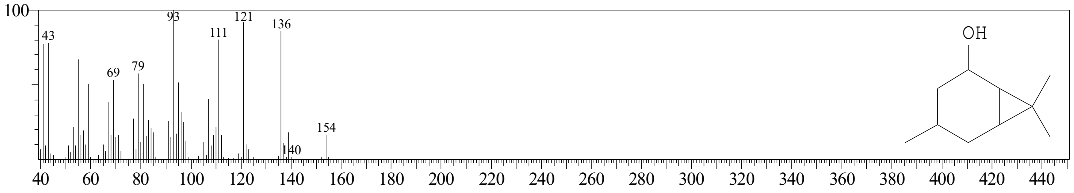

Hit#:4 Entry:25934 Library:NIST11.lib
SI:71 Formula:C10H18O2 CAS:56614-57-4 MolWeight:170 RetIndex:1326
CompName:Bicyclo[2.2.1]heptane-2,3-diol, 1,7,7-trimethyl-, (exo,exo)- $$ 1,7,7-Trimethylbicyclo[2.2.1]heptane-2,3-diol # $$

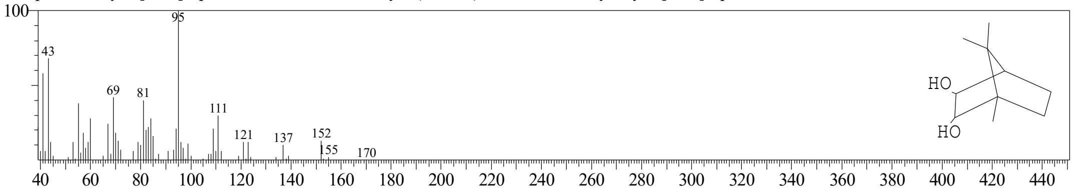

Hit#:5 Entry:6538 Library:NIST11.lib
SI:71 Formula:C8H14O CAS:0-00-0 MolWeight:126 RetIndex:1024
CompName:4,4-Dimethyl-cyclohex-2-en-1-ol $$ 4,4-Dimethyl-2-cyclohexen-1-ol # $$

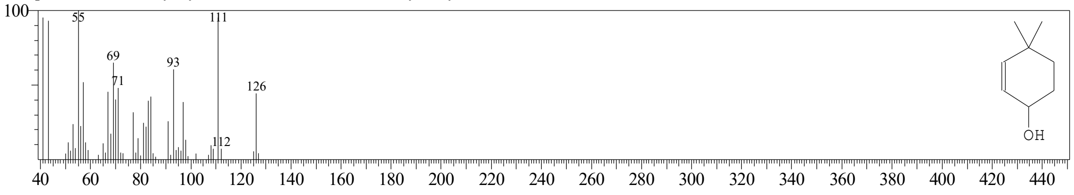

<< Target >>
Line#:21 R.Time:14.975(Scan#:1138) MassPeaks:126
RawMode:Single 14.975(1138) BasePeak:180.00(65004)
BG Mode:None Group 1 - Event 1

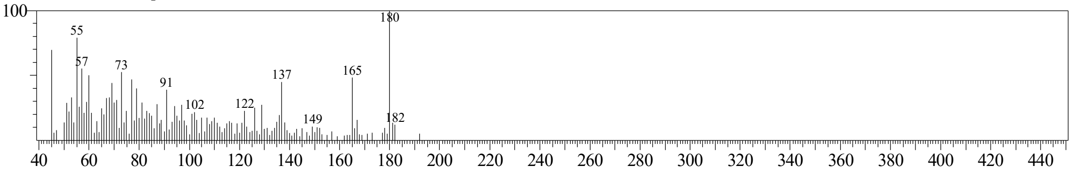

Hit#:1 Entry:81228 Library:NIST11.lib
SI:68 Formula:C16H28O2 CAS:459-67-6 MolWeight:252 RetIndex:1993
CompName:11-(2-Cyclopenten-1-yl)undecanoic acid, (+)- $$ Hydnocarpic acid $$ 2-Cyclopentene-1-undecanoic acid $$ Isolated from chaulmoogra oil $$ 11-(2

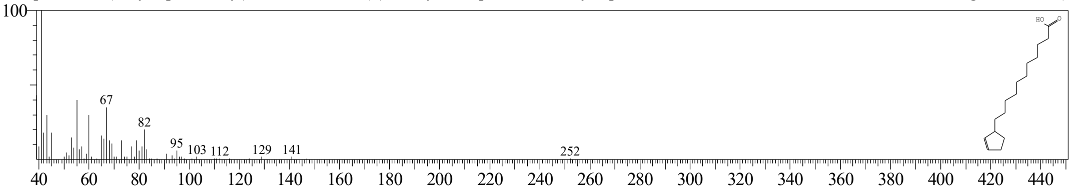

Hit#:2 Entry:82667 Library:NIST11.lib
SI:62 Formula:C16H30O2 CAS:6053-49-2 MolWeight:254 RetIndex:2011
CompName:Cyclopentaneundecanoic acid $$ 11-Cyclopentylundecanoic acid # $$

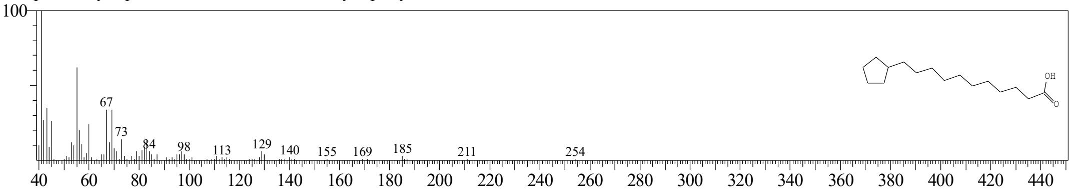

Hit#:3 Entry:51953 Library:NIST11.lib
SI:61 Formula:C11H16O4 CAS:117132-08-8 MolWeight:212 RetIndex:1610
CompName:9,9-Dimethoxybicyclo[3.3.1]nona-2,4-dione $$ 9,9-Dimethoxybicyclo[3.3.1]nonane-2,4-dione # $$

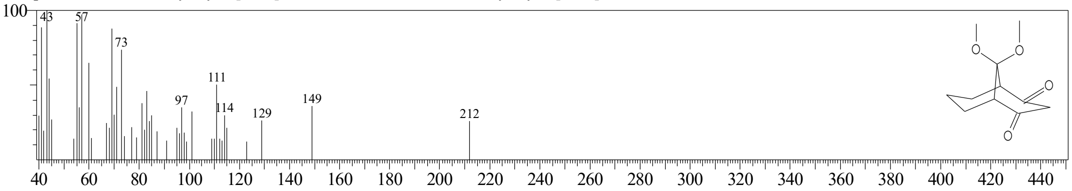

Hit#:4 Entry:90501 Library:NIST11.lib
SI:60 Formula:C18H32O CAS:56630-94-5 MolWeight:264 RetIndex:2077
CompName:6,9,12-Octadecatrien-1-ol $$ (6E,9E,12E)-6,9,12-Octadecatrien-1-ol # $$

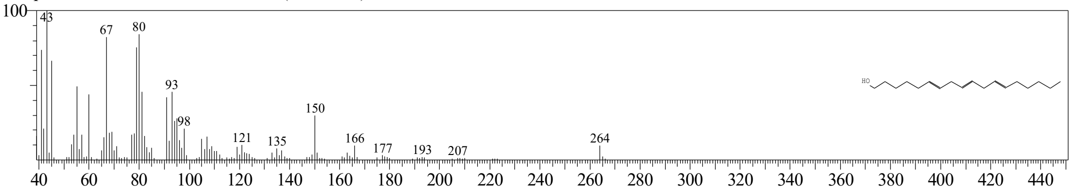

Hit#:5 Entry:14742 Library:NIST11s.lib
SI:60 Formula:C11H18O2 CAS:2777-65-3 MolWeight:182 RetIndex:1469
CompName:Undec-10-ynoic acid $$ 10-Undecynoic acid $$ Undec-1-yn-11-oic acid $$ Hendecynoic acid $$

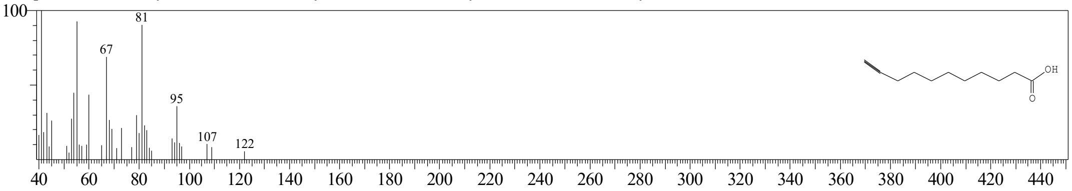

<< Target >>
Line#:22 R.Time:15.058(Scan#:1148) MassPeaks:126
RawMode:Single 15.058(1148) BasePeak:126.95(363272)
BG Mode:None Group 1 - Event 1

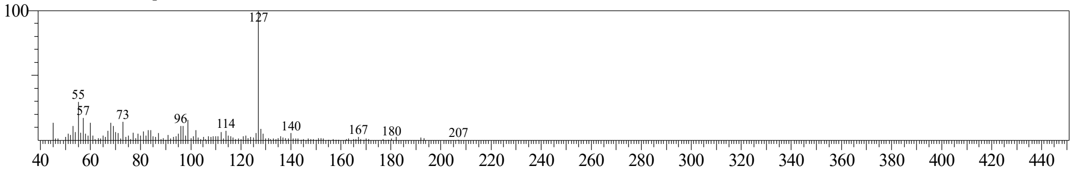

Hit#:1 Entry:6770 Library:NIST11.lib
SI:75 Formula:C4H5N3O2 CAS:0-00-0 MolWeight:127 RetIndex:1241
CompName:4-Hydroxy-2-hydroxyaminopyrimidine $$ 2-(Hydroxyamino)-4-pyrimidinol # $$

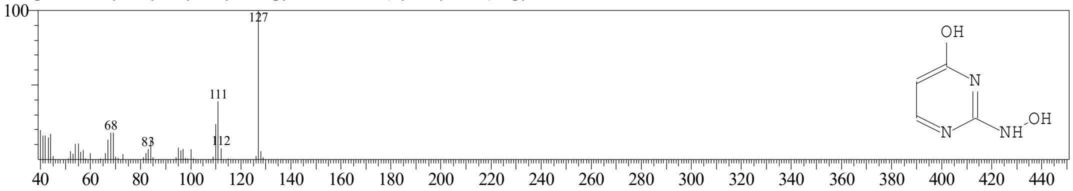

Hit#:2 Entry:28087 Library:NIST11s.lib
SI:74 Formula:C22H44O2 CAS:55193-79-8 MolWeight:340 RetIndex:2311
CompName:Octanoic acid, 1-methyltridecyl ester $$ Octanoic acid, 2-tetradecyl ester $$ 1-Methyltridecyl octanoate # $$

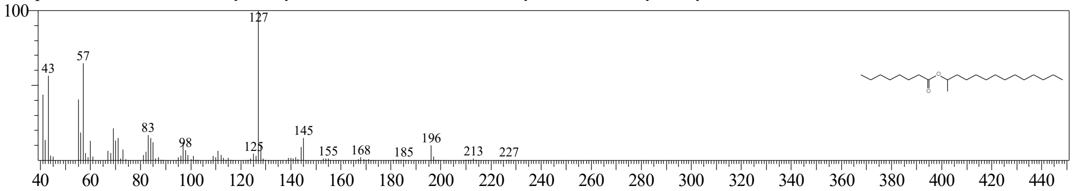

Hit#:3 Entry:18522 Library:NIST11.lib
SI:74 Formula:C9H16O2 CAS:38401-84-2 MolWeight:156 RetIndex:1132
CompName:1,6-Dioxaspiro[4.4]nonane, 2-ethyl- $$ 2-Ethyl-1,6-dioxaspiro[4.4]nonane # $$

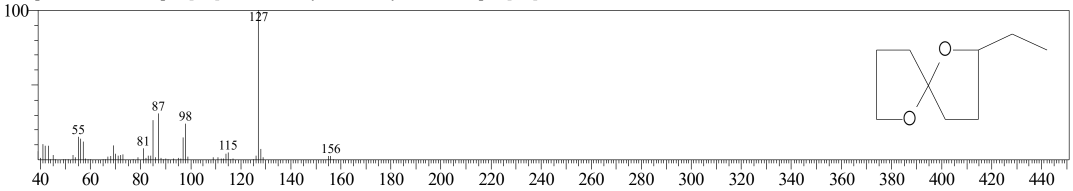

Hit#:4 Entry:52130 Library:NIST11.lib
SI:73 Formula:C12H20O3 CAS:35972-92-0 MolWeight:212 RetIndex:1510
CompName:Spiro[bicyclo[2.2.1]heptane-2,2'-[1,3]dioxolane]-3-ol, 4,7,7-trimethyl-

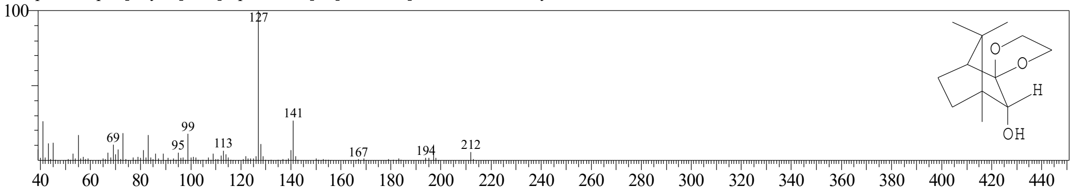

Hit#:5 Entry:158437 Library:NIST11.lib
SI:73 Formula:C23H46O2 CAS:0-00-0 MolWeight:354 RetIndex:2411
CompName:Octanoic acid, 4-pentadecyl ester $$ 1-Propyldodecyl octanoate # $$

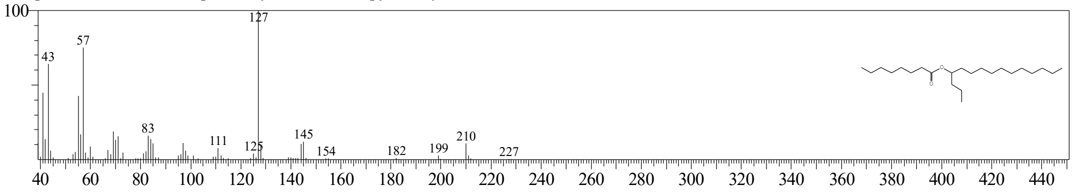

<< Target >>
Line#:23 R.Time:15.142(Scan#:1158) MassPeaks:125
RawMode:Single 15.142(1158) BasePeak:55.00(221737)
BG Mode:None Group 1 - Event 1

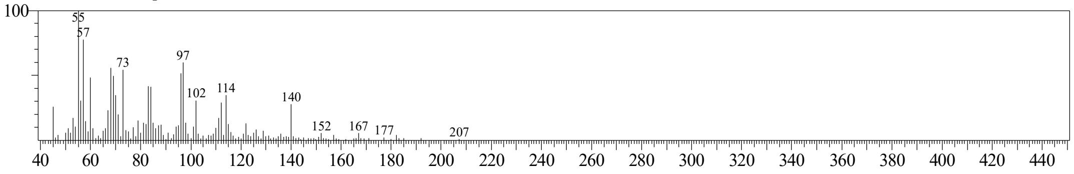

Hit#:1 Entry:19273 Library:NIST11.lib
SI:80 Formula:C8H14O3 CAS:15458-61-4 MolWeight:158 RetIndex:1347
CompName:9-Oxabicyclo[3.3.1]nonane-2,6-diol

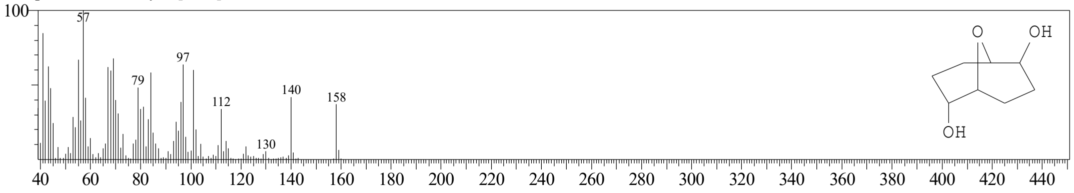

Hit#:2 Entry:51953 Library:NIST11.lib
SI:78 Formula:C11H16O4 CAS:117132-08-8 MolWeight:212 RetIndex:1610
CompName:9,9-Dimethoxybicyclo[3.3.1]nona-2,4-dione $$ 9,9-Dimethoxybicyclo[3.3.1]nonane-2,4-dione # $$

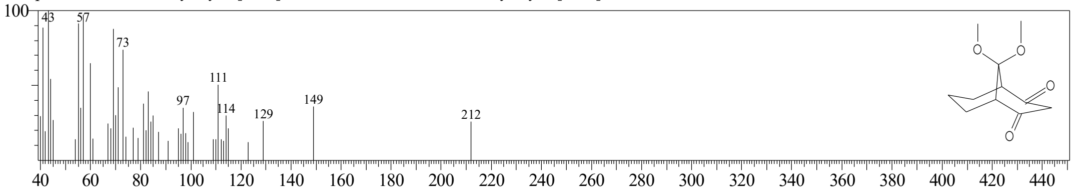

Hit#:3 Entry:33865 Library:NIST11.lib
SI:77 Formula:C11H20O2 CAS:15790-94-0 MolWeight:184 RetIndex:1479
CompName:trans-2-undecenoic acid $$ (2E)-2-Undecenoic acid # $$

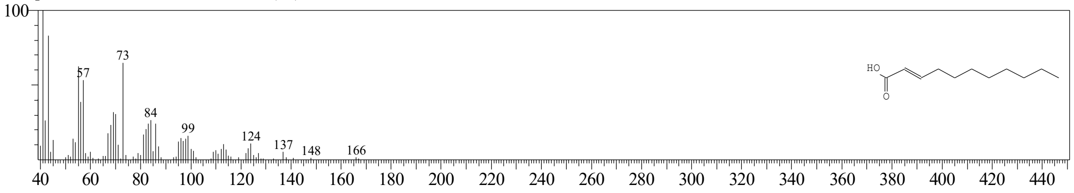

Hit#:4 Entry:33864 Library:NIST11.lib
SI:77 Formula:C11H20O2 CAS:4189-02-0 MolWeight:184 RetIndex:1479
CompName:2-Undecenoic acid $$ Undec-2-enoic acid $$

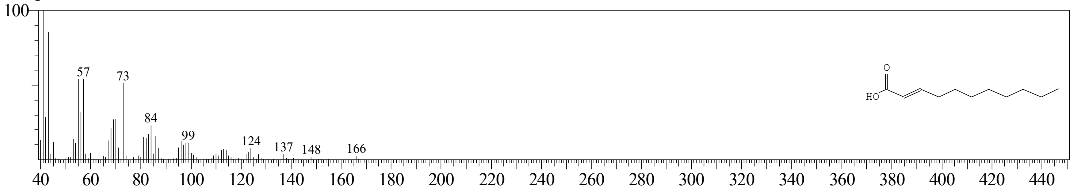

Hit#:5 Entry:42675 Library:NIST11.lib
SI:76 Formula:C12H22O2 CAS:4412-16-2 MolWeight:198 RetIndex:1578
CompName:2-Dodecenoic acid $$ Dodec-2-enoic acid $$

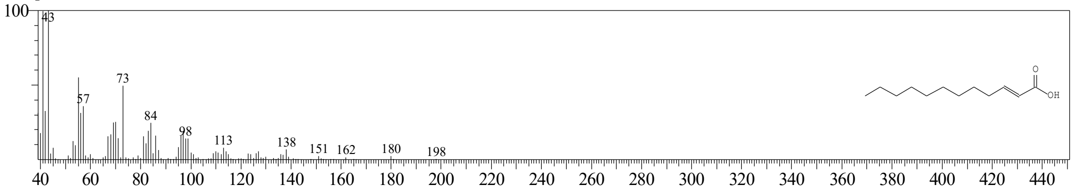

<< Target >>
Line#:24 R.Time:15.625(Scan#:1216) MassPeaks:135
RawMode:Single 15.625(1216) BasePeak:91.00(68652)
BG Mode:None Group 1 - Event 1

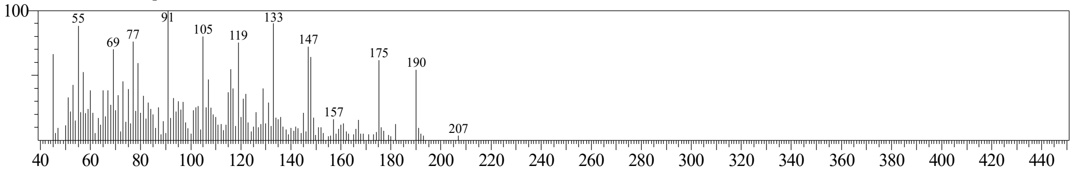

Hit#:1 Entry:37581 Library:NIST11.lib
SI:68 Formula:C13H18O CAS:38818-55-2 MolWeight:190 RetIndex:1454
CompName:Megastigmatrienone $$ 4-[(1E)-1,3-Butadienyl]-3,5,5-trimethyl-2-cyclohexen-1-one # $$

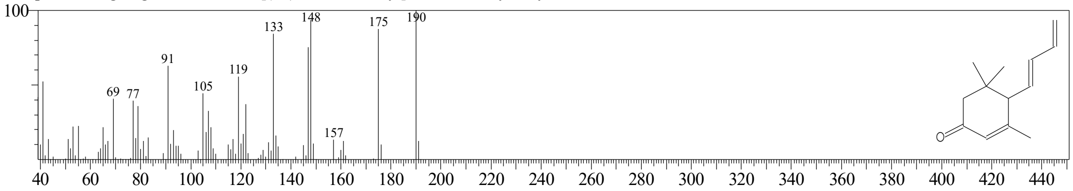

Hit#:2 Entry:21400 Library:NIST11.lib
SI:65 Formula:C12H18 CAS:0-00-0 MolWeight:162 RetIndex:1052
CompName:2-(3,3-Dimethyl-but-1-ynyl)-1,1-dimethyl-3-methylene-cyclopropane $$ 2-(3,3-Dimethyl-1-butynyl)-1,1-dimethyl-3-methylenecyclopropane # $$

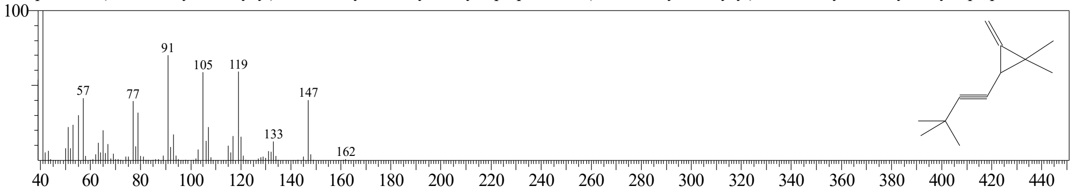

Hit#:3 Entry:21399 Library:NIST11.lib
SI:64 Formula:C12H18 CAS:0-00-0 MolWeight:162 RetIndex:1052
CompName:1-(3,3-Dimethyl-but-1-ynyl)-1,2-dimethyl-3-methylene-cyclopropane $$ 1-(3,3-Dimethyl-1-butynyl)-1,2-dimethyl-3-methylenecyclopropane # $$

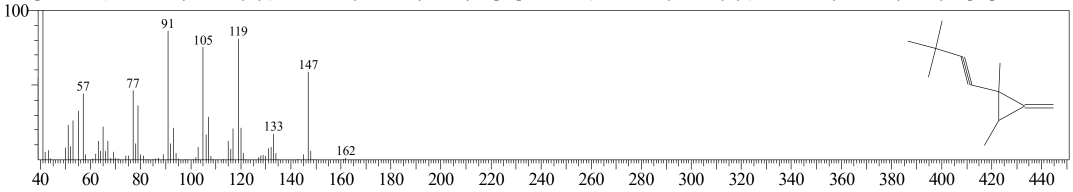

Hit#:4 Entry:70739 Library:NIST11.lib
SI:64 Formula:C15H26O2 CAS:88588-48-1 MolWeight:238 RetIndex:1786
CompName:Cedran-diol, 8S,13- $$ Cedrane-8,13-diol # $$

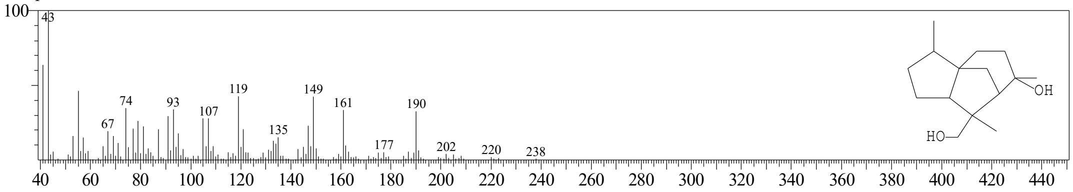

Hit#:5 Entry:131287 Library:NIST11.lib
SI:64 Formula:C21H32O2 CAS:10178-35-5 MolWeight:316 RetIndex:2176
CompName:1-Naphthalenecarboxylic acid, decahydro-1,4a-dimethyl-6-methylene-5-(3-methyl-2,4-pentadienyl)-, methyl ester, [1S-[1.alpha.,4a.alpha.,5.alpha.(Z

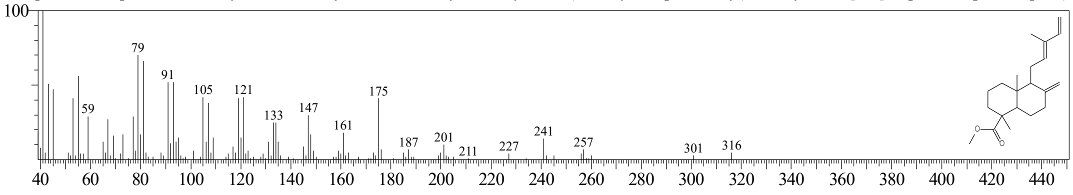

<< Target >>
Line#:25 R.Time:15.767(Scan#:1233) MassPeaks:142
RawMode:Single 15.767(1233) BasePeak:108.00(567570)
BG Mode:None Group 1 - Event 1

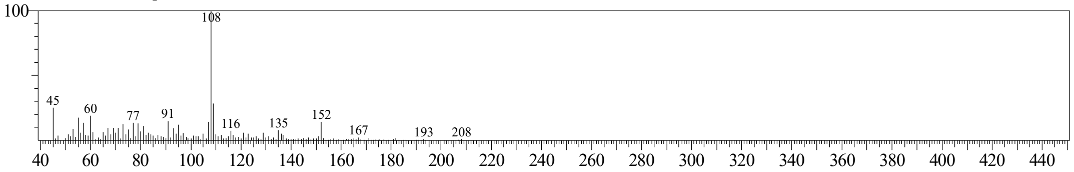

Hit#:1 Entry:49452 Library:NIST11.lib
SI:78 Formula:C13H20O2 CAS:52210-15-8 MolWeight:208 RetIndex:1627
CompName:2-Cyclohexen-1-one, 4-(3-hydroxy-1-butenyl)-3,5,5-trimethyl-, [R-[R*,R*-(E)]]- $$ 2-Cyclohexen-1-one, 4-[(1E,3R)-3-hydroxy-1-buten-1-yl]-3,5,

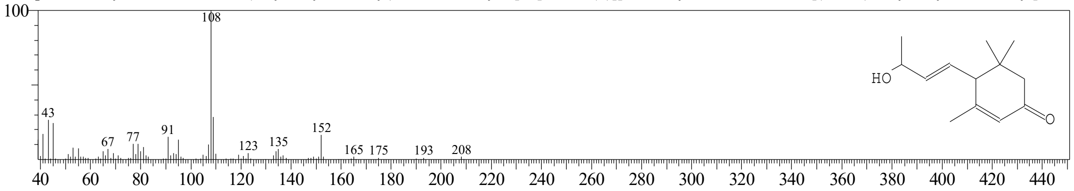

Hit#:2 Entry:49451 Library:NIST11.lib
SI:74 Formula:C13H20O2 CAS:34318-21-3 MolWeight:208 RetIndex:1627
CompName:2-Cyclohexen-1-one, 4-(3-hydroxy-1-butenyl)-3,5,5-trimethyl- $$ 3-Oxo-.alpha.-ionol $$ 4-(3-Hydroxy-1-butenyl)-3,5,5-trimethyl-2-cyclohexen-1-

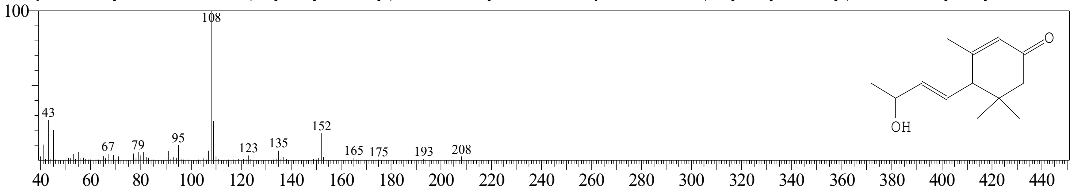

Hit#:3 Entry:9560 Library:NIST11s.lib
SI:73 Formula:C10H16O CAS:4501-58-0 MolWeight:152 RetIndex:1155
CompName:.alpha.-Campholenal $$ (R)-.alpha.-Campholene aldehyde $$ 3-Cyclopentene-1-acetaldehyde, 2,2,3-trimethyl- $$ .alpha.-Compholene aldehyde $$

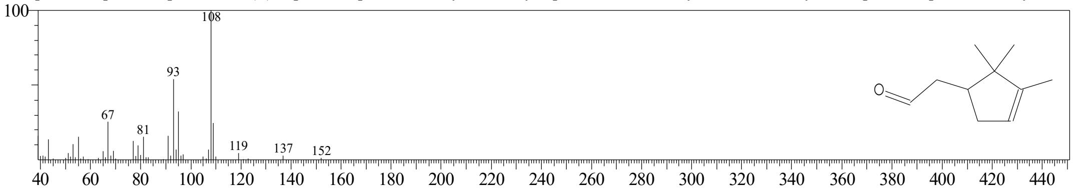

Hit#:4 Entry:15539 Library:NIST11.lib
SI:71 Formula:C11H18 CAS:115948-98-6 MolWeight:150 RetIndex:1027
CompName:2,2-Dimethyl-3-vinyl-bicyclo[2.2.1]heptane

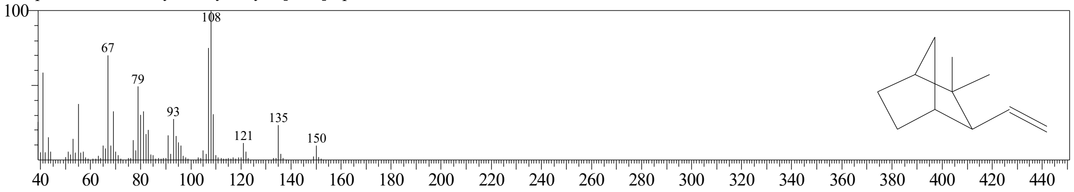

Hit#:5 Entry:16472 Library:NIST11.lib
SI:71 Formula:C10H16O CAS:4501-58-0 MolWeight:152 RetIndex:1155
CompName:.alpha.-Campholenal $$ (R)-.alpha.-Campholene aldehyde $$ 3-Cyclopentene-1-acetaldehyde, 2,2,3-trimethyl- $$ .alpha.-Compholene aldehyde $$

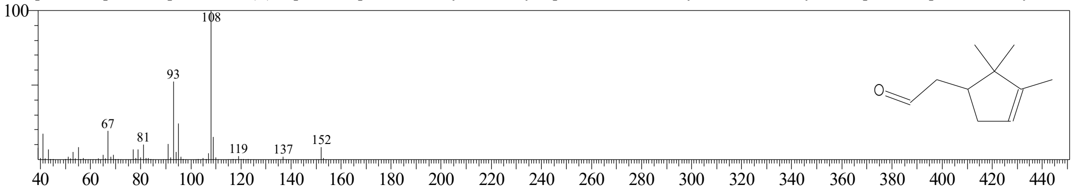

<< Target >>
Line#:26 R.Time:16.275(Scan#:1294) MassPeaks:147
RawMode:Single 16.275(1294) BasePeak:60.00(699999)
BG Mode:None Group 1 - Event 1

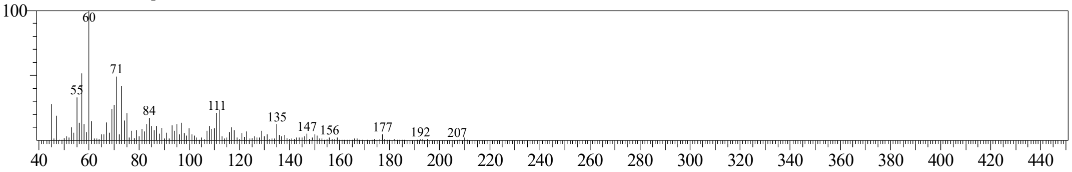

Hit#:1 Entry:14323 Library:NIST11.lib
SI:75 Formula:C6H12O4 CAS:53585-08-3 MolWeight:148 RetIndex:1472
CompName:1,2,3,5-Cyclohexanetetrol, (1.alpha.,2.beta.,3.alpha.,5.beta.)- $$ 1,2,3,5-Cyclohexanetetrol # $$

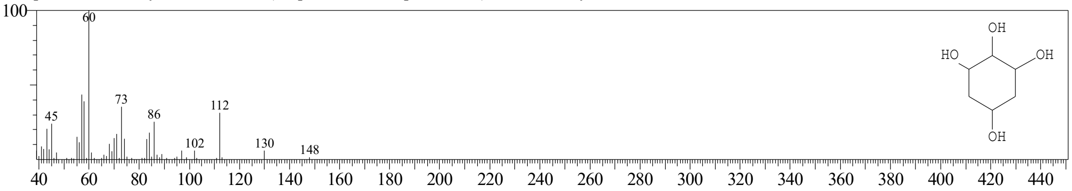

Hit#:2 Entry:27150 Library:NIST11.lib
SI:74 Formula:C10H20S CAS:42779-08-8 MolWeight:172 RetIndex:1256
CompName:Octane, 1-(ethenylthio)- $$ 1-(Octylsulfanyl)ethylene # $$

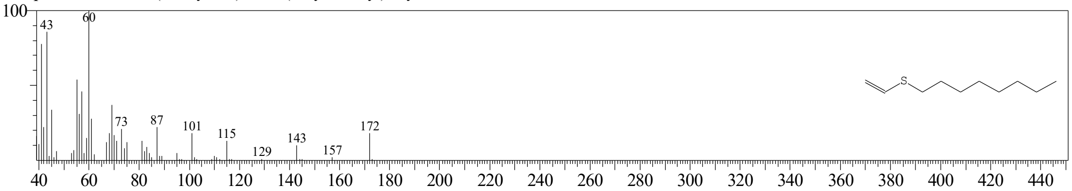

Hit#:3 Entry:13090 Library:NIST11s.lib
SI:73 Formula:C10H20O2 CAS:334-48-5 MolWeight:172 RetIndex:1372
CompName:n-Decanoic acid $$ Decanoic acid $$ n-Capric acid $$ n-Decoic acid $$ n-Decylic acid $$ Capric acid $$ Caprinic acid $$ Caprynic acid $$ Decoic

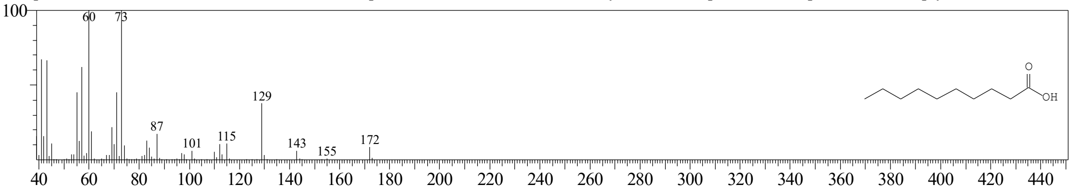

Hit#:4 Entry:100625 Library:NIST11.lib
SI:73 Formula:C10H19AgO2 CAS:13126-67-5 MolWeight:278 RetIndex:0
CompName:Decanoic acid, silver(1+) salt $$ Silver decanoate $$

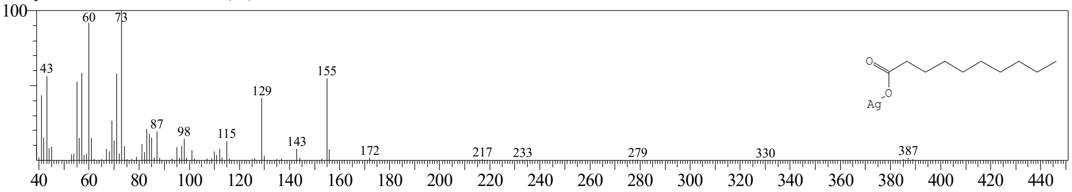

Hit#:5 Entry:13091 Library:NIST11s.lib
SI:73 Formula:C10H20O2 CAS:334-48-5 MolWeight:172 RetIndex:1372
CompName:n-Decanoic acid $$ Decanoic acid $$ n-Capric acid $$ n-Decoic acid $$ n-Decylic acid $$ Capric acid $$ Caprinic acid $$ Caprynic acid $$ Decoic

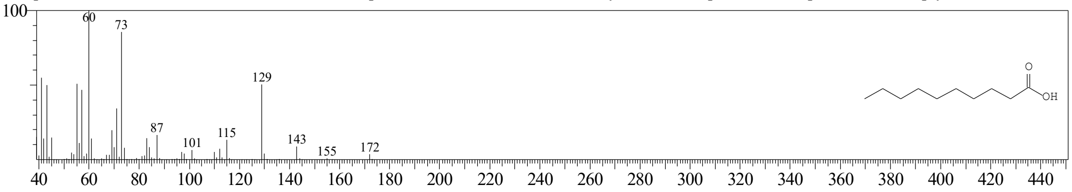

<< Target >>
Line#:27 R.Time:16.275(Scan#:1294) MassPeaks:147
RawMode:Single 16.275(1294) BasePeak:60.00(699999)
BG Mode:None Group 1 - Event 1

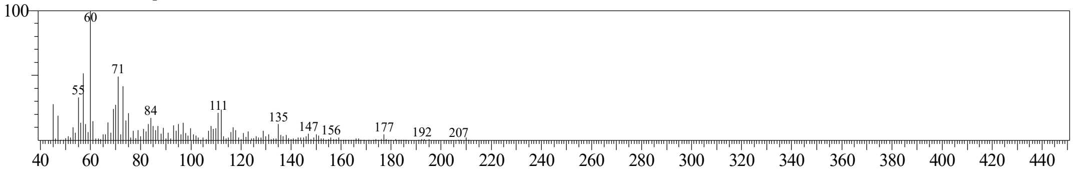

Hit#:1 Entry:14323 Library:NIST11.lib
SI:75 Formula:C6H12O4 CAS:53585-08-3 MolWeight:148 RetIndex:1472
CompName:1,2,3,5-Cyclohexanetetrol, (1.alpha.,2.beta.,3.alpha.,5.beta.)- $$ 1,2,3,5-Cyclohexanetetrol # $$

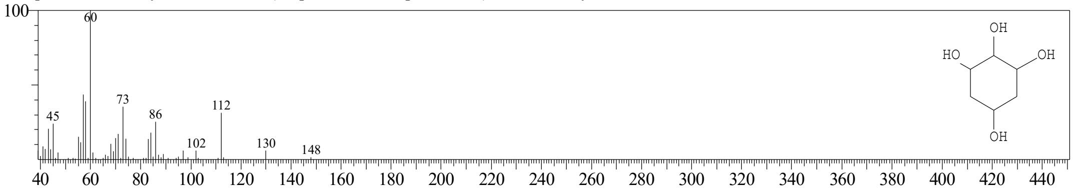

Hit#:2 Entry:27150 Library:NIST11.lib
SI:74 Formula:C10H20S CAS:42779-08-8 MolWeight:172 RetIndex:1256
CompName:Octane, 1-(ethenylthio)- $$ 1-(Octylsulfanyl)ethylene # $$

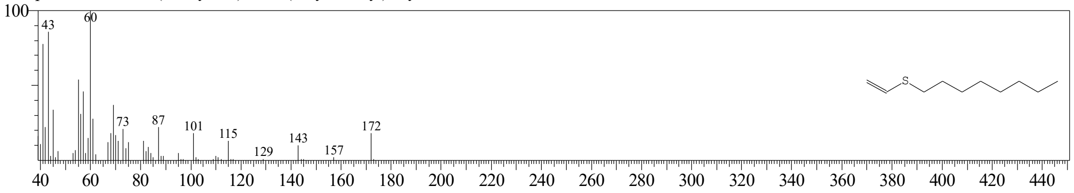

Hit#:3 Entry:13090 Library:NIST11s.lib
SI:73 Formula:C10H20O2 CAS:334-48-5 MolWeight:172 RetIndex:1372
CompName:n-Decanoic acid $$ Decanoic acid $$ n-Capric acid $$ n-Decoic acid $$ n-Decylic acid $$ Capric acid $$ Caprinic acid $$ Caprynic acid $$ Decoic

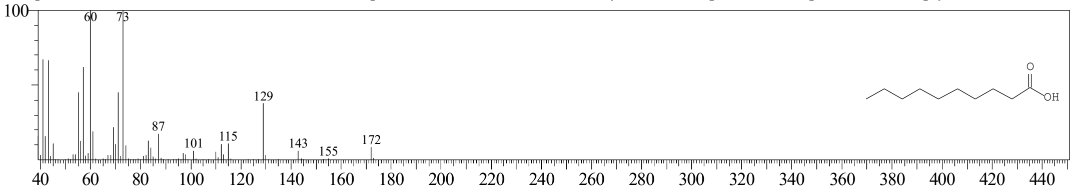

Hit#:4 Entry:100625 Library:NIST11.lib
SI:73 Formula:C10H19AgO2 CAS:13126-67-5 MolWeight:278 RetIndex:0
CompName:Decanoic acid, silver(1+) salt $$ Silver decanoate $$

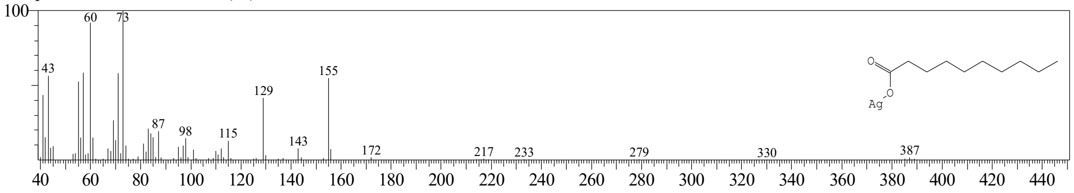

Hit#:5 Entry:13091 Library:NIST11s.lib
SI:73 Formula:C10H20O2 CAS:334-48-5 MolWeight:172 RetIndex:1372
CompName:n-Decanoic acid $$ Decanoic acid $$ n-Capric acid $$ n-Decoic acid $$ n-Decylic acid $$ Capric acid $$ Caprinic acid $$ Caprynic acid $$ Decoic

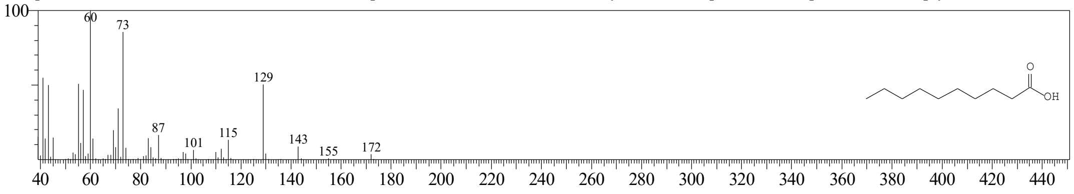

<< Target >>
Line#:28 R.Time:16.592(Scan#:1332) MassPeaks:148
RawMode:Single 16.592(1332) BasePeak:73.00(282847)
BG Mode:None Group 1 - Event 1

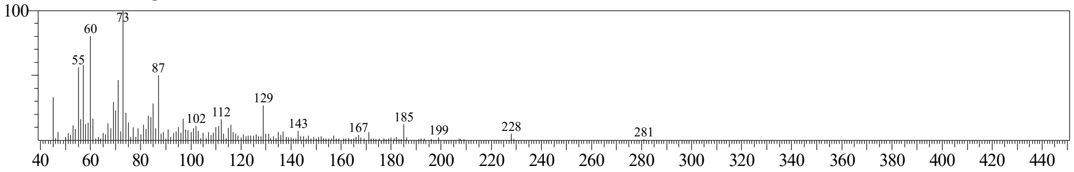

Hit#:1 Entry:53599 Library:NIST11.lib
SI:83 Formula:C13H26O2 CAS:638-53-9 MolWeight:214 RetIndex:1670
CompName:Tridecanoic acid $$ n-Tridecanoic acid $$ n-Tridecoic acid $$ Tridecylic acid $$

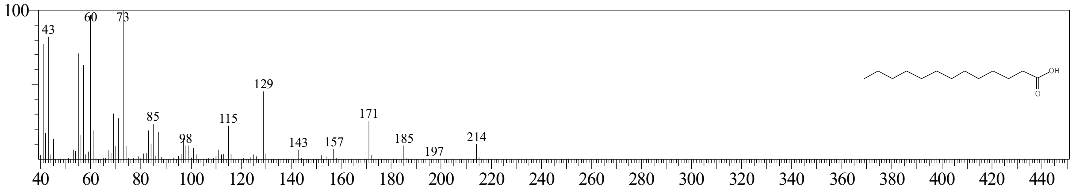

Hit#:2 Entry:100625 Library:NIST11.lib
SI:82 Formula:C10H19AgO2 CAS:13126-67-5 MolWeight:278 RetIndex:0
CompName:Decanoic acid, silver(1+) salt $$ Silver decanoate $$

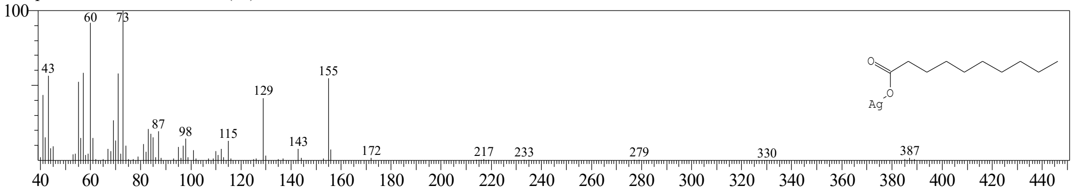

Hit#:3 Entry:73851 Library:NIST11.lib
SI:82 Formula:C15H30O2 CAS:1002-84-2 MolWeight:242 RetIndex:1869
CompName:Pentadecanoic acid $$ Pentadecylic acid $$ n-Pentadecanoic acid $$ n-Pentadecylic acid $$

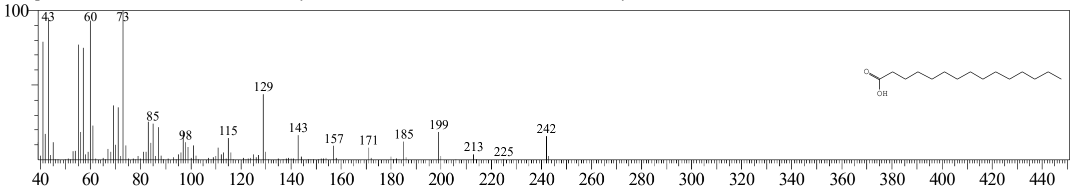

Hit#:4 Entry:63810 Library:NIST11.lib
SI:82 Formula:C14H28O2 CAS:544-63-8 MolWeight:228 RetIndex:1769
CompName:Tetradecanoic acid $$ Myristic acid $$ n-Tetradecanoic acid $$ n-Tetradecoic acid $$ Neo-Fat 14 $$ Univol U 316S $$ 1-Tridecanecarboxylic acid $

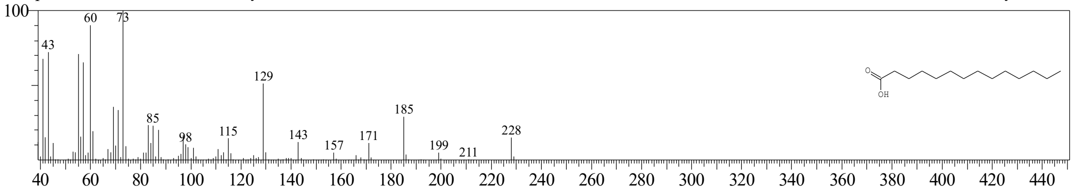

Hit#:5 Entry:19408 Library:NIST11s.lib
SI:82 Formula:C13H26O2 CAS:638-53-9 MolWeight:214 RetIndex:1670
CompName:Tridecanoic acid $$ n-Tridecanoic acid $$ n-Tridecoic acid $$ Tridecylic acid $$

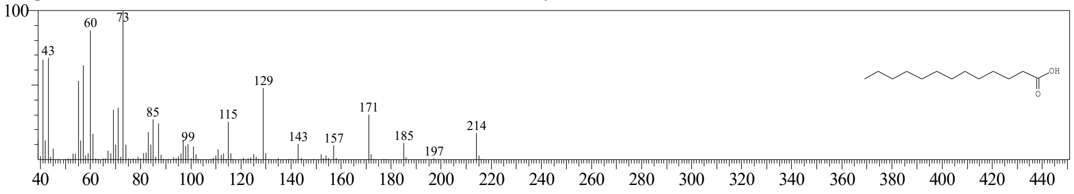

<< Target >>
Line#:29 R.Time:16.767(Scan#:1353) MassPeaks:154
RawMode:Single 16.767(1353) BasePeak:73.00(352554)
BG Mode:None Group 1 - Event 1

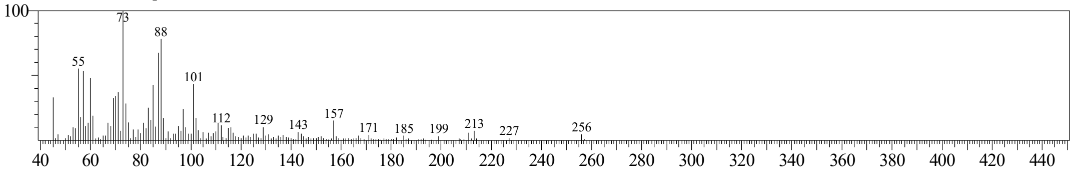

Hit#:1 Entry:17521 Library:NIST11s.lib
SI:77 Formula:C12H24O2 CAS:143-07-7 MolWeight:200 RetIndex:1570
CompName:Dodecanoic acid $$ n-Dodecanoic acid $$ Neo-fat 12 $$ Aliphat no. 4 $$ ABL $$ Dodecylic acid $$ Lauric acid $$ Laurostearic acid $$ Neo-fat 12-

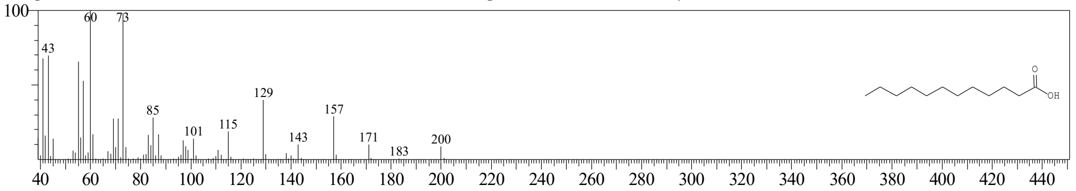

Hit#:2 Entry:23313 Library:NIST11s.lib
SI:76 Formula:C16H32O2 CAS:57-10-3 MolWeight:256 RetIndex:1968
CompName:n-Hexadecanoic acid $$ Hexadecanoic acid $$ n-Hexadecoic acid $$ Palmitic acid $$ Pentadecanecarboxylic acid $$ 1-Pentadecanecarboxylic acid

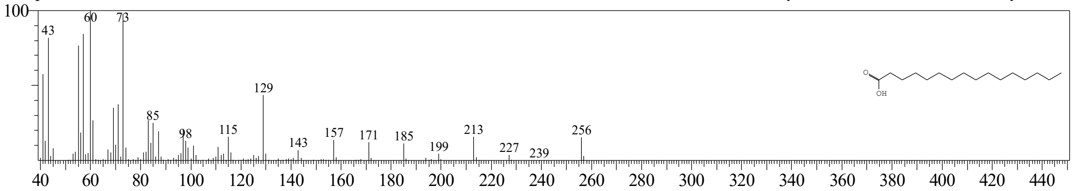

Hit#:3 Entry:166830 Library:NIST11.lib
SI:76 Formula:C24H48O2 CAS:5908-87-2 MolWeight:368 RetIndex:2574
CompName:Docosanoic acid, ethyl ester $$ Ethyl docosanoate # $$

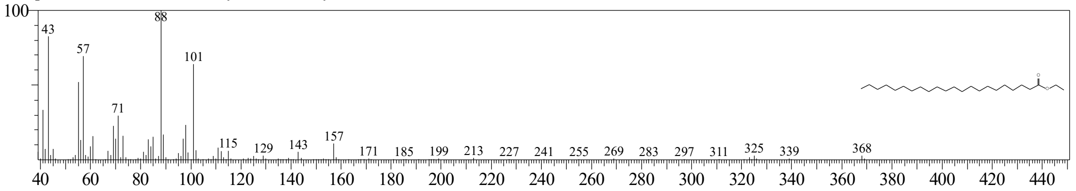

Hit#:4 Entry:44009 Library:NIST11.lib
SI:76 Formula:C12H24O2 CAS:143-07-7 MolWeight:200 RetIndex:1570
CompName:Dodecanoic acid $$ n-Dodecanoic acid $$ Neo-fat 12 $$ Aliphat no. 4 $$ ABL $$ Dodecylic acid $$ Lauric acid $$ Laurostearic acid $$ Neo-fat 12-

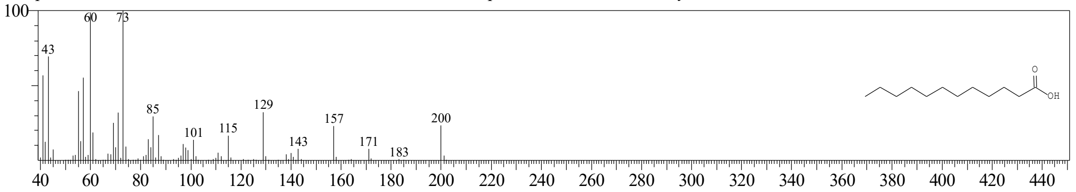

Hit#:5 Entry:25180 Library:NIST11s.lib
SI:76 Formula:C18H36O2 CAS:628-97-7 MolWeight:284 RetIndex:1978
CompName:Hexadecanoic acid, ethyl ester $$ Palmitic acid, ethyl ester $$ Ethyl hexadecanoate $$ Ethyl palmitate $$ Ethyl n-hexadecanoate $$

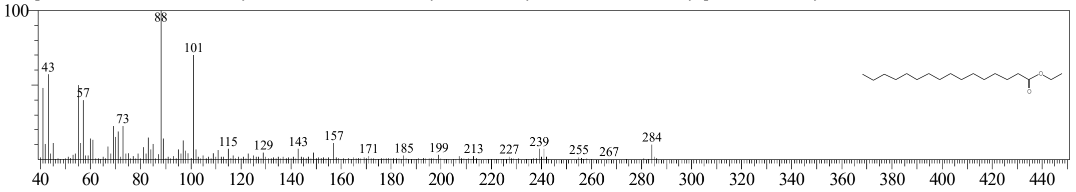

<< Target >>
Line#:30 R.Time:16.908(Scan#:1370) MassPeaks:147
RawMode:Single 16.908(1370) BasePeak:73.00(357241)
BG Mode:None Group 1 - Event 1

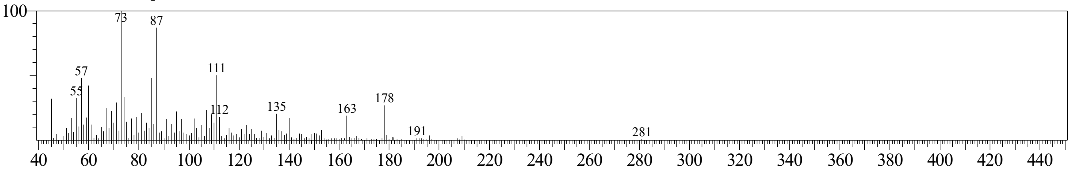

Hit#:1 Entry:135733 Library:NIST11.lib
SI:69 Formula:C21H38O2 CAS:56687-68-4 MolWeight:322 RetIndex:2203
CompName:[1,1'-Bicyclopropyl]-2-octanoic acid, 2'-hexyl-, methyl ester

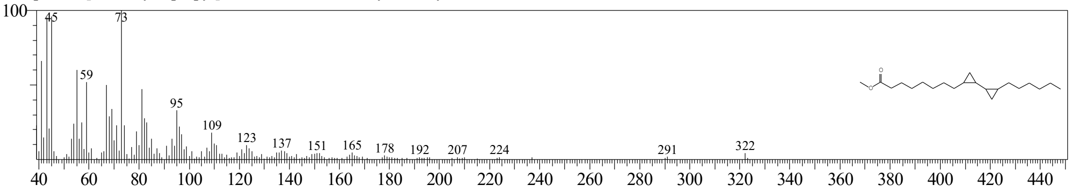

Hit#:2 Entry:212076 Library:NIST11.lib
SI:68 Formula:C36H54O14 CAS:560-53-2 MolWeight:710 RetIndex:5558
CompName:Card-20(22)-enolide, 3-[(2,6-dideoxy-4-O-.beta.-D-glucopyranosyl-3-O-methyl-.beta.-D-ribo-hexopyranosyl)oxy]-5,14-dihydroxy-19-oxo-, (3.beta.

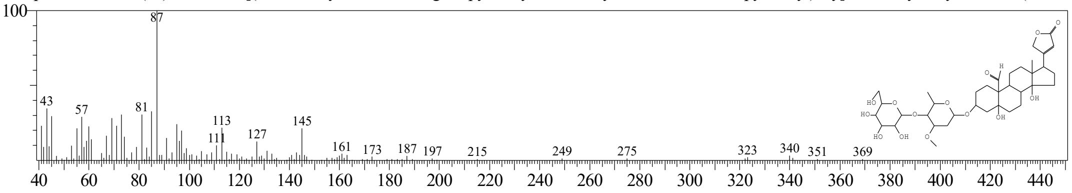

Hit#:3 Entry:39438 Library:NIST11.lib
SI:66 Formula:C7H14O6 CAS:0-00-0 MolWeight:194 RetIndex:1647
CompName:3-O-Methyl-d-glucose $$ 3-O-Methylhexose # $$

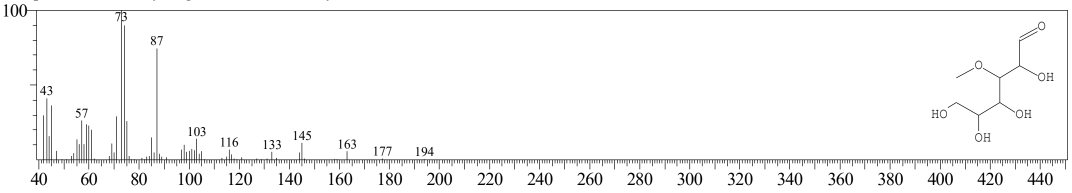

Hit#:4 Entry:26685 Library:NIST11s.lib
SI:66 Formula:C19H34O3 CAS:40596-69-8 MolWeight:310 RetIndex:1933
CompName:Methoprene $$ 2,4-Dodecadienoic acid, 11-methoxy-3,7,11-trimethyl-, 1-methylethyl ester, (E,E)- $$ Altosid $$ Altosid SR 10 $$ Altoside $$ ENT

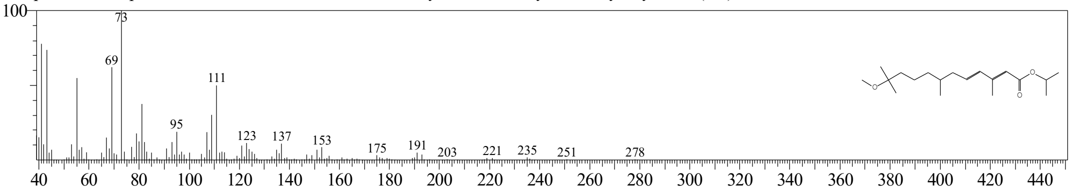

Hit#:5 Entry:205078 Library:NIST11.lib
SI:66 Formula:C18H32O16 CAS:597-12-6 MolWeight:504 RetIndex:4506
CompName:.alpha.-D-Glucopyranoside, O-.alpha.-D-glucopyranosyl-(1.fwdarw.3)-.beta.-D-fructofuranosyl $$ Glucopyranoside, O-.alpha.-D-glucopyranosyl-(1

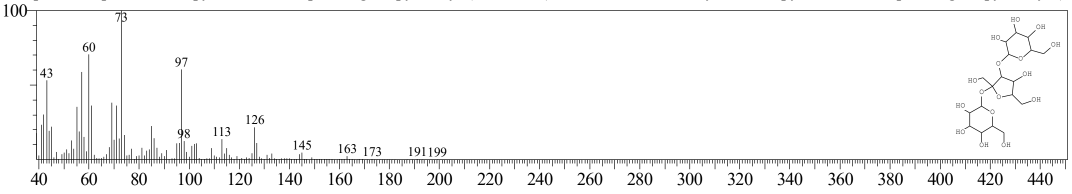

<< Target >>
Line#:31 R.Time:16.983(Scan#:1379) MassPeaks:149
RawMode:Single 16.983(1379) BasePeak:124.00(1320406)
BG Mode:None Group 1 - Event 1

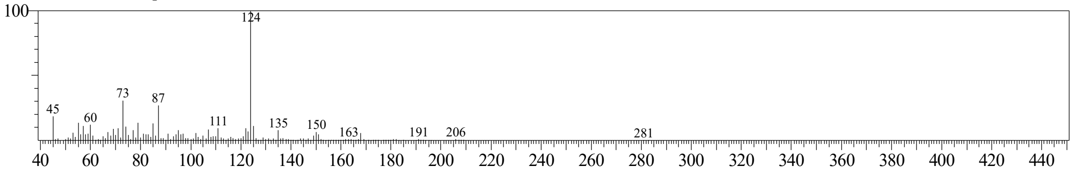

Hit#:1 Entry:107111 Library:NIST11.lib
SI:72 Formula:C13H18O5S CAS:0-00-0 MolWeight:286 RetIndex:2575
CompName:2-O-p-Methylphenyl-1-thio-.beta.-d-glucoside $$ 2-S-(4-Methylphenyl)-2-thiohexopyranose # $$

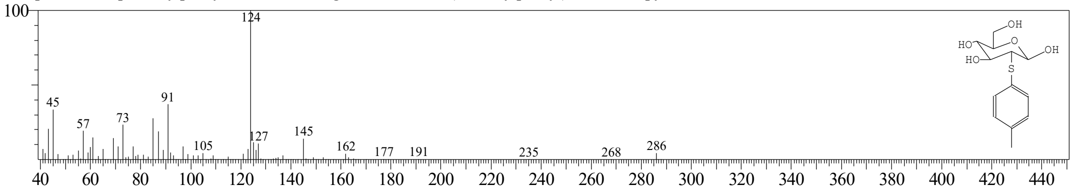

Hit#:2 Entry:107112 Library:NIST11.lib
SI:71 Formula:C13H18O5S CAS:0-00-0 MolWeight:286 RetIndex:2478
CompName:2-Methylphenyl-1-thio-.beta.-d-galactoside $$ 2-O-(4-Methylphenyl)-1-thiohexopyranose # $$

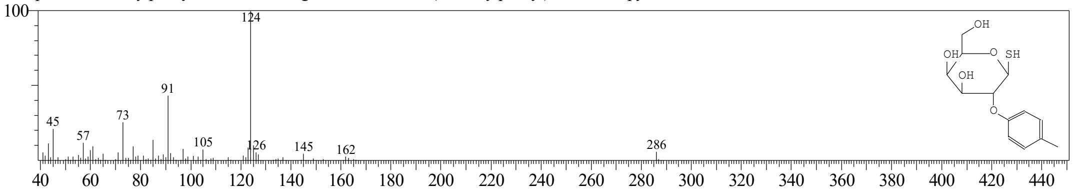

Hit#:3 Entry:107113 Library:NIST11.lib
SI:69 Formula:C13H18O5S CAS:0-00-0 MolWeight:286 RetIndex:2575
CompName:S-4-Methylphenyl-.beta.-D-glucothioside $$ 4-Methylphenyl 1-thiohexopyranoside # $$

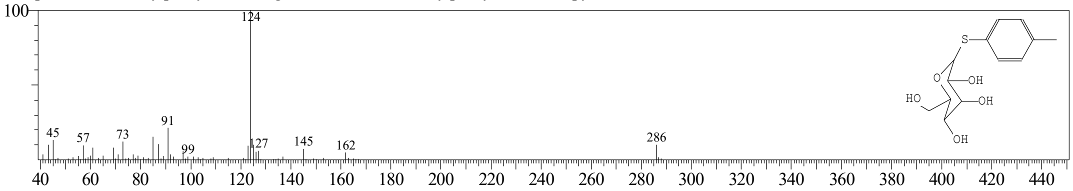

Hit#:4 Entry:72823 Library:NIST11.lib
SI:68 Formula:C13H23NO3 CAS:43224-65-3 MolWeight:241 RetIndex:1665
CompName:1-Acetyl-2,2,6,6-tetramethyl-4-acetyloxypiperidyne $$ 1-Acetyl-2,2,6,6-tetramethyl-4-piperidinyl acetate # $$

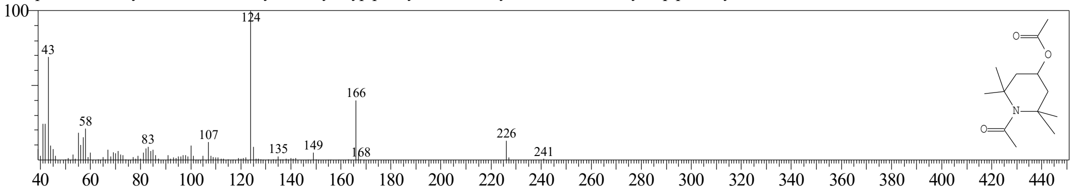

Hit#:5 Entry:33969 Library:NIST11.lib
SI:68 Formula:C11H20O2 CAS:22882-86-6 MolWeight:184 RetIndex:1415
CompName:4-Decenoic acid, 3-methyl-, (E)- $$ (4E)-3-Methyl-4-decenoic acid # $$

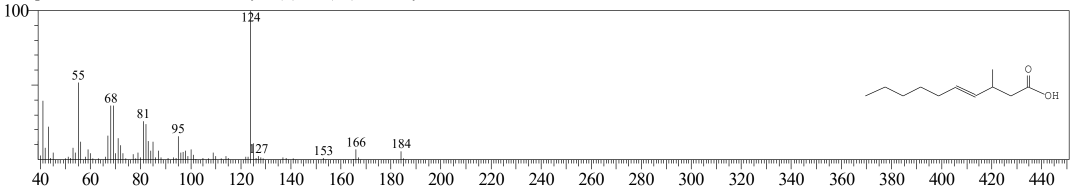

<< Target >>
Line#:32 R.Time:17.100(Scan#:1393) MassPeaks:149
RawMode:Single 17.100(1393) BasePeak:73.00(477374)
BG Mode:None Group 1 - Event 1

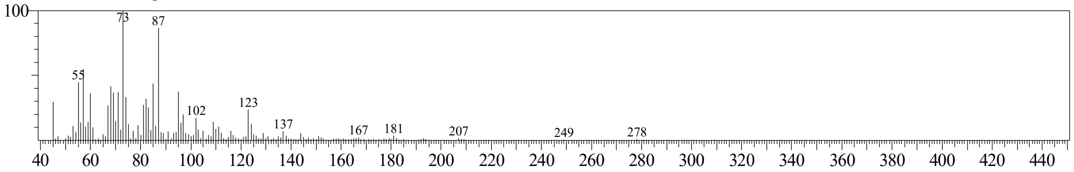

Hit#:1 Entry:135733 Library:NIST11.lib
SI:81 Formula:C21H38O2 CAS:56687-68-4 MolWeight:322 RetIndex:2203
CompName:[1,1'-Bicyclopropyl]-2-octanoic acid, 2'-hexyl-, methyl ester

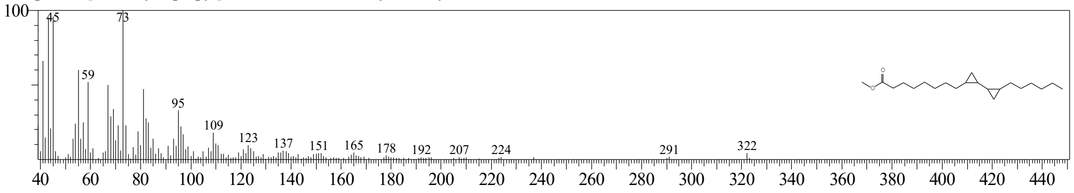

Hit#:2 Entry:33864 Library:NIST11.lib
SI:75 Formula:C11H20O2 CAS:4189-02-0 MolWeight:184 RetIndex:1479
CompName:2-Undecenoic acid $$ Undec-2-enoic acid $$

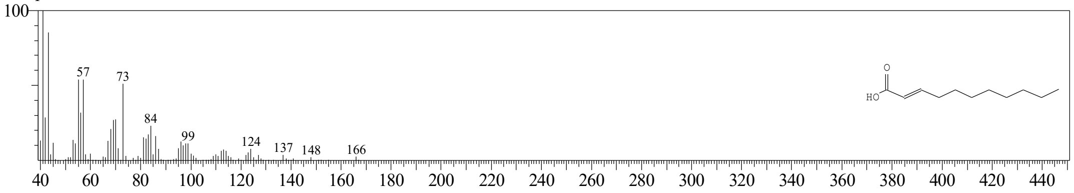

Hit#:3 Entry:93487 Library:NIST11.lib
SI:75 Formula:C17H32O2 CAS:0-00-0 MolWeight:268 RetIndex:1822
CompName:10-Methyl-E-11-tridecen-1-ol propionate $$ (11E)-10-Methyl-11-tridecenyl propionate # $$

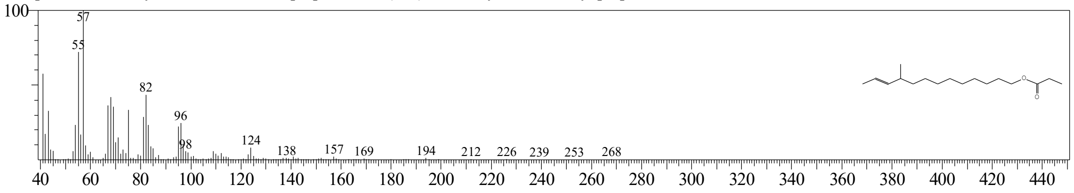

Hit#:4 Entry:33865 Library:NIST11.lib
SI:75 Formula:C11H20O2 CAS:15790-94-0 MolWeight:184 RetIndex:1479
CompName:trans-2-undecenoic acid $$ (2E)-2-Undecenoic acid # $$

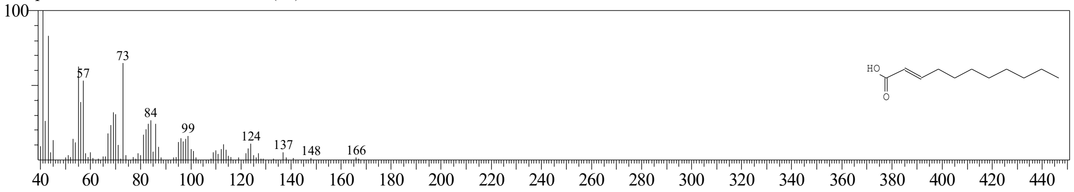

Hit#:5 Entry:25037 Library:NIST11s.lib
SI:74 Formula:C18H34O2 CAS:112-80-1 MolWeight:282 RetIndex:2175
CompName:Oleic Acid $$ 9-Octadecenoic acid (Z)- $$ .DELTA.9-cis-Oleic acid $$ cis-Oleic Acid $$ cis-9-Octadecenoic Acid $$ Emersol 211 $$ Emersol 220 \

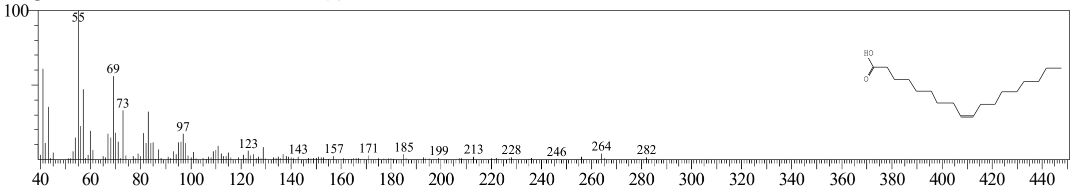

<< Target >>
Line#:33 R.Time:17.333(Scan#:1421) MassPeaks:156
RawMode:Single 17.333(1421) BasePeak:73.00(658908)
BG Mode:None Group 1 - Event 1

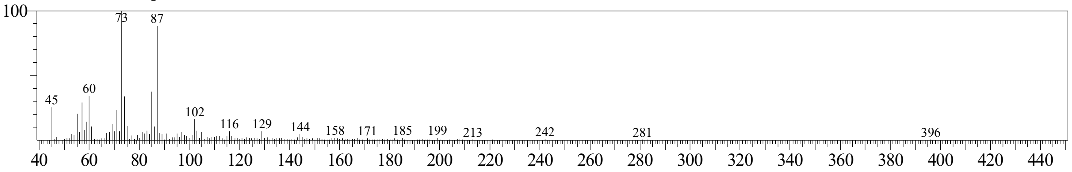

Hit#:1 Entry:39438 Library:NIST11.lib
SI:85 Formula:C7H14O6 CAS:0-00-0 MolWeight:194 RetIndex:1647
CompName:3-O-Methyl-d-glucose $$ 3-O-Methylhexose # $$

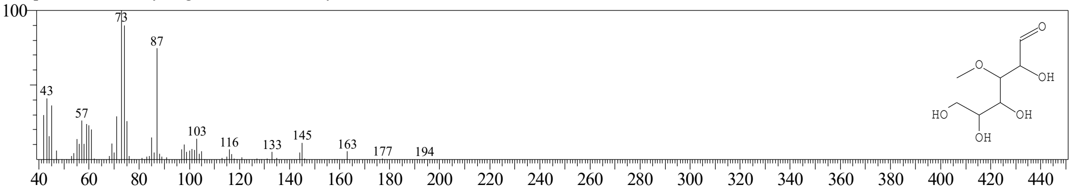

Hit#:2 Entry:39435 Library:NIST11.lib
SI:82 Formula:C7H14O6 CAS:0-00-0 MolWeight:194 RetIndex:1667
CompName:.alpha.-Methyl mannofuranoside $$ Methyl hexofuranoside # $$

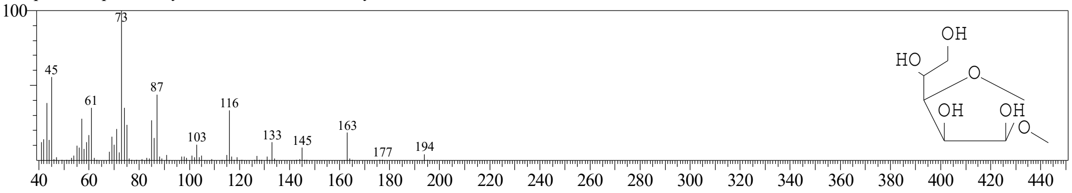

Hit#:3 Entry:14260 Library:NIST11s.lib
SI:80 Formula:C6H12O6 CAS:3458-28-4 MolWeight:180 RetIndex:1698
CompName:d-Mannose $$ Mannose, d- $$ Carubinose $$ d(+)-Mannose $$ Mannose $$ Seminose $$ Hexose # $$

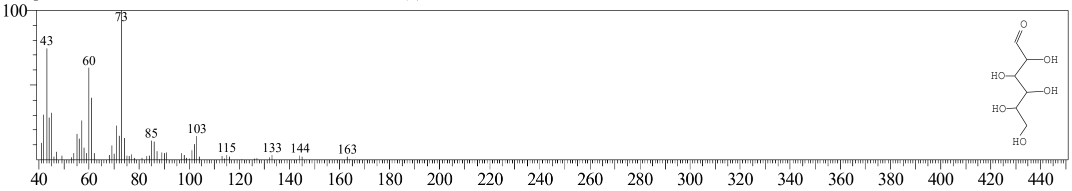

Hit#:4 Entry:39436 Library:NIST11.lib
SI:80 Formula:C7H14O6 CAS:4097-91-0 MolWeight:194 RetIndex:1667
CompName:.alpha.-d-Mannofuranoside, methyl $$ Methyl hexofuranoside # $$

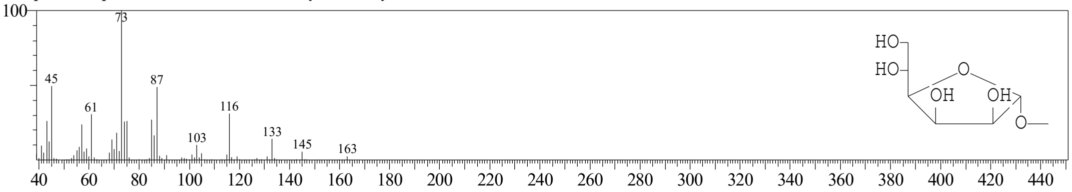

Hit#:5 Entry:142911 Library:NIST11.lib
SI:79 Formula:C17H32O6 CAS:0-00-0 MolWeight:332 RetIndex:2651
CompName:.beta.-D-Mannofuranoside, 1-O-(10-undecenyl)- $$ 10-Undecenyl hexofuranoside # $$

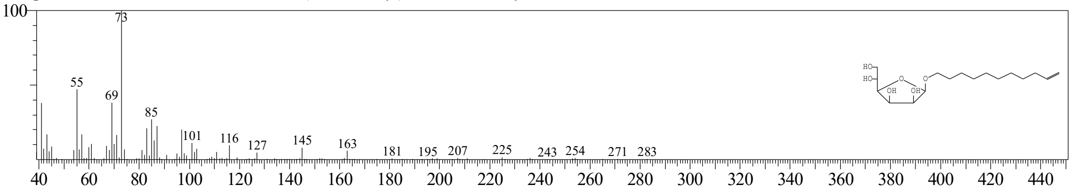

<< Target >>
Line#:34 R.Time:18.125(Scan#:1516) MassPeaks:200
RawMode:Single 18.125(1516) BasePeak:73.00(1779060)
BG Mode:None Group 1 - Event 1

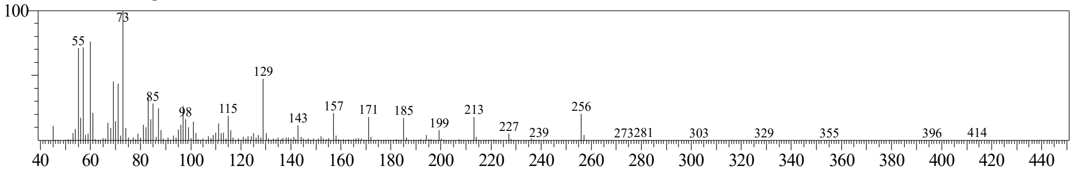

Hit#:1 Entry:23313 Library:NIST11s.lib
SI:93 Formula:C16H32O2 CAS:57-10-3 MolWeight:256 RetIndex:1968
CompName:n-Hexadecanoic acid $$ Hexadecanoic acid $$ n-Hexadecoic acid $$ Palmitic acid $$ Pentadecanecarboxylic acid $$ 1-Pentadecanecarboxylic acid

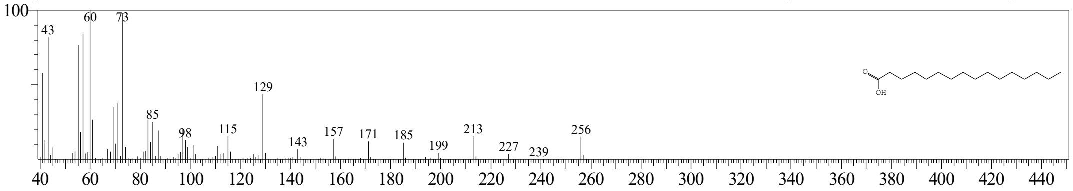

Hit#:2 Entry:211367 Library:NIST11.lib
SI:91 Formula:C38H68O8 CAS:28474-90-0 MolWeight:652 RetIndex:4765
CompName:l-(+)-Ascorbic acid 2,6-dihexadecanoate

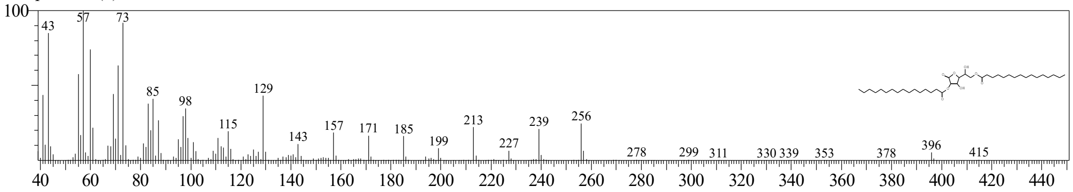

Hit#:3 Entry:22188 Library:NIST11s.lib
SI:90 Formula:C15H30O2 CAS:1002-84-2 MolWeight:242 RetIndex:1869
CompName:Pentadecanoic acid $$ Pentadecylic acid $$ n-Pentadecanoic acid $$ n-Pentadecylic acid $$

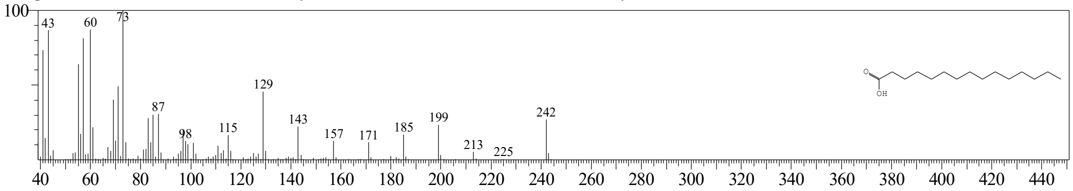

Hit#:4 Entry:23305 Library:NIST11s.lib
SI:90 Formula:C16H32O2 CAS:57-10-3 MolWeight:256 RetIndex:1968
CompName:n-Hexadecanoic acid $$ Hexadecanoic acid $$ n-Hexadecoic acid $$ Palmitic acid $$ Pentadecanecarboxylic acid $$ 1-Pentadecanecarboxylic acid

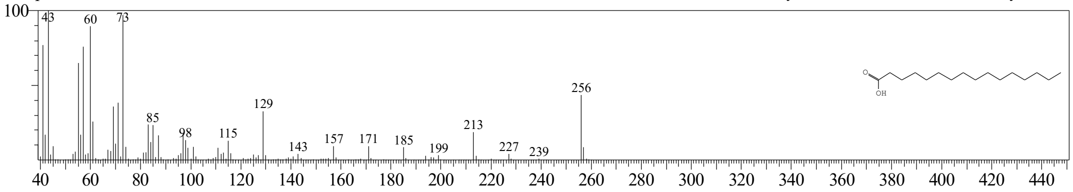

Hit#:5 Entry:73851 Library:NIST11.lib
SI:90 Formula:C15H30O2 CAS:1002-84-2 MolWeight:242 RetIndex:1869
CompName:Pentadecanoic acid $$ Pentadecylic acid $$ n-Pentadecanoic acid $$ n-Pentadecylic acid $$

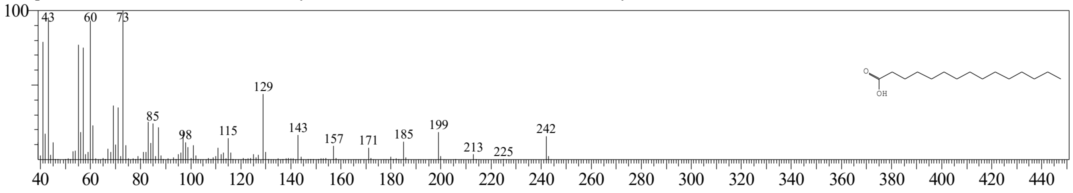

<< Target >>
Line#:35 R.Time:18.192(Scan#:1524) MassPeaks:192
RawMode:Single 18.192(1524) BasePeak:88.00(4920052)
BG Mode:None Group 1 - Event 1

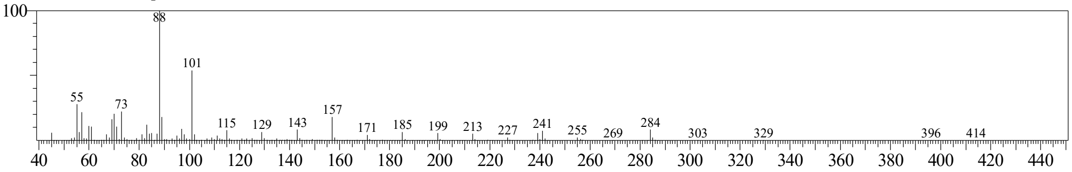

Hit#:1 Entry:106188 Library:NIST11.lib
SI:94 Formula:C18H36O2 CAS:628-97-7 MolWeight:284 RetIndex:1978
CompName:Hexadecanoic acid, ethyl ester $$ Palmitic acid, ethyl ester $$ Ethyl hexadecanoate $$ Ethyl palmitate $$ Ethyl n-hexadecanoate $$

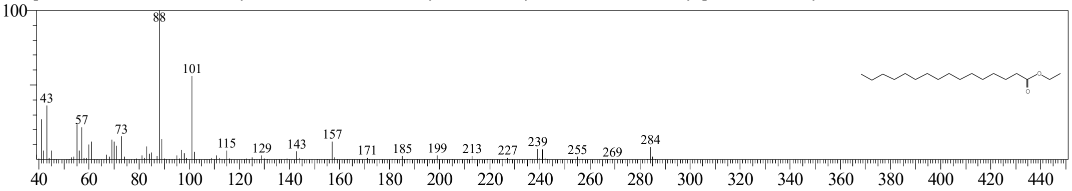

Hit#:2 Entry:95198 Library:NIST11.lib
SI:91 Formula:C17H34O2 CAS:0-00-0 MolWeight:270 RetIndex:1814
CompName:Ethyl 13-methyl-tetradecanoate

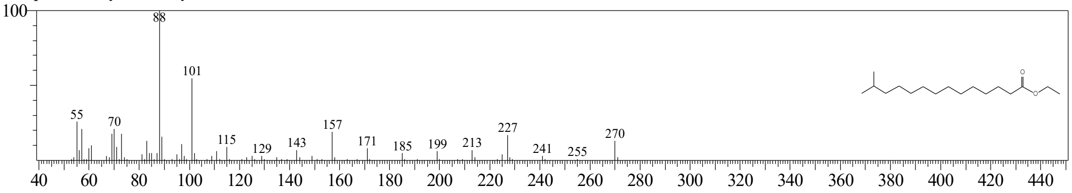

Hit#:3 Entry:25181 Library:NIST11s.lib
SI:90 Formula:C18H36O2 CAS:628-97-7 MolWeight:284 RetIndex:1978
CompName:Hexadecanoic acid, ethyl ester $$ Palmitic acid, ethyl ester $$ Ethyl hexadecanoate $$ Ethyl palmitate $$ Ethyl n-hexadecanoate $$

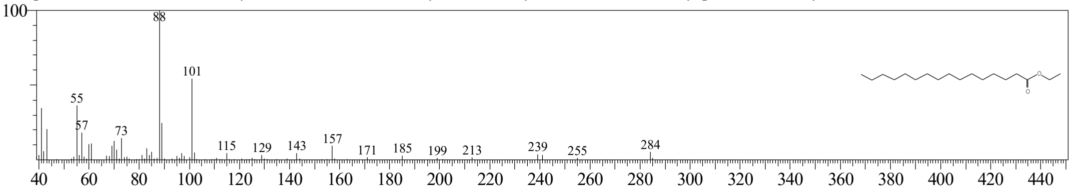

Hit#:4 Entry:95197 Library:NIST11.lib
SI:90 Formula:C17H34O2 CAS:41114-00-5 MolWeight:270 RetIndex:1878
CompName:Pentadecanoic acid, ethyl ester $$ Ethyl pentadecanoate $$ n-Pentadecanoic acid ethyl ester $$

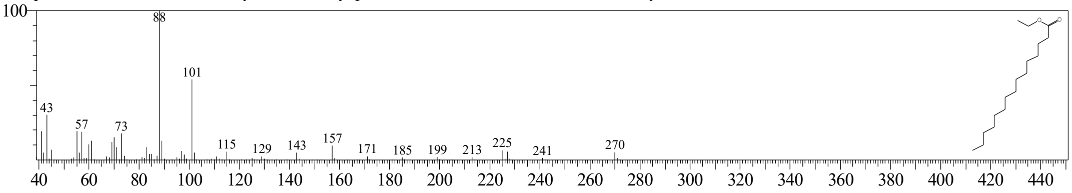

Hit#:5 Entry:26807 Library:NIST11s.lib
SI:90 Formula:C20H40O2 CAS:111-61-5 MolWeight:312 RetIndex:2177
CompName:Octadecanoic acid, ethyl ester $$ Stearic acid, ethyl ester $$ Ethyl n-octadecanoate $$ Ethyl octadecanoate $$ Ethyl stearate $$ Radia 7185 $$

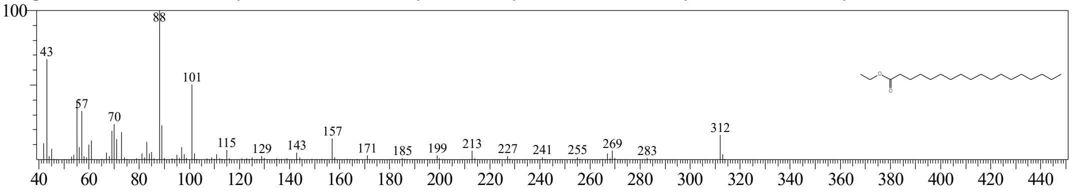

<< Target >>
Line#:36 R.Time:18.292(Scan#:1536) MassPeaks:207
RawMode:Single 18.292(1536) BasePeak:55.00(198759)
BG Mode:None Group 1 - Event 1

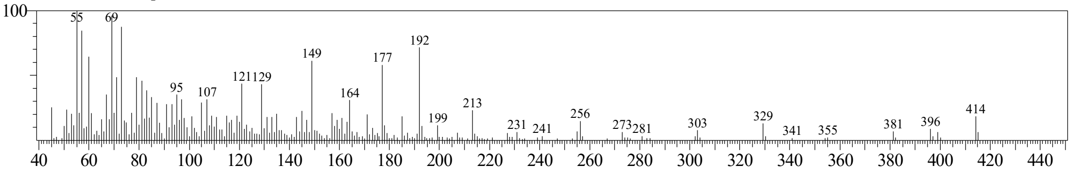

Hit#:1 Entry:29933 Library:NIST11s.lib
SI:63 Formula:C29H50O CAS:83-47-6 MolWeight:414 RetIndex:2731
CompName:.gamma.-Sitosterol $$ Stigmast-5-en-3-ol, (3.beta.,24S)- $$ Stigmast-5-en-3.beta.-ol, (24S)- $$ Clionasterol $$ Fucosterol, .beta.-dihydro- $$ 24.bet

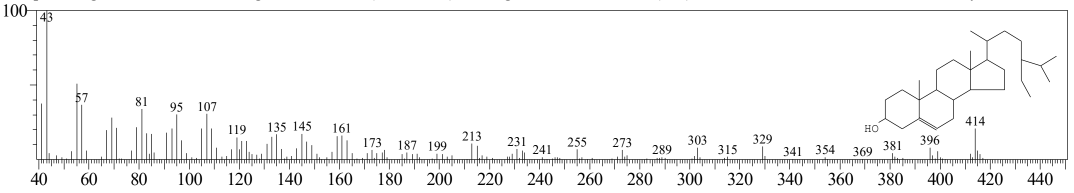

Hit#:2 Entry:187507 Library:NIST11.lib
SI:62 Formula:C29H50O CAS:83-46-5 MolWeight:414 RetIndex:2731
CompName:.beta.-Sitosterol $$ Stigmast-5-en-3-ol, (3.beta.)- $$ Stigmast-5-en-3.beta.-ol $$ .alpha.-Dihydrofucosterol $$ .beta.-Sitosterin $$ Angelicin $$ Ange

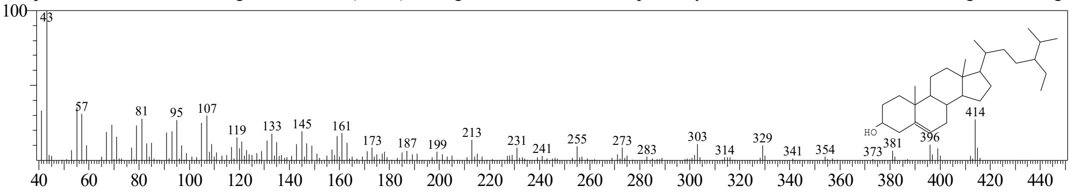

Hit#:3 Entry:29932 Library:NIST11s.lib
SI:62 Formula:C29H50O CAS:83-46-5 MolWeight:414 RetIndex:2731
CompName:.beta.-Sitosterol $$ Stigmast-5-en-3-ol, (3.beta.)- $$ Stigmast-5-en-3.beta.-ol $$ .alpha.-Dihydrofucosterol $$ .beta.-Sitosterin $$ Angelicin $$ Ange

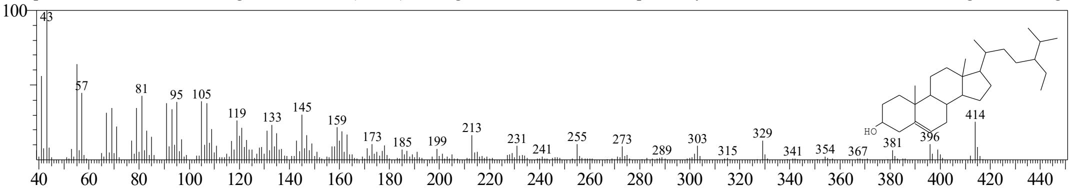

Hit#:4 Entry:187505 Library:NIST11.lib
SI:61 Formula:C29H50O CAS:0-00-0 MolWeight:414 RetIndex:2703
CompName:Stigmastan-3-en-6-ol $$ Stigmast-3-en-6-ol # $$

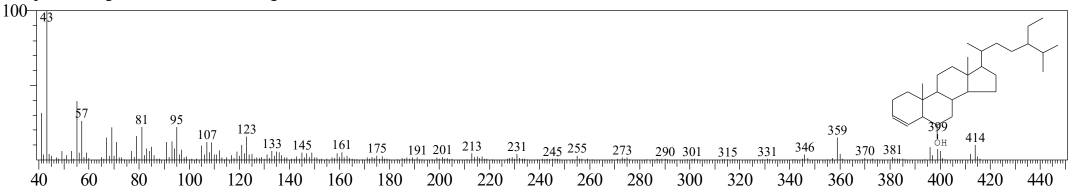

Hit#:5 Entry:187508 Library:NIST11.lib
SI:60 Formula:C29H50O CAS:83-47-6 MolWeight:414 RetIndex:2731
CompName:.gamma.-Sitosterol $$ Stigmast-5-en-3-ol, (3.beta.,24S)- $$ Stigmast-5-en-3.beta.-ol, (24S)- $$ Clionasterol $$ Fucosterol, .beta.-dihydro- $$ 24.bet

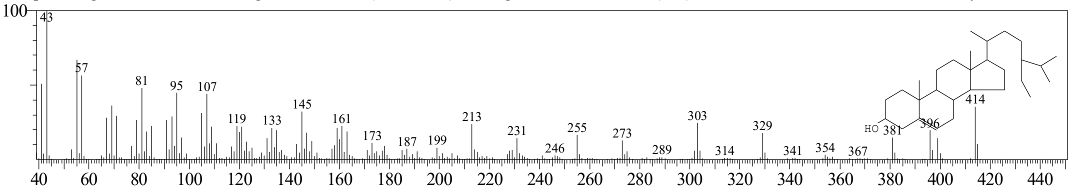

<< Target >>
Line#:37 R.Time:18.375(Scan#:1546) MassPeaks:215
RawMode:Single 18.375(1546) BasePeak:55.00(180927)
BG Mode:None Group 1 - Event 1

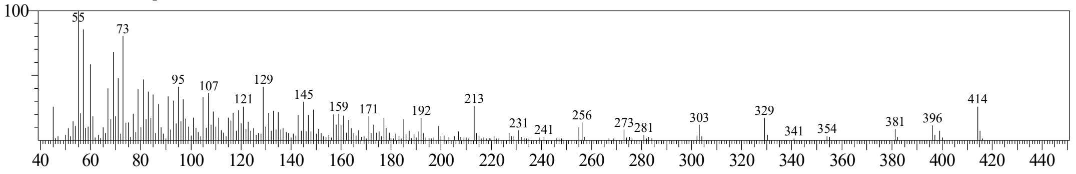

Hit#:1 Entry:29933 Library:NIST11s.lib
SI:73 Formula:C29H50O CAS:83-47-6 MolWeight:414 RetIndex:2731
CompName:.gamma.-Sitosterol $$ Stigmast-5-en-3-ol, (3.beta.,24S)- $$ Stigmast-5-en-3.beta.-ol, (24S)- $$ Clionasterol $$ Fucosterol, .beta.-dihydro- $$ 24.bet

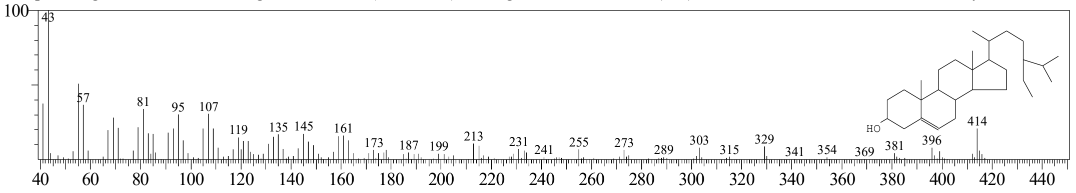

Hit#:2 Entry:29932 Library:NIST11s.lib
SI:71 Formula:C29H50O CAS:83-46-5 MolWeight:414 RetIndex:2731
CompName:.beta.-Sitosterol $$ Stigmast-5-en-3-ol, (3.beta.)- $$ Stigmast-5-en-3.beta.-ol $$ .alpha.-Dihydrofucosterol $$ .beta.-Sitosterin $$ Angelicin $$ Ange

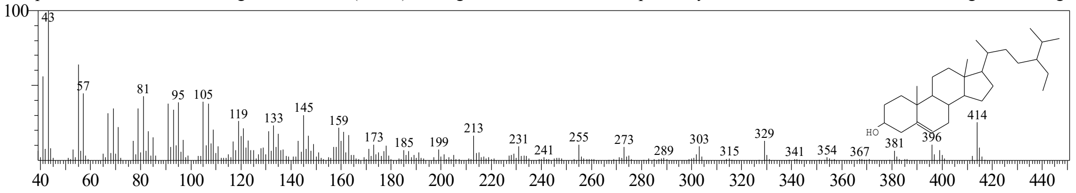

Hit#:3 Entry:187507 Library:NIST11.lib
SI:70 Formula:C29H50O CAS:83-46-5 MolWeight:414 RetIndex:2731
CompName:.beta.-Sitosterol $$ Stigmast-5-en-3-ol, (3.beta.)- $$ Stigmast-5-en-3.beta.-ol $$ .alpha.-Dihydrofucosterol $$ .beta.-Sitosterin $$ Angelicin $$ Ange

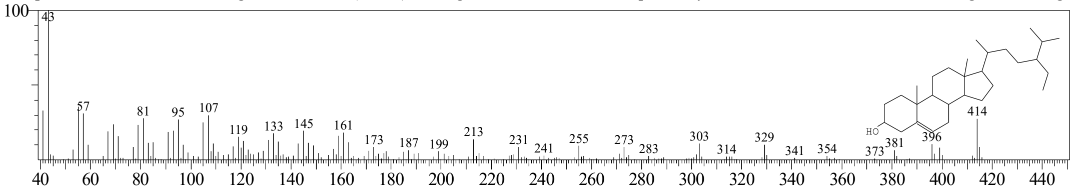

Hit#:4 Entry:187508 Library:NIST11.lib
SI:69 Formula:C29H50O CAS:83-47-6 MolWeight:414 RetIndex:2731
CompName:.gamma.-Sitosterol $$ Stigmast-5-en-3-ol, (3.beta.,24S)- $$ Stigmast-5-en-3.beta.-ol, (24S)- $$ Clionasterol $$ Fucosterol, .beta.-dihydro- $$ 24.bet

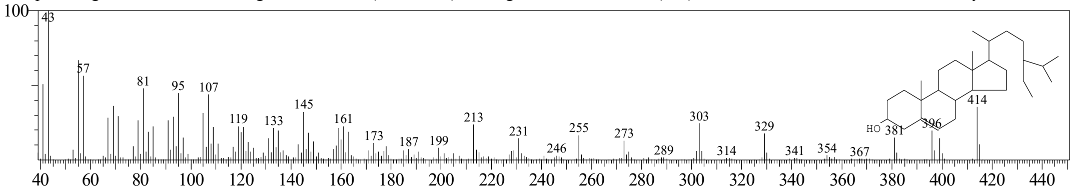

Hit#:5 Entry:187505 Library:NIST11.lib
SI:65 Formula:C29H50O CAS:0-00-0 MolWeight:414 RetIndex:2703
CompName:Stigmastan-3-en-6-ol $$ Stigmast-3-en-6-ol # $$

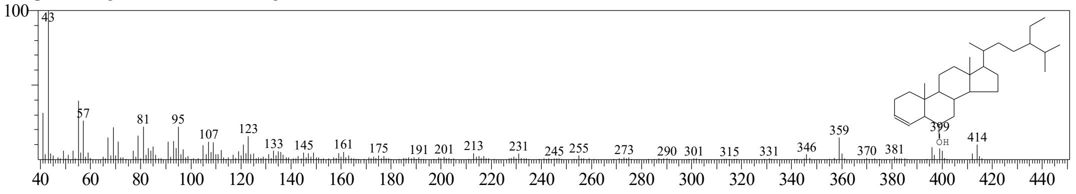

<< Target >>
Line#:38 R.Time:18.808(Scan#:1598) MassPeaks:190
RawMode:Single 18.808(1598) BasePeak:88.00(191967)
BG Mode:None Group 1 - Event 1

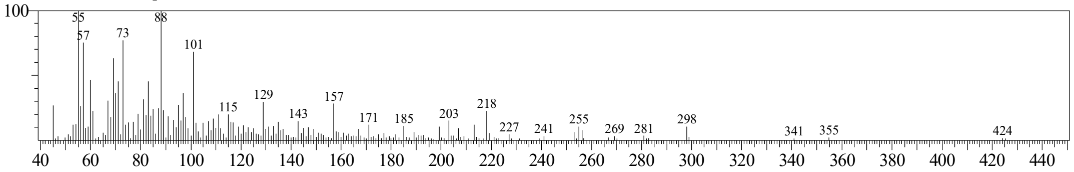

Hit#:1 Entry:51953 Library:NIST11.lib
SI:76 Formula:C11H16O4 CAS:117132-08-8 MolWeight:212 RetIndex:1610
CompName:9,9-Dimethoxybicyclo[3.3.1]nona-2,4-dione $$ 9,9-Dimethoxybicyclo[3.3.1]nonane-2,4-dione # $$

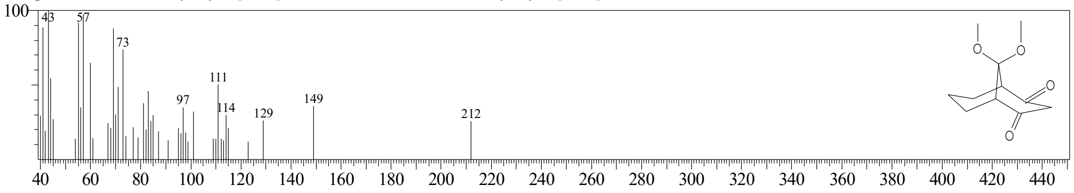

Hit#:2 Entry:117162 Library:NIST11.lib
SI:76 Formula:C19H38O2 CAS:0-00-0 MolWeight:298 RetIndex:2013
CompName:Ethyl 14-methyl-hexadecanoate

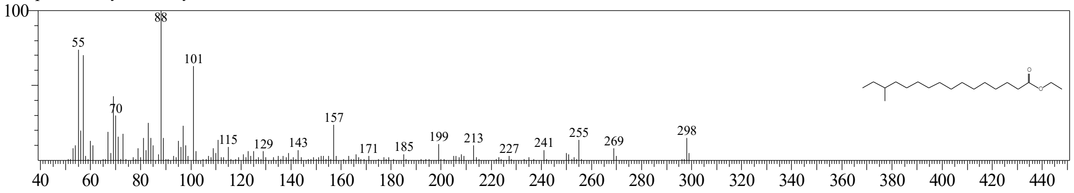

Hit#:3 Entry:43826 Library:NIST11.lib
SI:75 Formula:C11H20O3 CAS:3433-16-7 MolWeight:200 RetIndex:1470
CompName:Nonanoic acid, 9-oxo-, ethyl ester $$ Ethyl 9-oxononanoate # $$

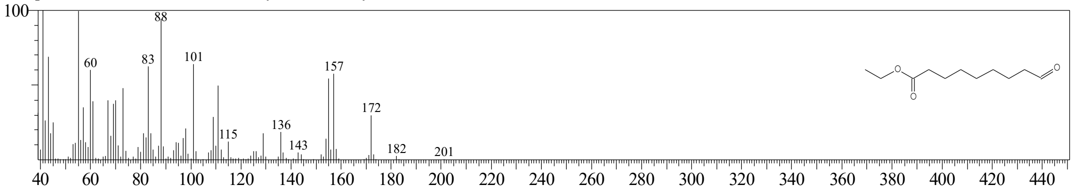

Hit#:4 Entry:128151 Library:NIST11.lib
SI:74 Formula:C20H40O2 CAS:506-30-9 MolWeight:312 RetIndex:2366
CompName:Eicosanoic acid $$ Arachic acid $$ Arachidic acid $$ Icosanoic acid $$ n-Eicosanoic acid $$ Arachidic acid (synthetic) $$

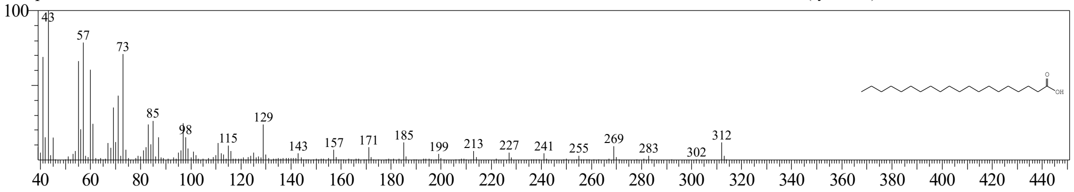

Hit#:5 Entry:42708 Library:NIST11.lib
SI:74 Formula:C12H22O2 CAS:0-00-0 MolWeight:198 RetIndex:1407
CompName:Propionic acid, 3-(2-methylcyclohexyl)-, ethyl ester $$ Ethyl 3-(2-methylcyclohexyl)propanoate # $$

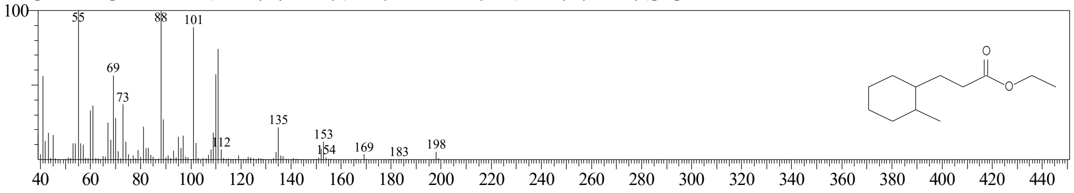

<< Target >>
Line#:39 R.Time:18.983(Scan#:1619) MassPeaks:191
RawMode:Single 18.983(1619) BasePeak:71.00(6303335)
BG Mode:None Group 1 - Event 1

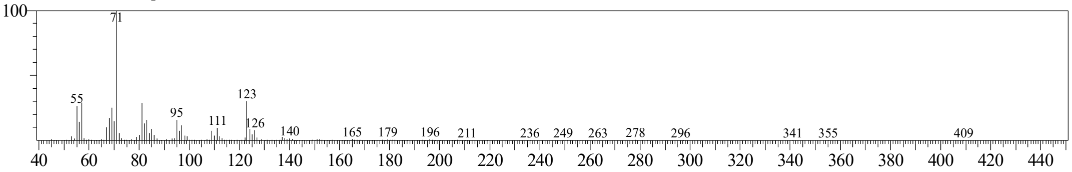

Hit#:1 Entry:115516 Library:NIST11.lib
SI:97 Formula:C20H40O CAS:150-86-7 MolWeight:296 RetIndex:2045
CompName:Phytol $$ 2-Hexadecen-1-ol, 3,7,11,15-tetramethyl-, [R-[R*,R*-(E)]]- $$ trans-Phytol $$ 3,7,11,15-Tetramethyl-2-hexadecen-1-ol-, (2E,7R,11R)- $

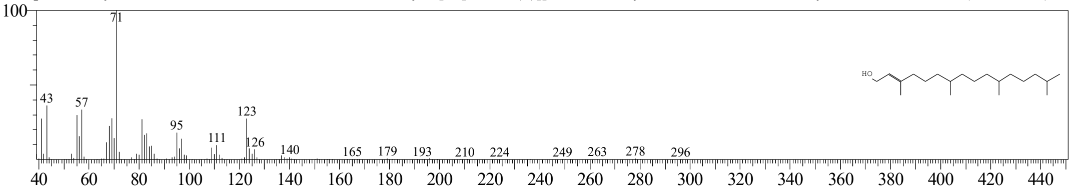

Hit#:2 Entry:25943 Library:NIST11s.lib
SI:95 Formula:C20H40O CAS:150-86-7 MolWeight:296 RetIndex:2045
CompName:Phytol $$ 2-Hexadecen-1-ol, 3,7,11,15-tetramethyl-, [R-[R*,R*-(E)]]- $$ trans-Phytol $$ 3,7,11,15-Tetramethyl-2-hexadecen-1-ol-, (2E,7R,11R)- $

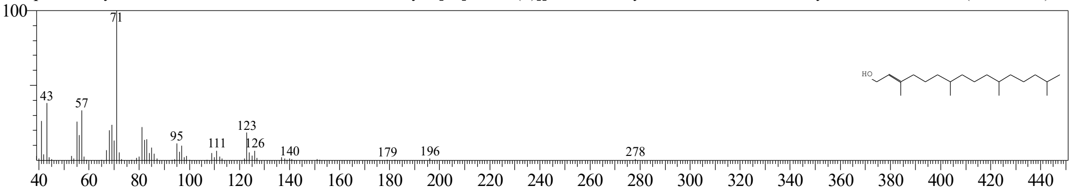

Hit#:3 Entry:25942 Library:NIST11s.lib
SI:95 Formula:C20H40O CAS:150-86-7 MolWeight:296 RetIndex:2045
CompName:Phytol $$ 2-Hexadecen-1-ol, 3,7,11,15-tetramethyl-, [R-[R*,R*-(E)]]- $$ trans-Phytol $$ 3,7,11,15-Tetramethyl-2-hexadecen-1-ol-, (2E,7R,11R)- $

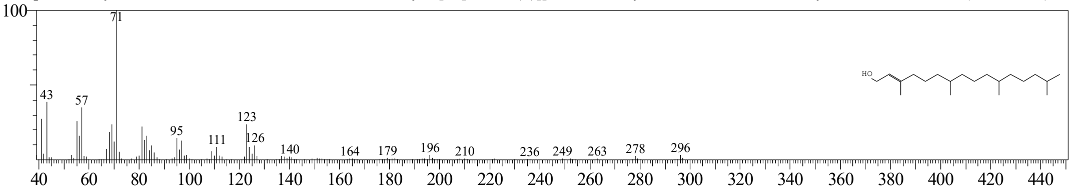

Hit#:4 Entry:10360 Library:NIST11s.lib
SI:87 Formula:C10H20O CAS:490-99-3 MolWeight:156 RetIndex:1164
CompName:Cyclohexanol, 5-methyl-2-(1-methylethyl)-, (1.alpha.,2.beta.,5.beta.)- $$ Menthol, trans-1,3,cis-1,4- $$ Isomenthol $$ Isomentol $$ 2-Isopropyl-5-n

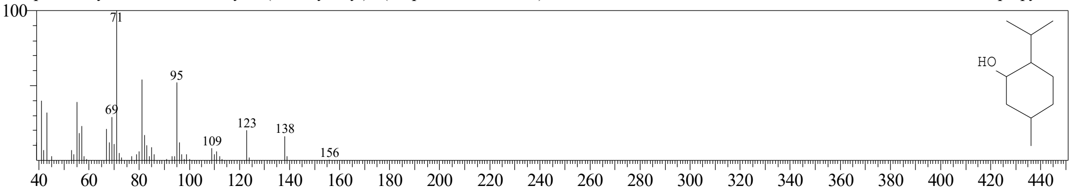

Hit#:5 Entry:18670 Library:NIST11.lib
SI:86 Formula:C10H20O CAS:23283-97-8 MolWeight:156 RetIndex:1164
CompName:Cyclohexanol, 5-methyl-2-(1-methylethyl)-, [1S-(1.alpha.,2.beta.,5.beta.)]- $$ (1S,2R,5R)-(+)-Isomenthol $$ 2-Isopropyl-5-methylcyclohexanol # $

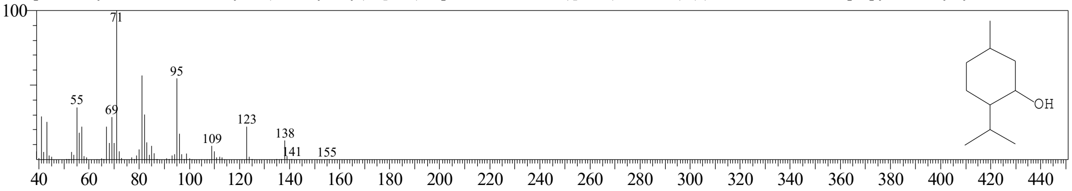

<< Target >>
Line#:40 R.Time:19.267(Scan#:1653) MassPeaks:223
RawMode:Single 19.267(1653) BasePeak:67.00(1478470)
BG Mode:None Group 1 - Event 1

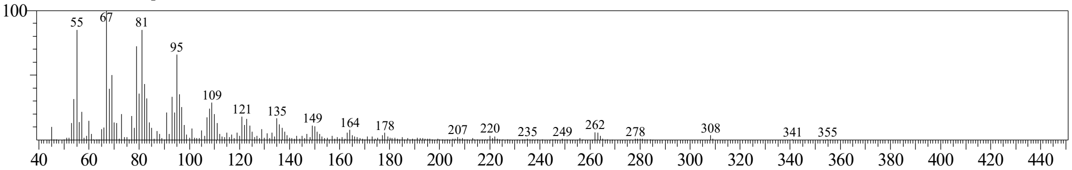

Hit#:1 Entry:144979 Library:NIST11.lib
SI:90 Formula:C22H38O2 CAS:10152-71-3 MolWeight:334 RetIndex:2266
CompName:Cyclopropaneoctanoic acid, 2-[[2-[(2-ethylcyclopropyl)methyl]cyclopropyl]methyl]-, methyl ester $$ Methyl 8-[2-((2-[(2-ethylcyclopropyl)methyl]c

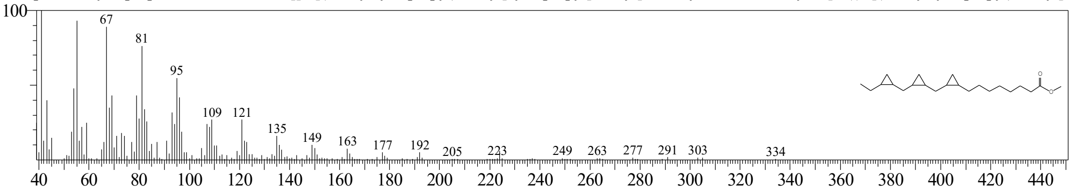

Hit#:2 Entry:24902 Library:NIST11s.lib
SI:89 Formula:C18H32O2 CAS:60-33-3 MolWeight:280 RetIndex:2183
CompName:9,12-Octadecadienoic acid (Z,Z)- $$ cis-9,cis-12-Octadecadienoic acid $$ cis,cis-Linoleic acid $$ Grape seed oil $$ Linoleic $$ Linoleic acid $$ Li

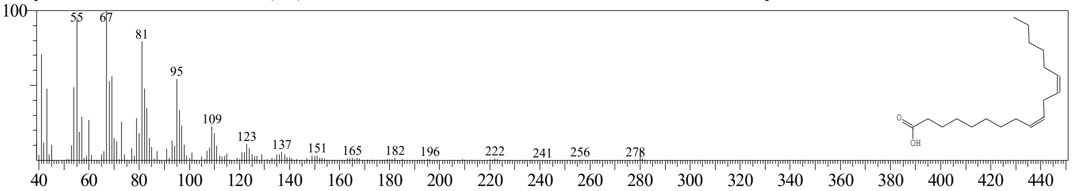

Hit#:3 Entry:126507 Library:NIST11.lib
SI:89 Formula:C20H38O2 CAS:17367-08-7 MolWeight:310 RetIndex:2344
CompName:Ethanol, 2-(9,12-octadecadienyloxy)-, (Z,Z)- $$ 2-cis,cis-9,12-Octadecadienyloxyethanol $$ 2-[(9Z,12Z)-9,12-Octadecadienyloxy]ethanol # $$

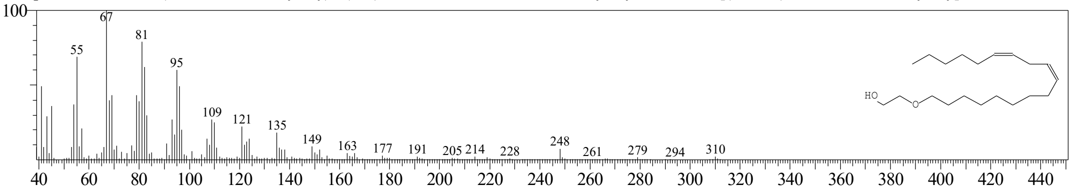

Hit#:4 Entry:81324 Library:NIST11.lib
SI:89 Formula:C17H32O CAS:64566-18-3 MolWeight:252 RetIndex:1907
CompName:(R)-(-)-14-Methyl-8-hexadecyn-1-ol $$ 14-Methyl-8-hexadecyn-1-ol # $$

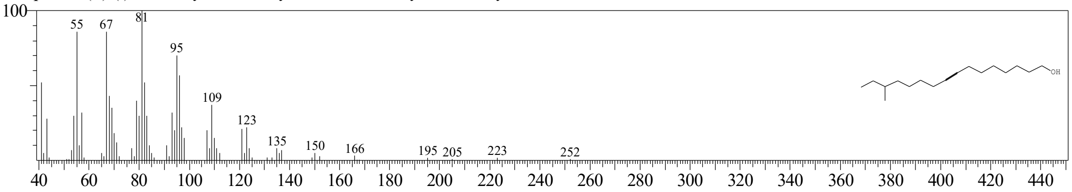

Hit#:5 Entry:24903 Library:NIST11s.lib
SI:88 Formula:C18H32O2 CAS:60-33-3 MolWeight:280 RetIndex:2183
CompName:9,12-Octadecadienoic acid (Z,Z)- $$ cis-9,cis-12-Octadecadienoic acid $$ cis,cis-Linoleic acid $$ Grape seed oil $$ Linoleic $$ Linoleic acid $$ Li

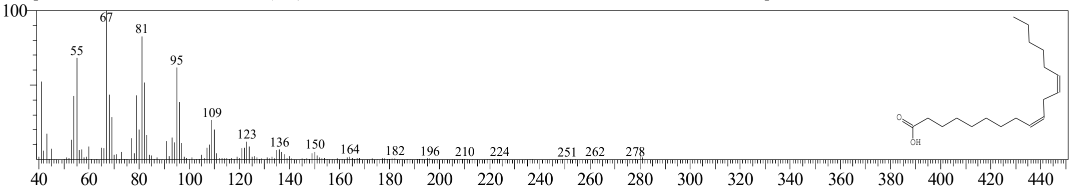

<< Target >>
Line#:41 R.Time:19.317(Scan#:1659) MassPeaks:225
RawMode:Single 19.317(1659) BasePeak:55.00(1747105)
BG Mode:None Group 1 - Event 1

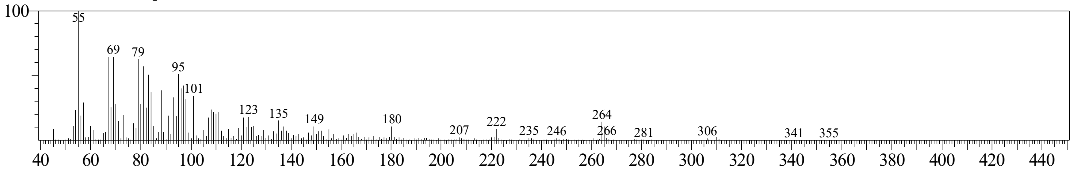

Hit#:1 Entry:26691 Library:NIST11s.lib
SI:82 Formula:C20H38O2 CAS:111-62-6 MolWeight:310 RetIndex:2185
CompName:Ethyl Oleate $$ 9-Octadecenoic acid (Z)-, ethyl ester $$ Oleic acid, ethyl ester $$ (Z)-9-Octadecenoic acid ethyl ester $$ Ethyl cis-9-octadecenoate

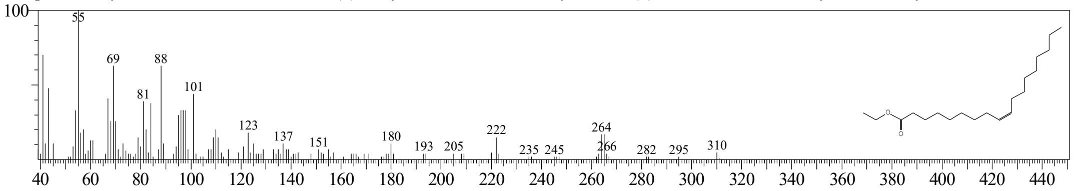

Hit#:2 Entry:26694 Library:NIST11s.lib
SI:81 Formula:C20H38O2 CAS:111-62-6 MolWeight:310 RetIndex:2185
CompName:Ethyl Oleate $$ 9-Octadecenoic acid (Z)-, ethyl ester $$ Oleic acid, ethyl ester $$ (Z)-9-Octadecenoic acid ethyl ester $$ Ethyl cis-9-octadecenoate

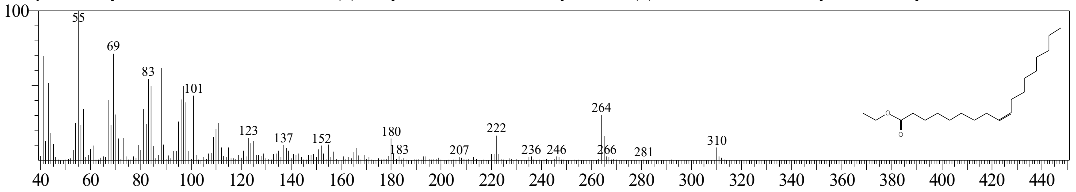

Hit#:3 Entry:126491 Library:NIST11.lib
SI:81 Formula:C20H38O2 CAS:6114-18-7 MolWeight:310 RetIndex:2185
CompName:(E)-9-Octadecenoic acid ethyl ester $$ 9-Octadecenoic acid, ethyl ester, (E)- $$ Ethyl 9-octadecenoate, (E)- $$ Ethyl (9E)-9-octadecenoate $$ Elaid

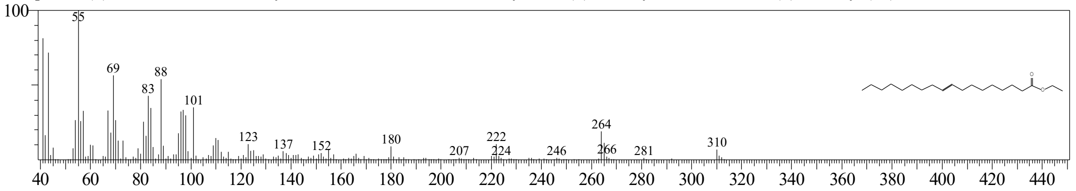

Hit#:4 Entry:26693 Library:NIST11s.lib
SI:80 Formula:C20H38O2 CAS:111-62-6 MolWeight:310 RetIndex:2185
CompName:Ethyl Oleate $$ 9-Octadecenoic acid (Z)-, ethyl ester $$ Oleic acid, ethyl ester $$ (Z)-9-Octadecenoic acid ethyl ester $$ Ethyl cis-9-octadecenoate

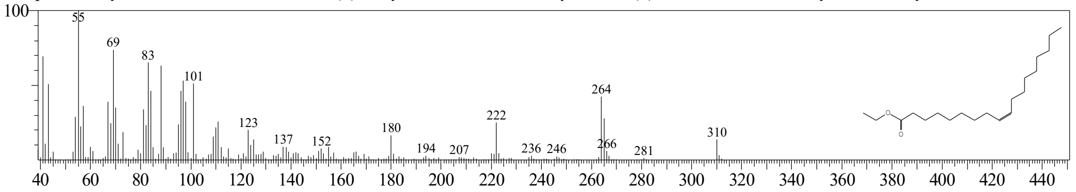

Hit#:5 Entry:159418 Library:NIST11.lib
SI:80 Formula:C21H40O4 CAS:3443-84-3 MolWeight:356 RetIndex:2705
CompName:9-Octadecenoic acid (Z)-, 2-hydroxy-1-(hydroxymethyl)ethyl ester $$ Olein, 2-mono- $$ .beta.-Monoolein $$ Glycerol 2-monooleate $$ 2-Monoole

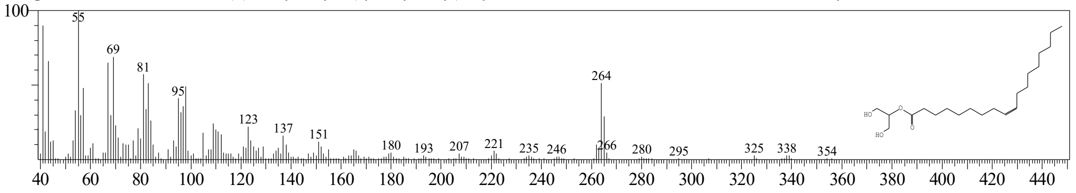

<< Target >>
Line#:42 R.Time:19.442(Scan#:1674) MassPeaks:212
RawMode:Single 19.442(1674) BasePeak:88.00(2287434)
BG Mode:None Group 1 - Event 1

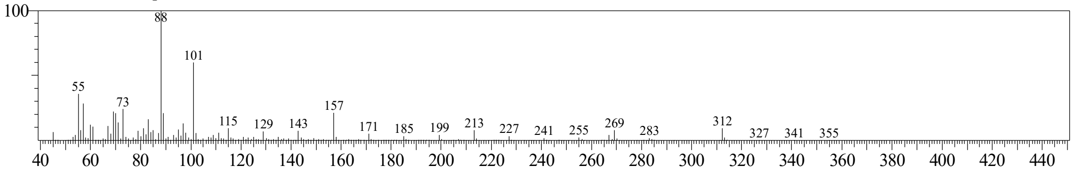

Hit#:1 Entry:26807 Library:NIST11s.lib
SI:90 Formula:C20H40O2 CAS:111-61-5 MolWeight:312 RetIndex:2177
CompName:Octadecanoic acid, ethyl ester $$ Stearic acid, ethyl ester $$ Ethyl n-octadecanoate $$ Ethyl octadecanoate $$ Ethyl stearate $$ Radia 7185 $$

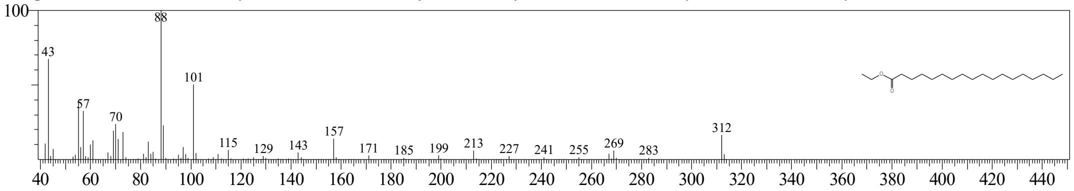

Hit#:2 Entry:26809 Library:NIST11s.lib
SI:88 Formula:C20H40O2 CAS:111-61-5 MolWeight:312 RetIndex:2177
CompName:Octadecanoic acid, ethyl ester $$ Stearic acid, ethyl ester $$ Ethyl n-octadecanoate $$ Ethyl octadecanoate $$ Ethyl stearate $$ Radia 7185 $$

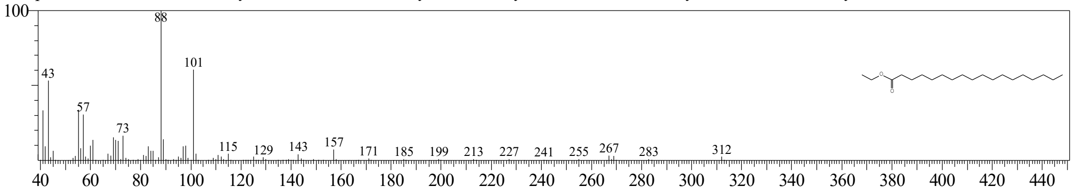

Hit#:3 Entry:26811 Library:NIST11s.lib
SI:88 Formula:C20H40O2 CAS:55124-97-5 MolWeight:312 RetIndex:2112
CompName:Octadecanoic acid, 17-methyl-, methyl ester $$ Methyl 17-methyloctadecanoate # $$

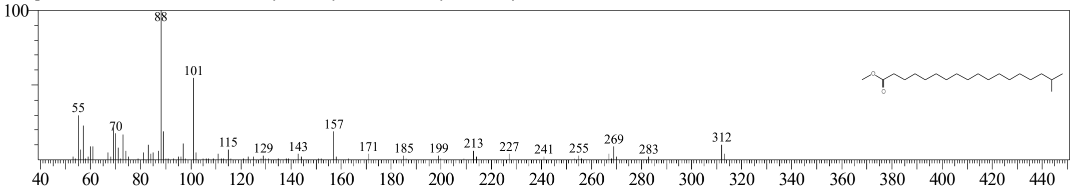

Hit#:4 Entry:117164 Library:NIST11.lib
SI:87 Formula:C19H38O2 CAS:0-00-0 MolWeight:298 RetIndex:2013
CompName:Ethyl 15-methyl-hexadecanoate

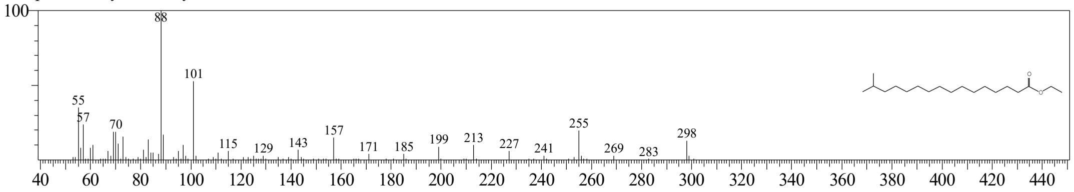

Hit#:5 Entry:26810 Library:NIST11s.lib
SI:87 Formula:C20H40O2 CAS:111-61-5 MolWeight:312 RetIndex:2177
CompName:Octadecanoic acid, ethyl ester $$ Stearic acid, ethyl ester $$ Ethyl n-octadecanoate $$ Ethyl octadecanoate $$ Ethyl stearate $$ Radia 7185 $$

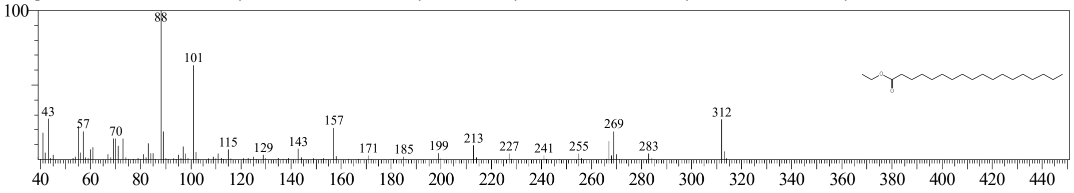

<< Target >>
Line#:43 R.Time:19.833(Scan#:1721) MassPeaks:214
RawMode:Single 19.833(1721) BasePeak:218.10(222500)
BG Mode:None Group 1 - Event 1

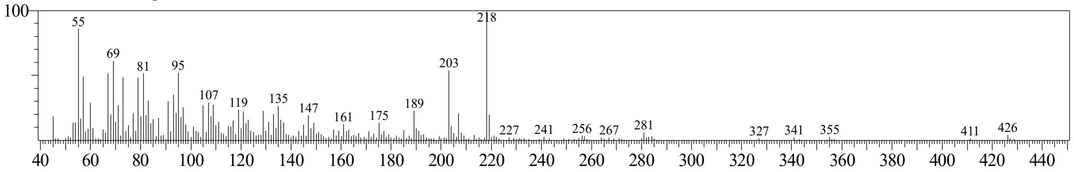

Hit#:1 Entry:120419 Library:NIST11.lib
SI:72 Formula:C21H34O CAS:56052-96-1 MolWeight:302 RetIndex:2075
CompName:Androstan-17-one, 16,16-dimethyl-, (5.alpha.)- $$ 16,16-Dimethylandrostan-17-one # $$

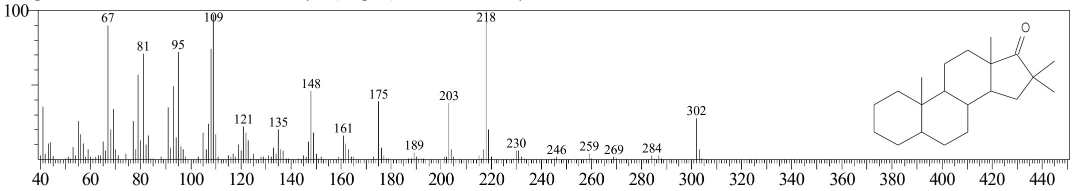

Hit#:2 Entry:99975 Library:NIST11.lib
SI:69 Formula:C19H32O CAS:54411-60-8 MolWeight:276 RetIndex:1953
CompName:D-Norandrostane-16-methanol, (5.alpha.,16.beta.)- $$ (8a,10a-Dimethylhexadecahydrocyclobuta[a]phenanthren-1-yl)methanol # $$

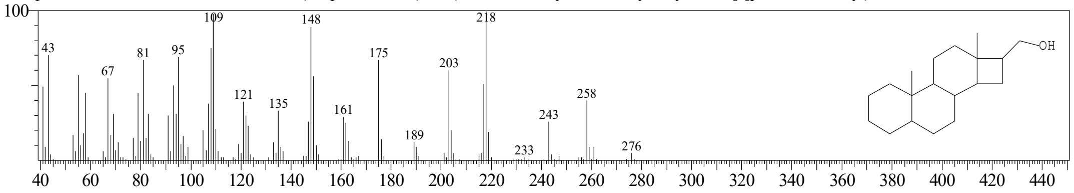

Hit#:3 Entry:110844 Library:NIST11.lib
SI:69 Formula:C19H30O2 CAS:32319-09-8 MolWeight:290 RetIndex:2067
CompName:D-Norandrostane-16-carboxylic acid, (5.alpha.,16.beta.)- $$ 8a,10a-Dimethylhexadecahydrocyclobuta[a]phenanthrene-1-carboxylic acid # $$

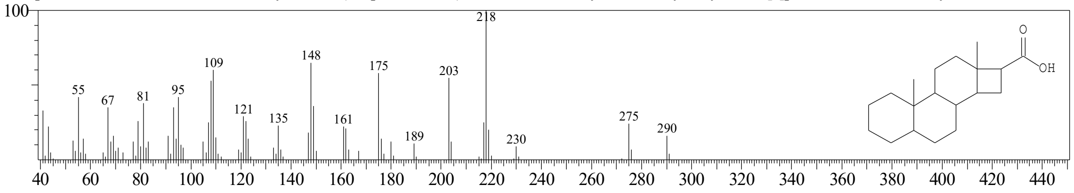

Hit#:4 Entry:186088 Library:NIST11.lib
SI:68 Formula:C30H50 CAS:464-97-1 MolWeight:410 RetIndex:2685
CompName:Urs-12-ene

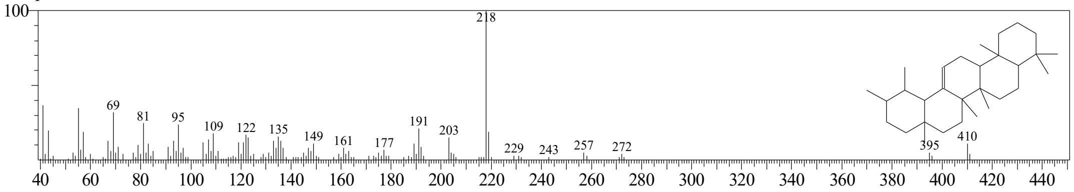

Hit#:5 Entry:56374 Library:NIST11.lib
SI:68 Formula:C15H22O CAS:0-00-0 MolWeight:218 RetIndex:1673
CompName:2(1H)Naphthalenone, 3,5,6,7,8,8a-hexahydro-4,8a-dimethyl-6-(1-methylethenyl)- $$ 6-Isopropenyl-4,8a-dimethyl-3,5,6,7,8,8a-hexahydro-2(1H)-na

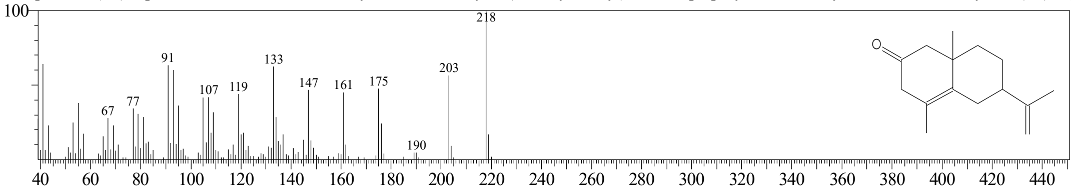

<< Target >>
Line#:44 R.Time:20.092(Scan#:1752) MassPeaks:213
RawMode:Single 20.092(1752) BasePeak:55.00(173390)
BG Mode:None Group 1 - Event 1

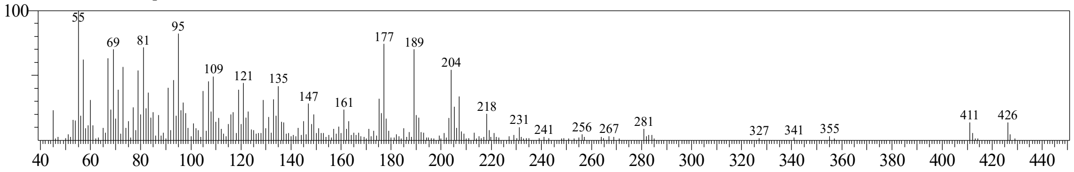

Hit#:1 Entry:59363 Library:NIST11.lib
SI:72 Formula:C15H26O CAS:473-04-1 MolWeight:222 RetIndex:1647
CompName:1-Naphthalenol, decahydro-1,4a-dimethyl-7-(1-methylethylidene)-, [1R-(1.alpha.,4a.beta.,8a.alpha.)]- $$ Eudesm-7(11)-en-4-ol $$ Juniper camphor

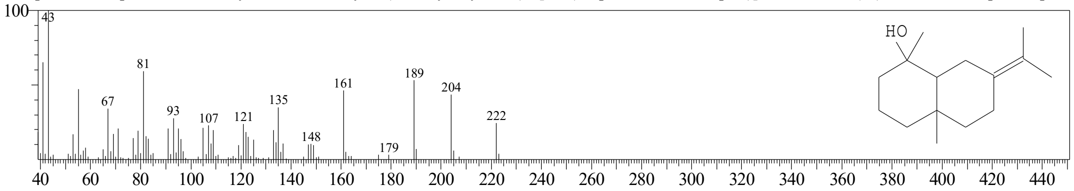

Hit#:2 Entry:89013 Library:NIST11.lib
SI:71 Formula:C18H30O CAS:0-00-0 MolWeight:262 RetIndex:1825
CompName:Sclareoloxide(Cis-B/C) $$ 3,4a,7,7,10a-Pentamethyl-4a,5,6,6a,7,8,9,10,10a,10b-decahydro-1H-benzo[f]chromene # $$

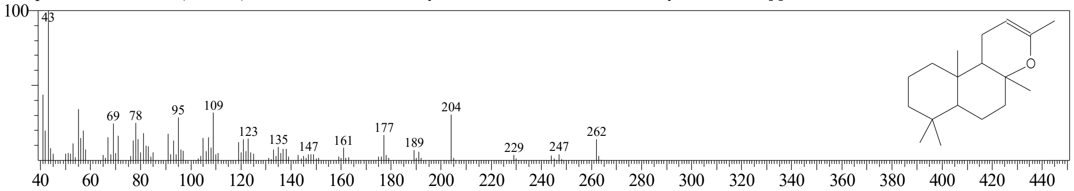

Hit#:3 Entry:59458 Library:NIST11.lib
SI:71 Formula:C15H26O CAS:465-24-7 MolWeight:222 RetIndex:1593
CompName:1,4-Methanoazulen-9-ol, decahydro-1,5,5,8a-tetramethyl-, [1R-(1.alpha.,3a.beta.,4.alpha.,8a.beta.,9S*)]- $$ Longiborneol $$ Juniperol $$

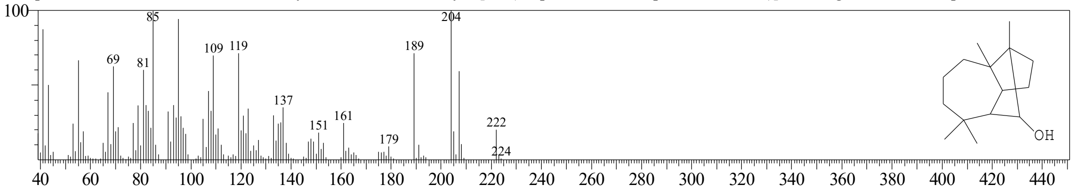

Hit#:4 Entry:57718 Library:NIST11.lib
SI:71 Formula:C15H24O CAS:0-00-0 MolWeight:220 RetIndex:1462
CompName:Aromadendrene oxide-(2)

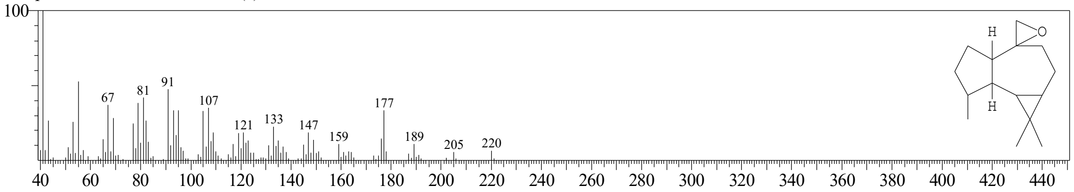

Hit#:5 Entry:46768 Library:NIST11.lib
SI:71 Formula:C15H24 CAS:473-13-2 MolWeight:204 RetIndex:1474
CompName:Naphthalene, 1,2,3,4,4a,5,6,8a-octahydro-4a,8-dimethyl-2-(1-methylethenyl)-, [2R-(2.alpha.,4a.alpha.,8a.beta.)]- $$ .alpha.-Selinene $$ 2-Isoproper

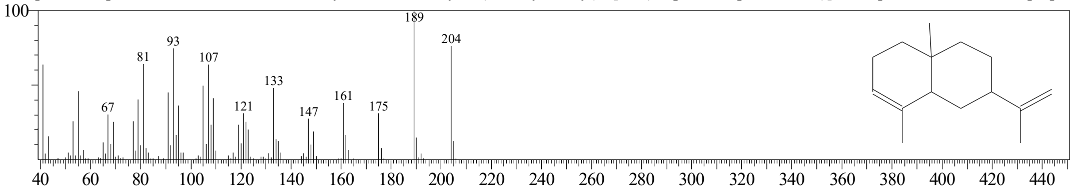

<< Target >>
Line#:45 R.Time:20.392(Scan#:1788) MassPeaks:214
RawMode:Single 20.392(1788) BasePeak:218.10(185132)
BG Mode:None Group 1 - Event 1

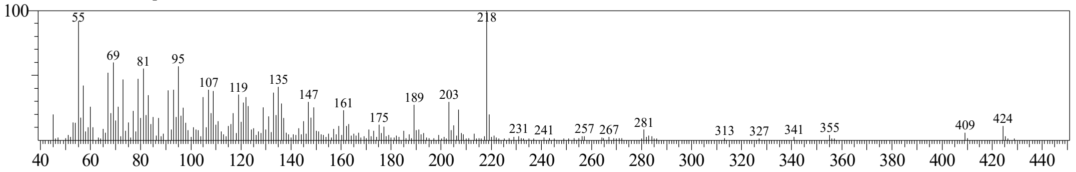

Hit#:1 Entry:120419 Library:NIST11.lib
SI:73 Formula:C21H34O CAS:56052-96-1 MolWeight:302 RetIndex:2075
CompName:Androstan-17-one, 16,16-dimethyl-, (5.alpha.)- $$ 16,16-Dimethylandrostan-17-one # $$

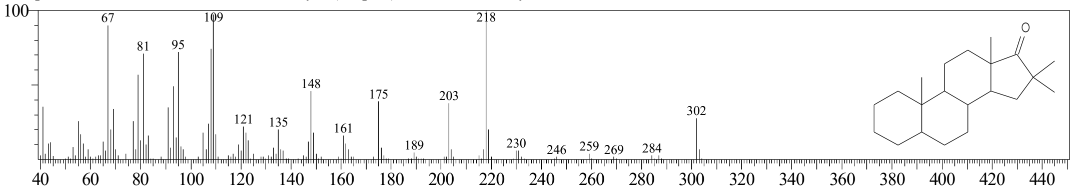

Hit#:2 Entry:101296 Library:NIST11.lib
SI:72 Formula:C17H26O3 CAS:0-00-0 MolWeight:278 RetIndex:2018
CompName:Acetic acid, 3-hydroxy-6-isopropenyl-4,8a-dimethyl-1,2,3,5,6,7,8,8a-octahydronaphthalen-2-yl ester $$ 3-Hydroxy-6-isopropenyl-4,8a-dimethyl-1,

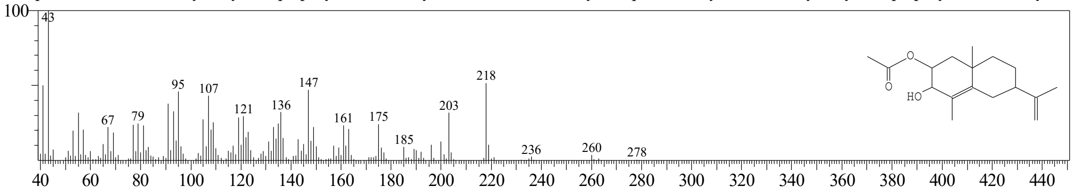

Hit#:3 Entry:101320 Library:NIST11.lib
SI:72 Formula:C17H26O3 CAS:0-00-0 MolWeight:278 RetIndex:2018
CompName:Acetic acid, 3-hydroxy-7-isopropenyl-1,4a-dimethyl-2,3,4,4a,5,6,7,8-octahydronaphthalen-2-yl ester $$ 3-Hydroxy-7-isopropenyl-1,4a-dimethyl-2,

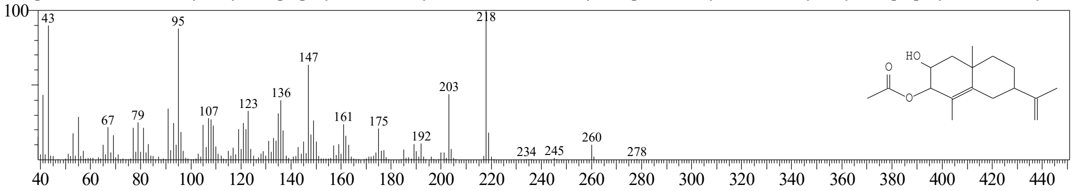

Hit#:4 Entry:99975 Library:NIST11.lib
SI:71 Formula:C19H32O CAS:54411-60-8 MolWeight:276 RetIndex:1953
CompName:D-Norandrostane-16-methanol, (5.alpha.,16.beta.)- $$ (8a,10a-Dimethylhexadecahydrocyclobuta[a]phenanthren-1-yl)methanol # $$

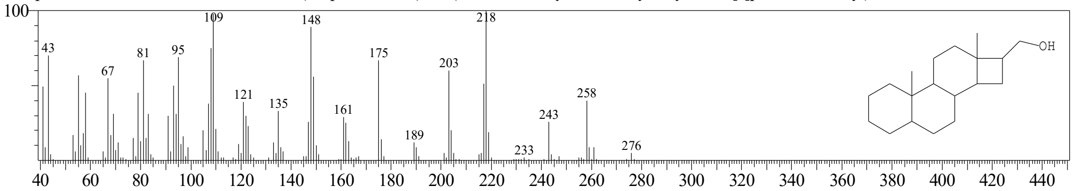

Hit#:5 Entry:110844 Library:NIST11.lib
SI:70 Formula:C19H30O2 CAS:32319-09-8 MolWeight:290 RetIndex:2067
CompName:D-Norandrostane-16-carboxylic acid, (5.alpha.,16.beta.)- $$ 8a,10a-Dimethylhexadecahydrocyclobuta[a]phenanthrene-1-carboxylic acid # $$

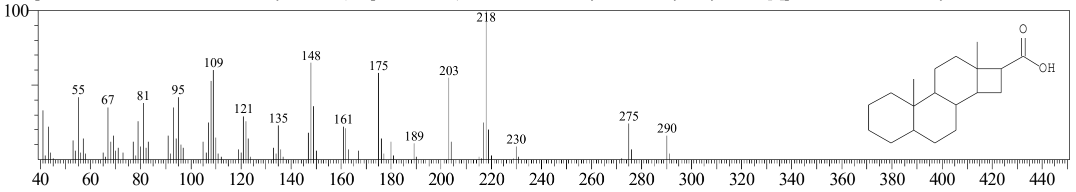

<< Target >>
Line#:46 R.Time:20.650(Scan#:1819) MassPeaks:207
RawMode:Single 20.650(1819) BasePeak:88.00(467740)
BG Mode:None Group 1 - Event 1

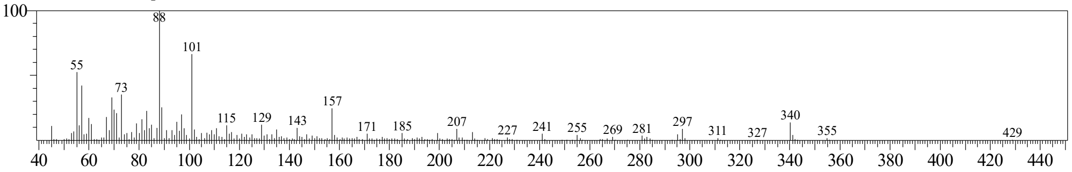

Hit#:1 Entry:25180 Library:NIST11s.lib
SI:84 Formula:C18H36O2 CAS:628-97-7 MolWeight:284 RetIndex:1978
CompName:Hexadecanoic acid, ethyl ester $$ Palmitic acid, ethyl ester $$ Ethyl hexadecanoate $$ Ethyl palmitate $$ Ethyl n-hexadecanoate $$

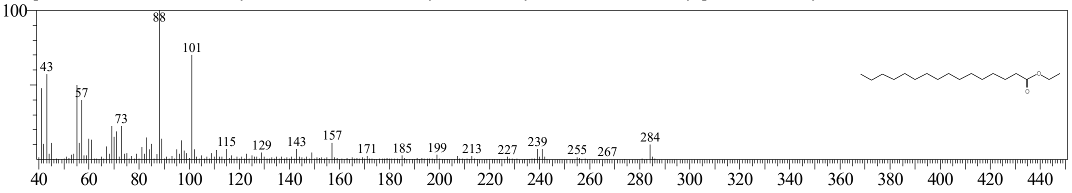

Hit#:2 Entry:117162 Library:NIST11.lib
SI:83 Formula:C19H38O2 CAS:0-00-0 MolWeight:298 RetIndex:2013
CompName:Ethyl 14-methyl-hexadecanoate

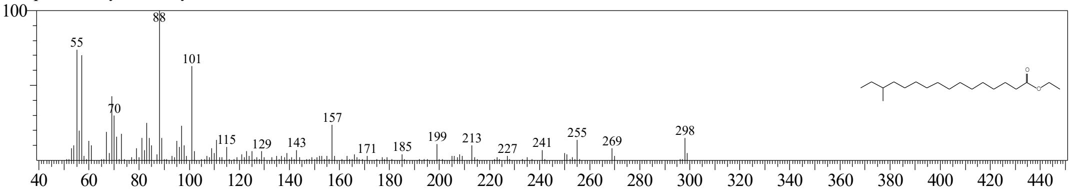

Hit#:3 Entry:166830 Library:NIST11.lib
SI:81 Formula:C24H48O2 CAS:5908-87-2 MolWeight:368 RetIndex:2574
CompName:Docosanoic acid, ethyl ester $$ Ethyl docosanoate # $$

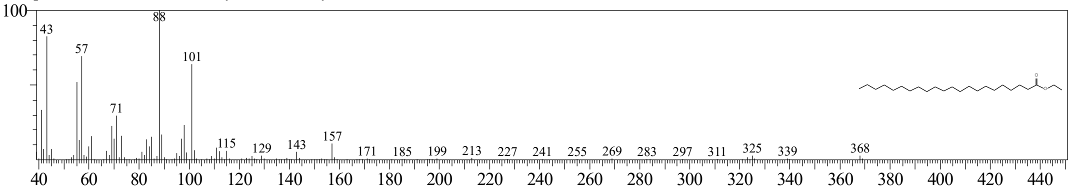

Hit#:4 Entry:73692 Library:NIST11.lib
SI:80 Formula:C14H26O3 CAS:151271-75-9 MolWeight:242 RetIndex:1769
CompName:12-Oxododecanoic acid, ethyl ester $$ Ethyl 12-oxododecanoate # $$

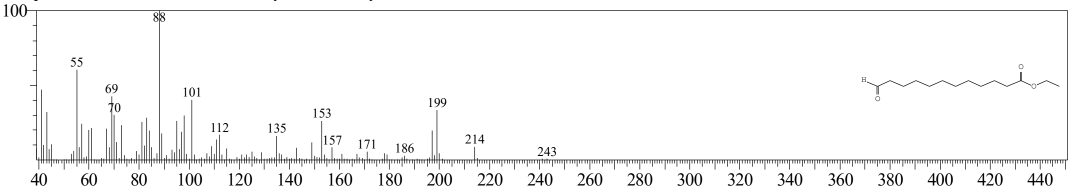

Hit#:5 Entry:73860 Library:NIST11.lib
SI:79 Formula:C15H30O2 CAS:28267-29-0 MolWeight:242 RetIndex:1680
CompName:Ethyl tridecanoate $$ Tridecanoic acid ethyl ester $$ n-Tridecanoic acid ethyl ester $$

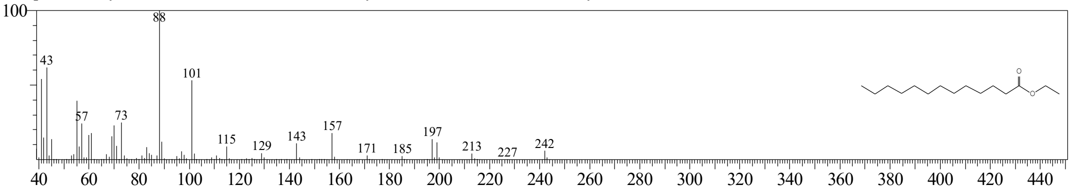

<< Target >>
Line#:47 R.Time:20.725(Scan#:1828) MassPeaks:192
RawMode:Single 20.725(1828) BasePeak:55.00(143513)
BG Mode:None Group 1 - Event 1

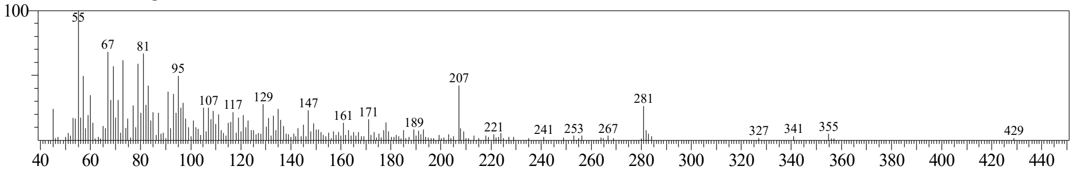

Hit#:1 Entry:102809 Library:NIST11.lib
SI:73 Formula:C18H32O2 CAS:34450-18-5 MolWeight:280 RetIndex:2165
CompName:17-Octadecynoic acid

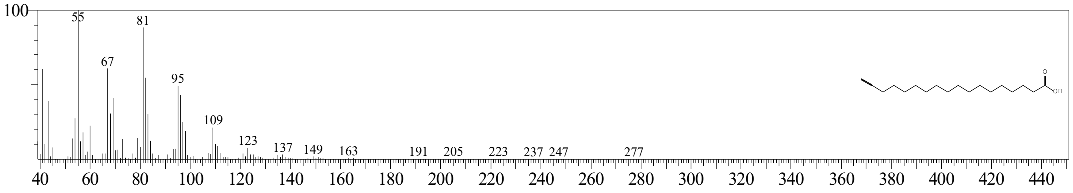

Hit#:2 Entry:135733 Library:NIST11.lib
SI:72 Formula:C21H38O2 CAS:56687-68-4 MolWeight:322 RetIndex:2203
CompName:[1,1'-Bicyclopropyl]-2-octanoic acid, 2'-hexyl-, methyl ester

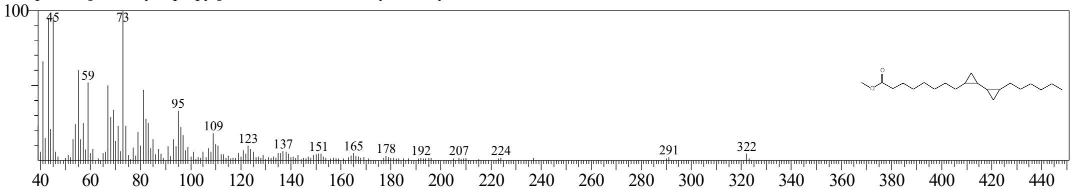

Hit#:3 Entry:203338 Library:NIST11.lib
SI:71 Formula:C30H52O3Si CAS:55759-94-9 MolWeight:488 RetIndex:3258
CompName:9,10-Secocholesta-5,7,10(19)-triene-1,3-diol, 25-[(trimethylsilyl)oxy]-, (3.beta.,5Z,7E)- $$ (5E,7E)-25-[(Trimethylsilyl)oxy]-9,10-secocholesta-5,7.

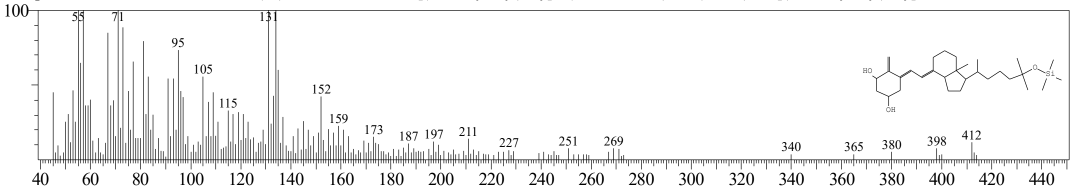

Hit#:4 Entry:174146 Library:NIST11.lib
SI:70 Formula:C22H42O3Si CAS:22074-68-6 MolWeight:382 RetIndex:2377
CompName:9,12-Octadecadienoic acid, 18-(trimethylsiloxy)-, methyl ester $$ Methyl (9E,12E)-18-[(trimethylsilyl)oxy]-9,12-octadecadienoate # $$

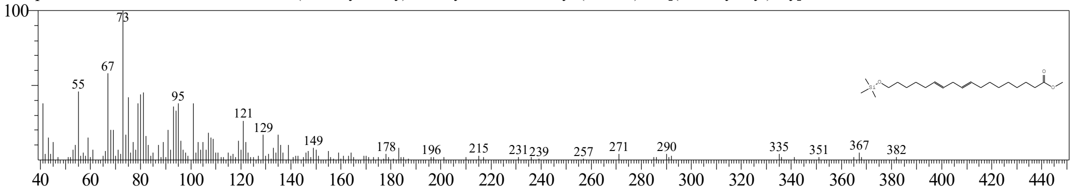

Hit#:5 Entry:90501 Library:NIST11.lib
SI:69 Formula:C18H32O CAS:56630-94-5 MolWeight:264 RetIndex:2077
CompName:6,9,12-Octadecatrien-1-ol $$ (6E,9E,12E)-6,9,12-Octadecatrien-1-ol # $$

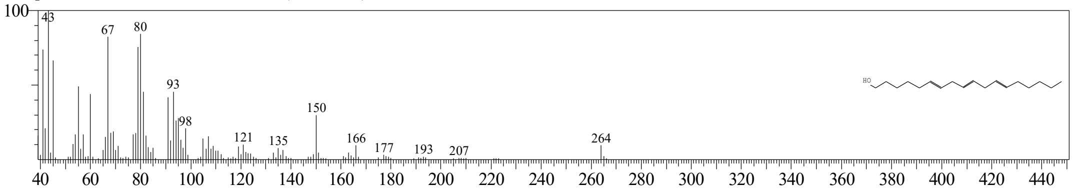

Supplement: Supplementary file 1 [file ijms-20-05913-s001.zip › ijms-580483 supplementary/ijms-580483 Supplement 1.pdf]
