# Supplementary material for: Model Optimization and In Silico Analysis of Potential Dipeptidyl Peptidase IV Antagonists from GC-MS Identified Compounds in Nauclea latifolia Leaf Extracts
Source: Int J Mol Sci. 2019 Nov 25;20(23):5913. doi: 10.3390/ijms20235913 (PMC6929178; doi:10.3390/ijms20235913)

Library

<< Target >>
Line#:1 R.Time:5.808(Scan#:38) MassPeaks:37
RawMode:Single 5.808(38) BasePeak:45.05(8413861)
BG Mode:None Group 1 - Event 1

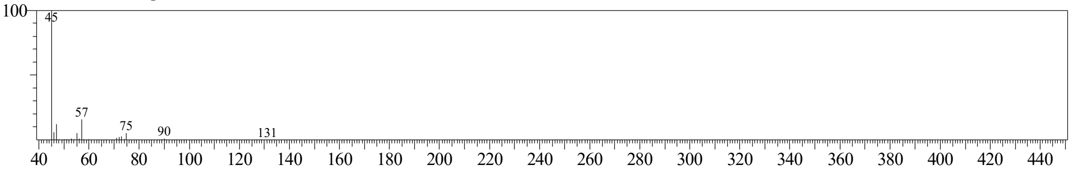


Hit#:1 Entry:1186 Library:NIST11s.lib
SI:94 Formula:C4H10O2 CAS:513-85-9 MolWeight:90 RetIndex:743
CompName:2,3-Butanediol $$ Butane-2,3-diol $$ Dimethylethylene glycol $$ 2,3-Butylene glycol $$ 2,3-Dihydroxybutane $$ D-2,3-Butane diol $$ 2,3-Butanc

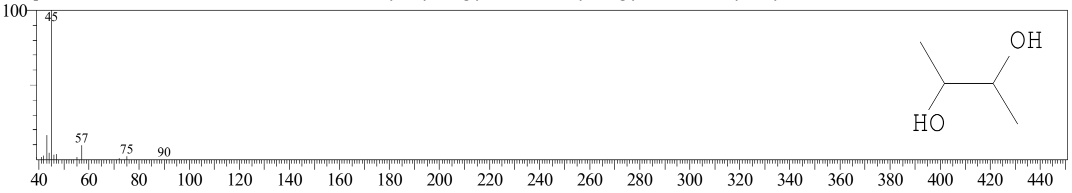


Hit#:2 Entry:1142 Library:NIST11.lib
SI:94 Formula:C4H10O2 CAS:24347-58-8 MolWeight:90 RetIndex:743
CompName:2,3-Butanediol, [R-(R*,R*)]- $$ 2,3-Butanediol # $$

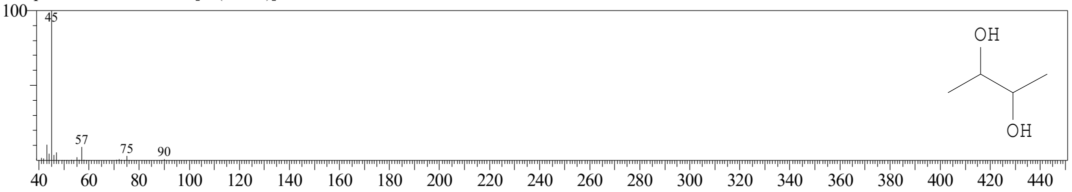


Hit#:3 Entry:1141 Library:NIST11.lib
SI:94 Formula:C4H10O2 CAS:513-85-9 MolWeight:90 RetIndex:743
CompName:2,3-Butanediol $$ Butane-2,3-diol $$ Dimethylethylene glycol $$ 2,3-Butylene glycol $$ 2,3-Dihydroxybutane $$ D-2,3-Butane diol $$ 2,3-Butanc

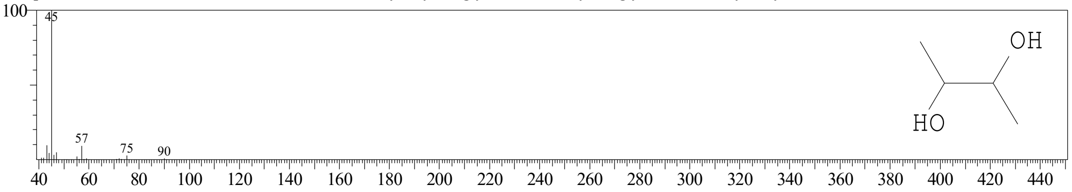


Hit#:4 Entry:1184 Library:NIST11s.lib
SI:94 Formula:C4H10O2 CAS:513-85-9 MolWeight:90 RetIndex:743
CompName:2,3-Butanediol $$ Butane-2,3-diol $$ Dimethylethylene glycol $$ 2,3-Butylene glycol $$ 2,3-Dihydroxybutane $$ D-2,3-Butane diol $$ 2,3-Butanc

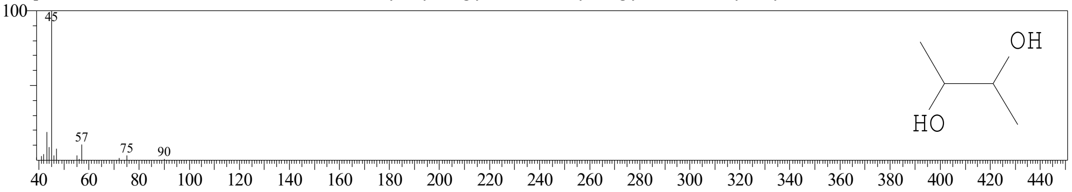


Hit#:5 Entry:1139 Library:NIST11.lib
SI:93 Formula:C4H10O2 CAS:19132-06-0 MolWeight:90 RetIndex:743
CompName:2,3-Butanediol, [S-(R*,R*)]- $$ (2S,3S)-(+)-2,3-Butanediol $$ 2,3-Butanediol # $$

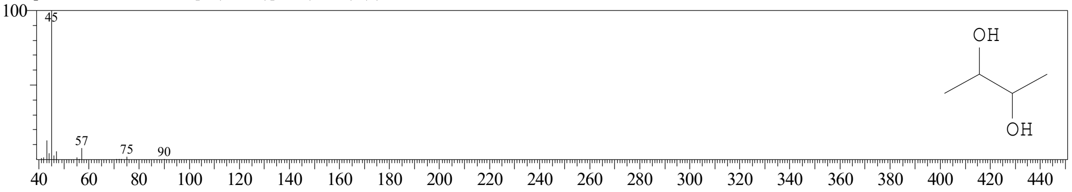

<< Target >>
Line#:2 R.Time:8.867(Scan#:405) MassPeaks:53
RawMode:Single 8.867(405) BasePeak:57.00(245537)
BG Mode:None Group 1 - Event 1

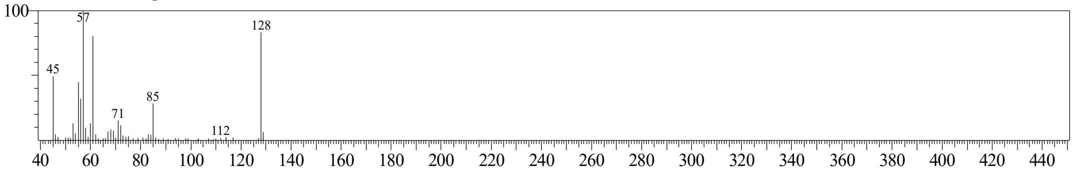


Hit#:1 Entry:7041 Library:NIST11.lib
SI:77 Formula:C6H8O3 CAS:3658-77-3 MolWeight:128 RetIndex:1022
CompName:2,5-Dimethyl-4-hydroxy-3(2H)-furanone $$ 3(2H)-Furanone, 4-hydroxy-2,5-dimethyl- $$ 3(2H)-Furanone, 2,5-dimethyl-4-hydroxy- $$ Alletone $$

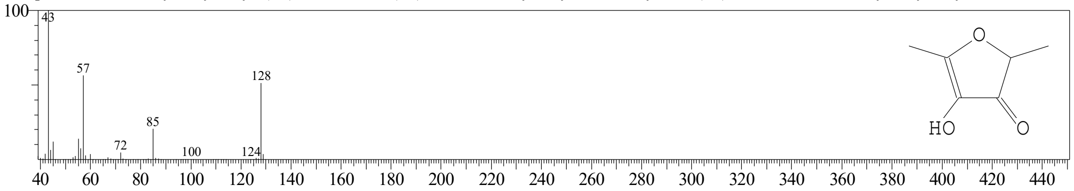


Hit#:2 Entry:7057 Library:NIST11.lib
SI:73 Formula:C6H8O3 CAS:28664-35-9 MolWeight:128 RetIndex:1088
CompName:2(5H)-Furanone, 3-hydroxy-4,5-dimethyl- $$ Sotolone $$ 4,5-Dimethyl-3-hydroxy-2(5H)-furanone $$ Furan-2(5H)-one, 3-hydroxy-4,5-dimethyl- $

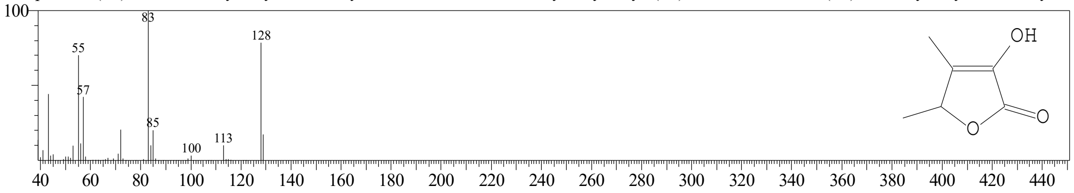


Hit#:3 Entry:17824 Library:NIST11s.lib
SI:72 Formula:C10H21NOS CAS:1114-71-2 MolWeight:203 RetIndex:1467
CompName:Pebulate $$ Carbamothioic acid, butylethyl-, S-propyl ester $$ Carbamic acid, butylethylthio-, S-propyl ester $$ Butylethylthiocarbamic acid S-pro

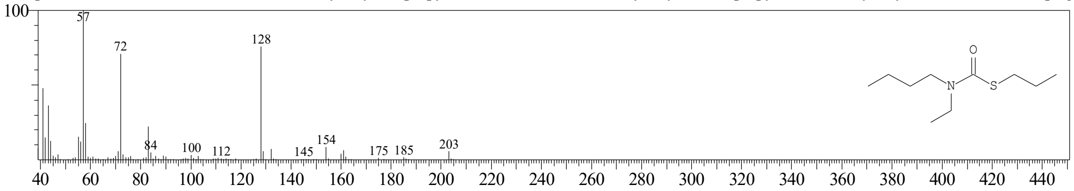


Hit#:4 Entry:86713 Library:NIST11.lib
SI:72 Formula:C9H12N2O7 CAS:957-77-7 MolWeight:260 RetIndex:2389
CompName:5-Hydroxyuridine $$ Uridine, 5-hydroxy- $$

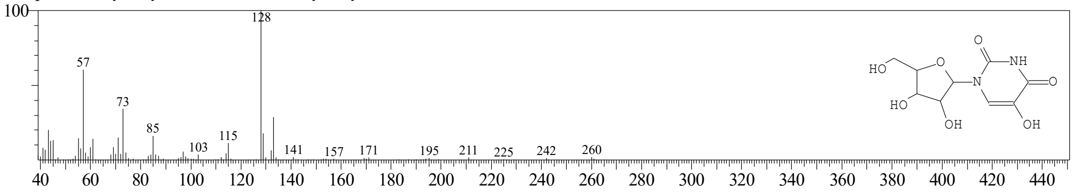


Hit#:5 Entry:74152 Library:NIST11.lib
SI:71 Formula:C11H17NOS2 CAS:147723-52-2 MolWeight:243 RetIndex:2181
CompName:Piperidine-1-dithiocarboxylic acid, 2-oxocyclopentyl ester $$ 2-Oxocyclopentyl 1-piperidinecarbodithioate # $$

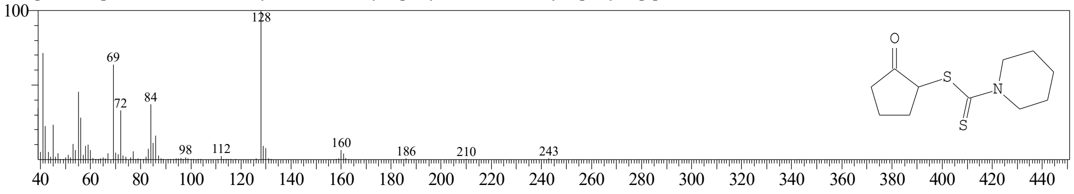

<< Target >>
Line#:3 R.Time:10.883(Scan#:647) MassPeaks:72
RawMode:Single 10.883(647) BasePeak:109.95(8362006)
BG Mode:None Group 1 - Event 1

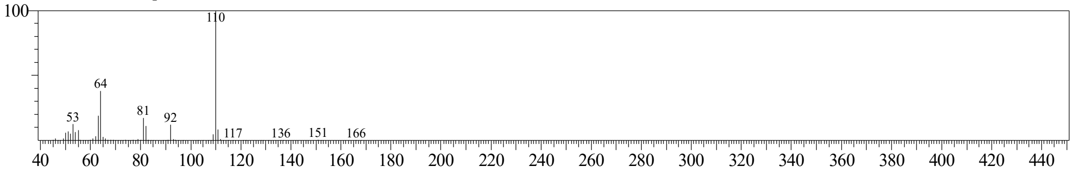


Hit#:1 Entry:2673 Library:NIST11s.lib
SI:98 Formula:C6H6O2 CAS:120-80-9 MolWeight:110 RetIndex:1122
CompName:Catechol $$ 1,2-Benzenediol $$ Pyrocatechol $$ o-Benzenediol $$ o-Dihydroxybenzene $$ o-Dioxybenzene $$ o-Hydroxyphenol $$ o-Phenylenedi

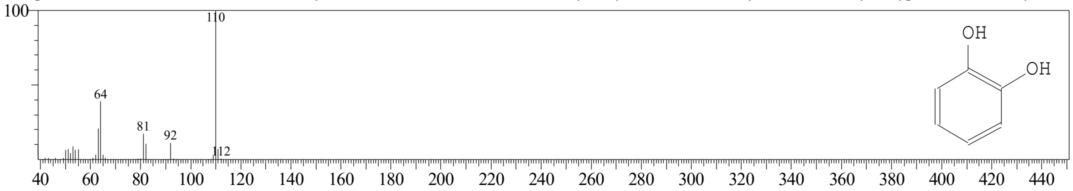


Hit#:2 Entry:2674 Library:NIST11s.lib
SI:98 Formula:C6H6O2 CAS:120-80-9 MolWeight:110 RetIndex:1122
CompName:Catechol $$ 1,2-Benzenediol $$ Pyrocatechol $$ o-Benzenediol $$ o-Dihydroxybenzene $$ o-Dioxybenzene $$ o-Hydroxyphenol $$ o-Phenylenedi

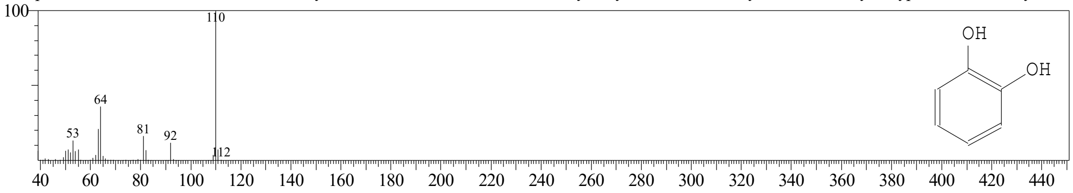


Hit#:3 Entry:3063 Library:NIST11.lib
SI:94 Formula:C6H6O2 CAS:120-80-9 MolWeight:110 RetIndex:1122
CompName:Catechol $$ 1,2-Benzenediol $$ Pyrocatechol $$ o-Benzenediol $$ o-Dihydroxybenzene $$ o-Dioxybenzene $$ o-Hydroxyphenol $$ o-Phenylenedi

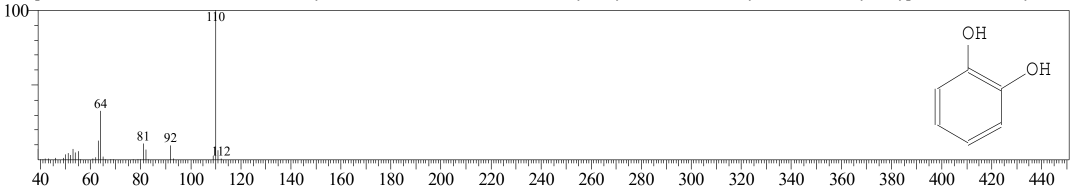


Hit#:4 Entry:2678 Library:NIST11s.lib
SI:89 Formula:C6H6O2 CAS:108-46-3 MolWeight:110 RetIndex:1122
CompName:Resorcinol $$ 1,3-Benzenediol $$ .alpha.-Resorcinol $$ m-Benzenediol $$ m-Dihydroxybenzene $$ m-Dioxybenzene $$ m-Hydroquinone $$ m-Hy

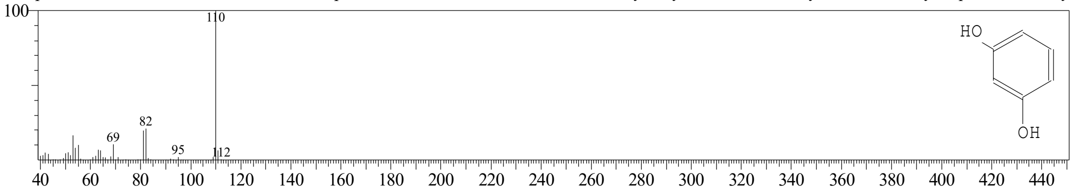


Hit#:5 Entry:2679 Library:NIST11s.lib
SI:89 Formula:C6H6O2 CAS:108-46-3 MolWeight:110 RetIndex:1122
CompName:Resorcinol $$ 1,3-Benzenediol $$ .alpha.-Resorcinol $$ m-Benzenediol $$ m-Dihydroxybenzene $$ m-Dioxybenzene $$ m-Hydroquinone $$ m-Hy

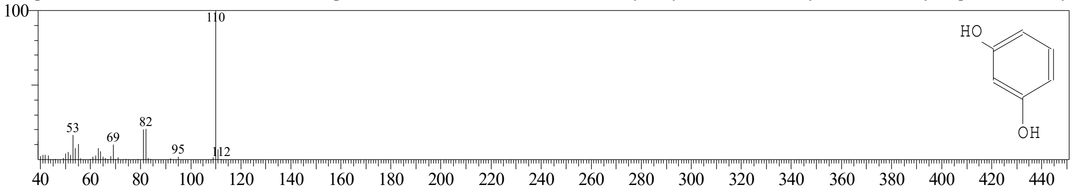

<< Target >>
Line#:4 R.Time:11.108(Scan#:674) MassPeaks:71
RawMode:Single 11.108(674) BasePeak:120.00(531607)
BG Mode:None Group 1 - Event 1

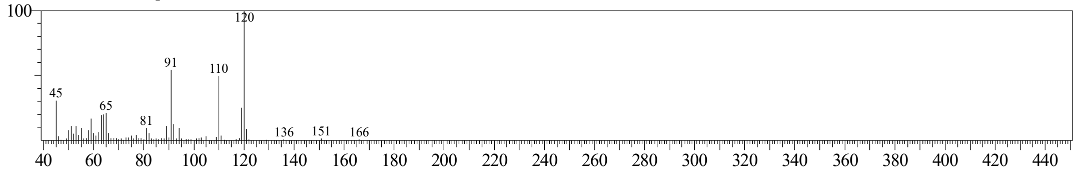


Hit#:1 Entry:4095 Library:NIST11s.lib
SI:78 Formula:C8H8O CAS:496-16-2 MolWeight:120 RetIndex:1036
CompName:Benzofuran, 2,3-dihydro- $$ Coumaran $$ Dihydrobenzofuran $$ Dihydrocoumarone $$ Kumaran $$ 2,3-Dihydrobenzofuran $$ 2,3-Dihydro-1-ben

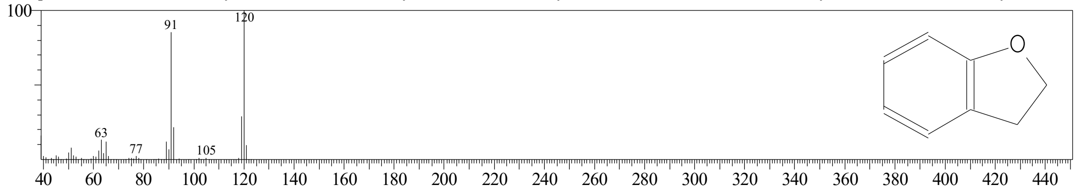


Hit#:2 Entry:5306 Library:NIST11.lib
SI:77 Formula:C8H8O CAS:496-16-2 MolWeight:120 RetIndex:1036
CompName:Benzofuran, 2,3-dihydro- $$ Coumaran $$ Dihydrobenzofuran $$ Dihydrocoumarone $$ Kumaran $$ 2,3-Dihydrobenzofuran $$ 2,3-Dihydro-1-ben

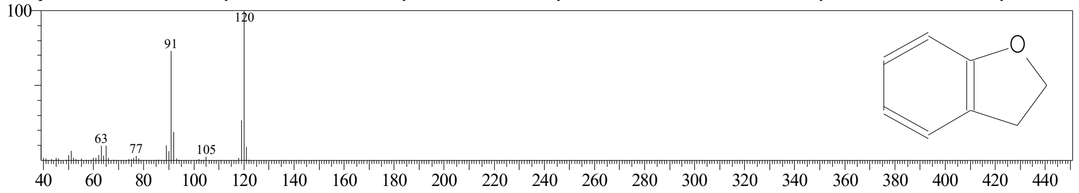


Hit#:3 Entry:5304 Library:NIST11.lib
SI:73 Formula:C8H8O CAS:766-94-9 MolWeight:120 RetIndex:959
CompName:Benzene, (ethenyloxy)- $$ Ether, phenyl vinyl $$ (Vinyloxy)benzene $$ Phenoxyethene $$ Phenoxyethylene $$ Phenyl vinyl ether $$ Vinyl phenyl e

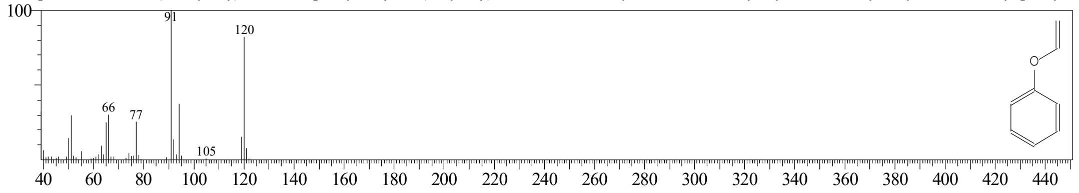


Hit#:4 Entry:4094 Library:NIST11s.lib
SI:73 Formula:C8H8O CAS:766-94-9 MolWeight:120 RetIndex:959
CompName:Benzene, (ethenyloxy)- $$ Ether, phenyl vinyl $$ (Vinyloxy)benzene $$ Phenoxyethene $$ Phenoxyethylene $$ Phenyl vinyl ether $$ Vinyl phenyl e

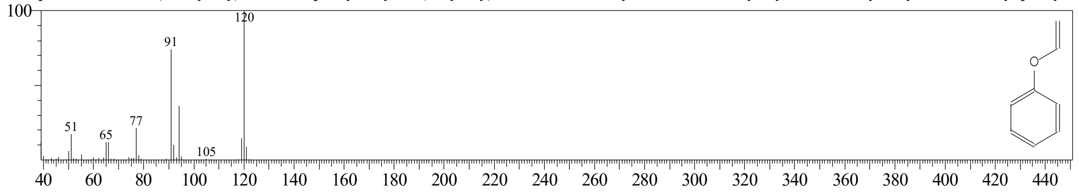


Hit#:5 Entry:14596 Library:NIST11.lib
SI:72 Formula:C10H12O CAS:5459-40-5 MolWeight:148 RetIndex:1172
CompName:4-Ethoxystyrene $$ p-Ethoxystyrene $$ Benzene, 1-ethenyl-4-ethoxy- $$ 1-Ethoxy-4-vinylbenzene # $$ p-Vinylphenetole $$

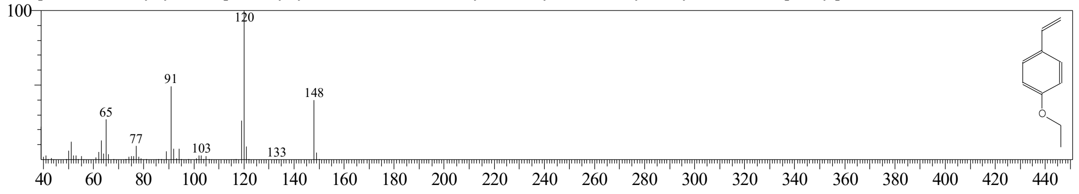

<< Target >>
Line#:5 R.Time:12.342(Scan#:822) MassPeaks:88
RawMode:Single 12.342(822) BasePeak:150.05(795392)
BG Mode:None Group 1 - Event 1

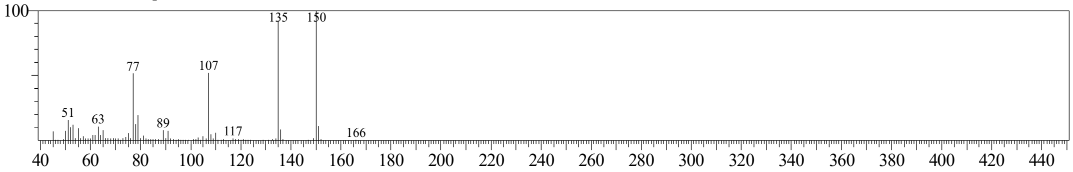


Hit#:1 Entry:15257 Library:NIST11.lib
SI:91 Formula:C9H10O2 CAS:7786-61-0 MolWeight:150 RetIndex:1293
CompName:2-Methoxy-4-vinylphenol $$ Phenol, 4-ethenyl-2-methoxy- $$ Phenol, 2-methoxy-4-vinyl- $$ 4-Hydroxy-3-methoxystyrene $$ p-Vinylguaiacol $$ 4

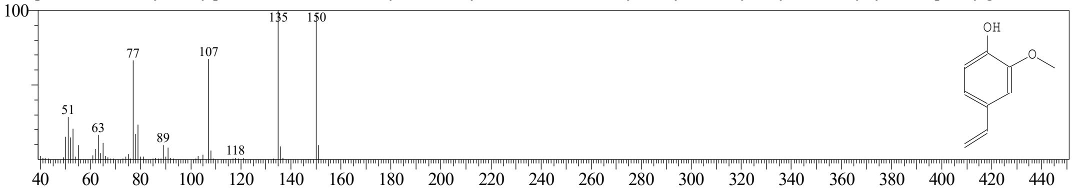


Hit#:2 Entry:8939 Library:NIST11s.lib
SI:82 Formula:C9H10O2 CAS:586-37-8 MolWeight:150 RetIndex:1218
CompName:3-Methoxyacetophenone $$ Ethanone, 1-(3-methoxyphenyl)- $$ Acetophenone, 3'-methoxy- $$ 3-Acetylanisole $$ 1-Acetyl-3-methoxybenzene $$ r

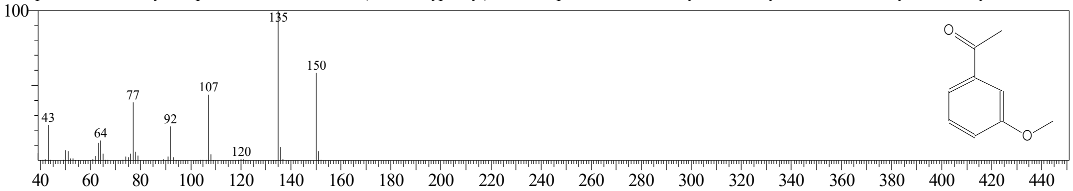


Hit#:3 Entry:15258 Library:NIST11.lib
SI:82 Formula:C9H10O2 CAS:586-37-8 MolWeight:150 RetIndex:1218
CompName:3-Methoxyacetophenone $$ Ethanone, 1-(3-methoxyphenyl)- $$ Acetophenone, 3'-methoxy- $$ 3-Acetylanisole $$ 1-Acetyl-3-methoxybenzene $$ r

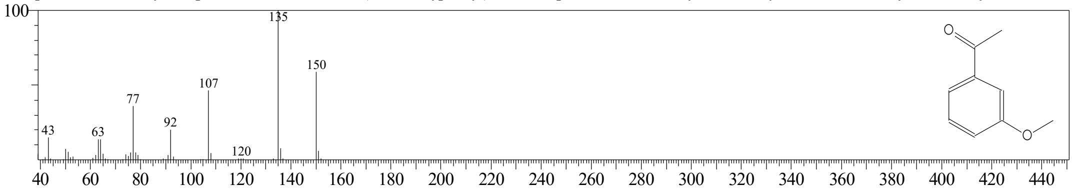


Hit#:4 Entry:8932 Library:NIST11s.lib
SI:82 Formula:C9H10O2 CAS:875-59-2 MolWeight:150 RetIndex:1363
CompName:4-Hydroxy-2-methylacetophenone $$ 2-Methyl-4-hydroxyacetophenone $$ Ethanone, 1-(4-hydroxy-2-methylphenyl)- $$ 1-(4-Hydroxy-2-methylphe

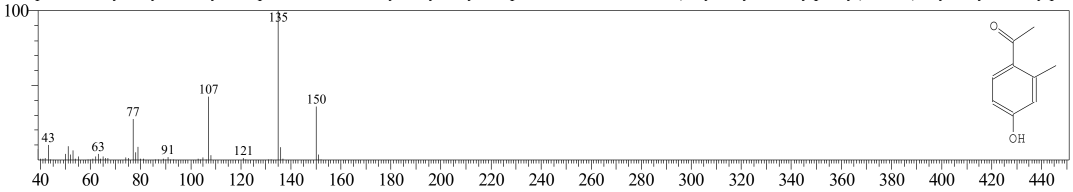


Hit#:5 Entry:8936 Library:NIST11s.lib
SI:82 Formula:C9H10O2 CAS:876-02-8 MolWeight:150 RetIndex:1363
CompName:4-Hydroxy-3-methylacetophenone $$ 4'-Hydroxy-3'-methylacetophenone $$ Ethanone, 1-(4-hydroxy-3-methylphenyl)- $$ 1-(4-Hydroxy-3-methylph

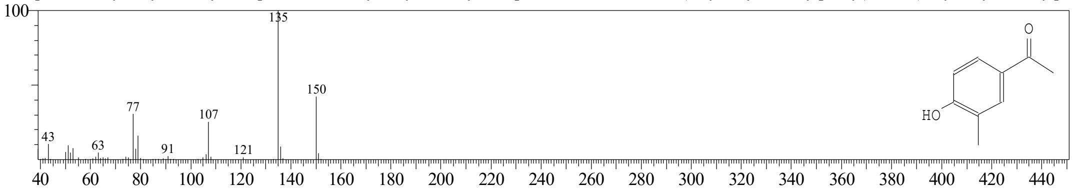

<< Target >>
Line#:6 R.Time:12.917(Scan#:891) MassPeaks:93
RawMode:Single 12.917(891) BasePeak:71.00(146338)
BG Mode:None Group 1 - Event 1

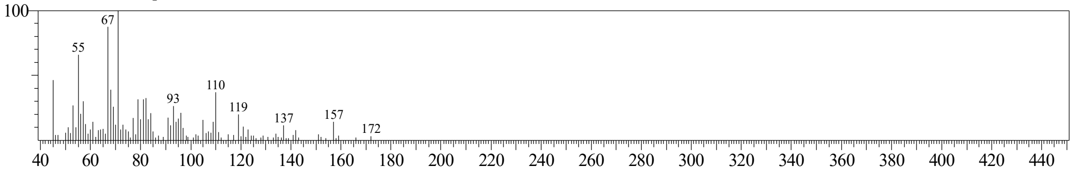


Hit#:1 Entry:12731 Library:NIST11s.lib
SI:87 Formula:C10H18O2 CAS:64142-78-5 MolWeight:170 RetIndex:1325
CompName:2,7-Octadiene-1,6-diol, 2,6-dimethyl- $$ 8-Hydroxylinalool $$ 2,6-Dimethyl-2,7-octadiene-1,6-diol $$

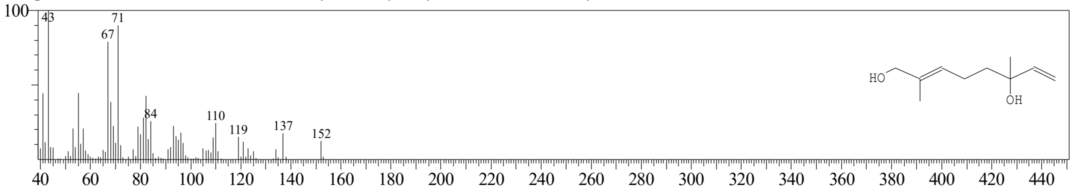


Hit#:2 Entry:25783 Library:NIST11.lib
SI:79 Formula:C10H18O2 CAS:26489-17-8 MolWeight:170 RetIndex:1471
CompName:2,6-Octadiene-1,8-diol, 2,6-dimethyl- $$ (2Z,6Z)-2,6-Dimethyl-2,6-octadiene-1,8-diol # $$

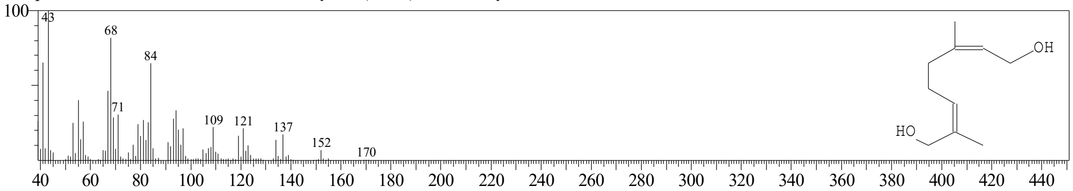


Hit#:3 Entry:82697 Library:NIST11.lib
SI:79 Formula:C16H30O2 CAS:0-00-0 MolWeight:254 RetIndex:1792
CompName:1,2-15,16-Diepoxyhexadecane $$ 2-[12-(2-Oxiranyl)dodecyl]oxirane # $$

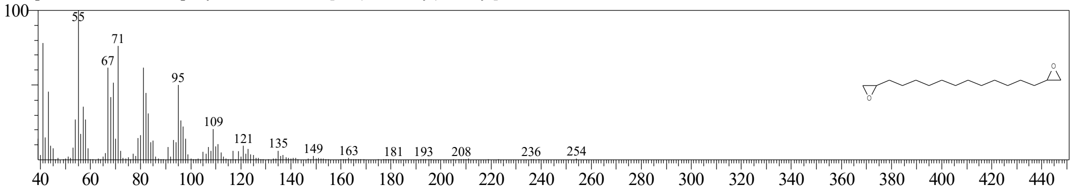


Hit#:4 Entry:32923 Library:NIST11.lib
SI:78 Formula:C12H22O CAS:72845-33-1 MolWeight:182 RetIndex:1181
CompName:1,6-Octadiene, 3-ethoxy-3,7-dimethyl- $$ 3-Ethoxy-3,7-dimethyl-1,6-octadiene $$ Ethyl linalool $$

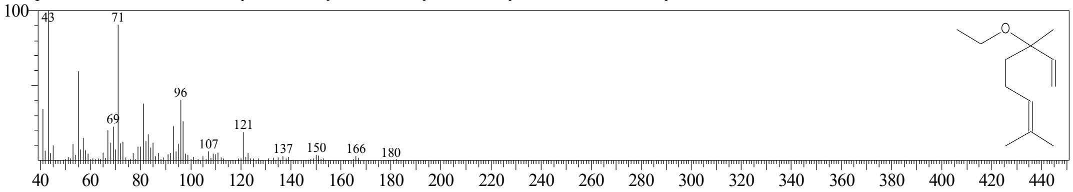


Hit#:5 Entry:49565 Library:NIST11.lib
SI:77 Formula:C14H24O CAS:0-00-0 MolWeight:208 RetIndex:1663
CompName:13-Tetradece-11-yn-1-ol $$ 13-Tetradecen-11-yn-1-ol # $$

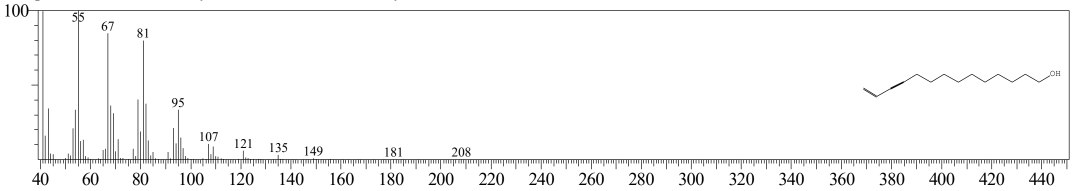

<< Target >>
Line#:7 R.Time:13.900(Scan#:1009) MassPeaks:106
RawMode:Single 13.900(1009) BasePeak:136.00(604937)
BG Mode:None Group 1 - Event 1

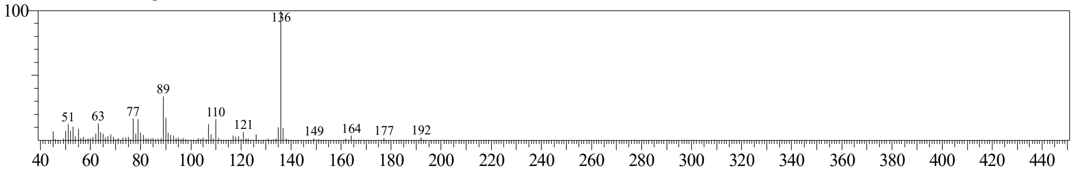


Hit#:1 Entry:11540 Library:NIST11s.lib
SI:79 Formula:C9H8O3 CAS:7310-95-4 MolWeight:164 RetIndex:1617
CompName:2-Hydroxy-5-methylisophthalaldehyde $$ 1,3-Benzenedicarboxaldehyde, 2-hydroxy-5-methyl- $$

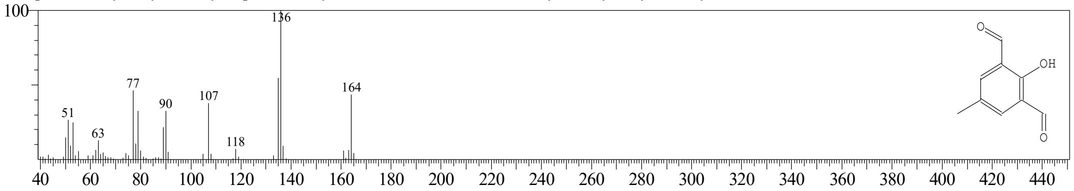


Hit#:2 Entry:22111 Library:NIST11.lib
SI:78 Formula:C9H8O3 CAS:7310-95-4 MolWeight:164 RetIndex:1617
CompName:2-Hydroxy-5-methylisophthalaldehyde $$ 1,3-Benzenedicarboxaldehyde, 2-hydroxy-5-methyl- $$

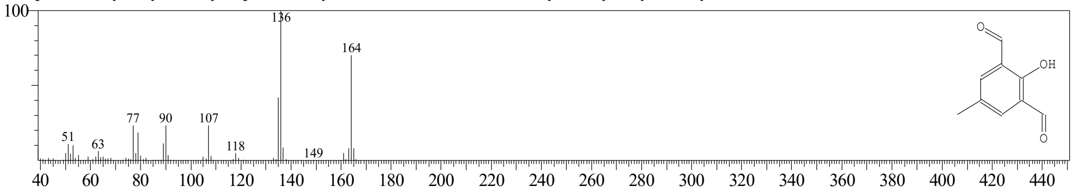


Hit#:3 Entry:56489 Library:NIST11.lib
SI:77 Formula:C9H9N5O2 CAS:339302-07-7 MolWeight:219 RetIndex:0
CompName:Phenol, 2-methoxy-4-(tetrazol-1-yliminomethyl)- $$ 2-Methoxy-4-[(E)-(1H-tetraazol-1-ylimino)methyl]phenol # $$

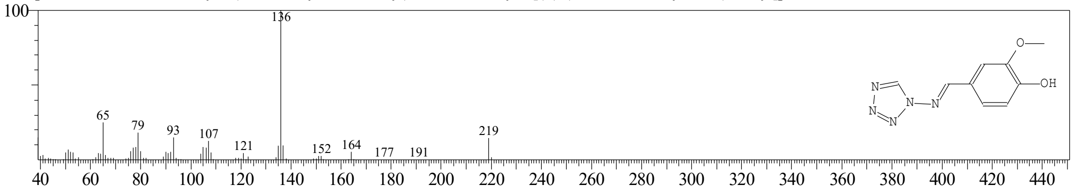


Hit#:4 Entry:22854 Library:NIST11.lib
SI:77 Formula:C10H15NO CAS:39590-27-7 MolWeight:165 RetIndex:1425
CompName:2-Ethoxyphenethylamine $$ 2-(2-Ethoxyphenyl)ethanamine # $$

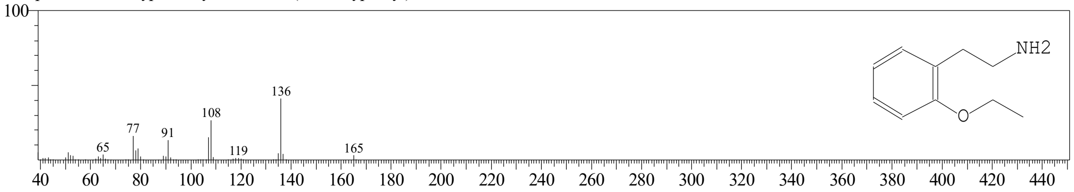


Hit#:5 Entry:9536 Library:NIST11.lib
SI:75 Formula:C8H8O2 CAS:90111-15-2 MolWeight:136 RetIndex:1316
CompName:3-Hydroxy-2-methylbenzaldehyde

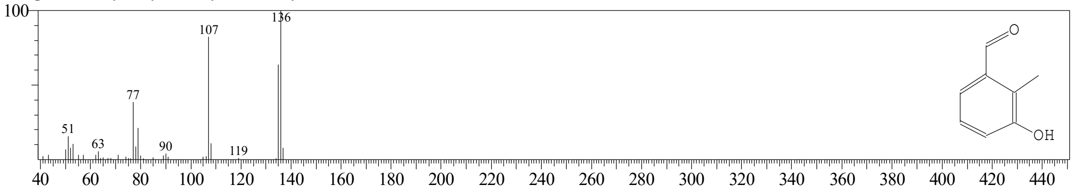

<< Target >>
Line#:8 R.Time:14.767(Scan#:1113) MassPeaks:118
RawMode:Single 14.767(1113) BasePeak:151.00(200178)
BG Mode:None Group 1 - Event 1

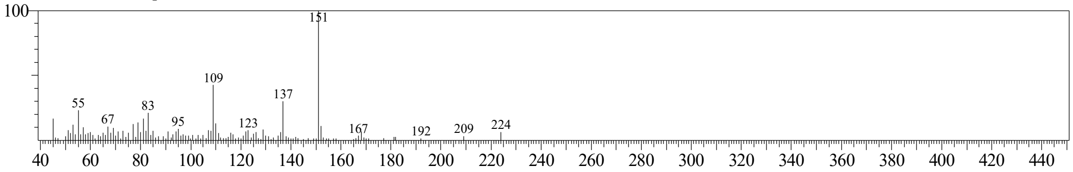


Hit#:1 Entry:64618 Library:NIST11.lib
SI:72 Formula:C10H15BrO CAS:64161-50-8 MolWeight:230 RetIndex:1417
CompName:Bicyclo[2.2.1]heptan-2-one, 1-(bromomethyl)-7,7-dimethyl-, (1S)- $$ 1-(Bromomethyl)-7,7-dimethylbicyclo[2.2.1]heptan-2-one # $$

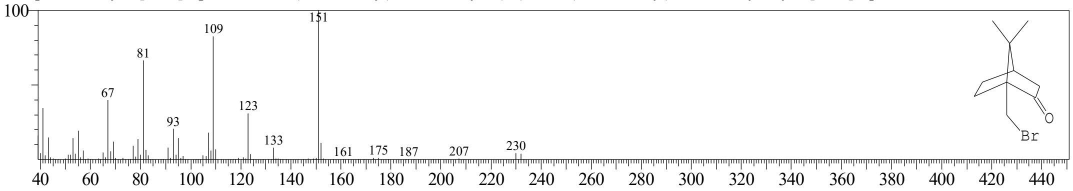


Hit#:2 Entry:62318 Library:NIST11.lib
SI:72 Formula:C14H26O2 CAS:126-86-3 MolWeight:226 RetIndex:1485
CompName:2,4,7,9-Tetramethyl-5-decyn-4,7-diol $$ 2,4,7,9-Tetramethyl-5-decyne-4,7-diol $$ 5-Decyne-4,7-diol, 2,4,7,9-tetramethyl- $$ Surfynol 104 $$ Surfy

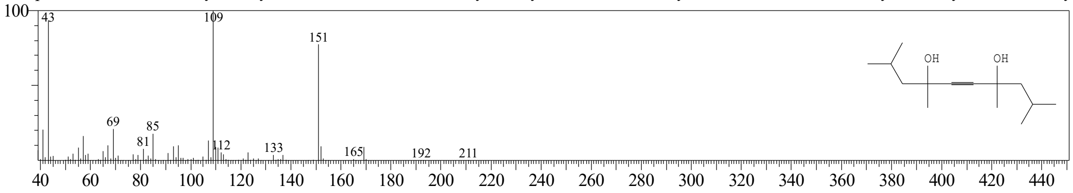


Hit#:3 Entry:31663 Library:NIST11.lib
SI:71 Formula:C12H20O CAS:62338-24-3 MolWeight:180 RetIndex:1317
CompName:Ethanone, 1-(4,5-diethyl-2-methyl-1-cyclopenten-1-yl)- $$ 1-(4,5-Diethyl-2-methyl-1-cyclopenten-1-yl)ethanone # $$

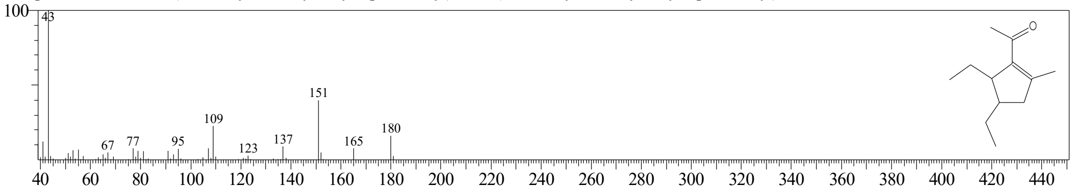


Hit#:4 Entry:40152 Library:NIST11.lib
SI:71 Formula:C12H18O2 CAS:0-00-0 MolWeight:194 RetIndex:1323
CompName:6-(3,3-Dimethyl-oxiran-2-ylidene)-5,5-dimethyl-hex-3-en-2-one $$ (3E,6Z)-6-(3,3-Dimethyl-2-oxiranylidene)-5,5-dimethyl-3-hexen-2-one # $$

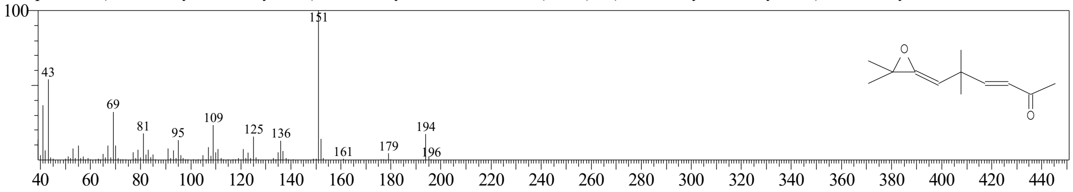


Hit#:5 Entry:20700 Library:NIST11s.lib
SI:71 Formula:C14H26O2 CAS:126-86-3 MolWeight:226 RetIndex:1485
CompName:2,4,7,9-Tetramethyl-5-decyn-4,7-diol $$ 2,4,7,9-Tetramethyl-5-decyne-4,7-diol $$ 5-Decyne-4,7-diol, 2,4,7,9-tetramethyl- $$ Surfynol 104 $$ Surfy

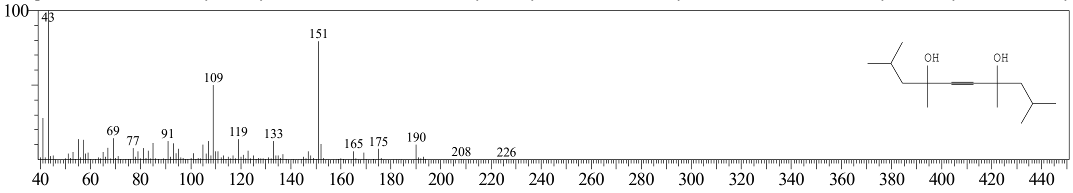

<< Target >>
Line#:9 R.Time:14.983(Scan#:1139) MassPeaks:126
RawMode:Single 14.983(1139) BasePeak:180.00(227812)
BG Mode:None Group 1 - Event 1

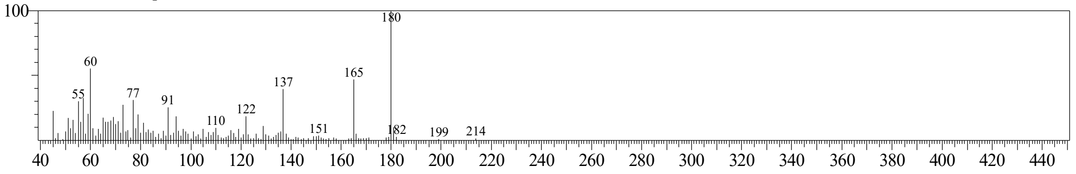


Hit#:1 Entry:81228 Library:NIST11.lib
SI:65 Formula:C16H28O2 CAS:459-67-6 MolWeight:252 RetIndex:1993
CompName:11-(2-Cyclopenten-1-yl)undecanoic acid, (+)- $$ Hydnocarpic acid $$ 2-Cyclopentene-1-undecanoic acid $$ Isolated from chaulmoogra oil $$ 11-(2

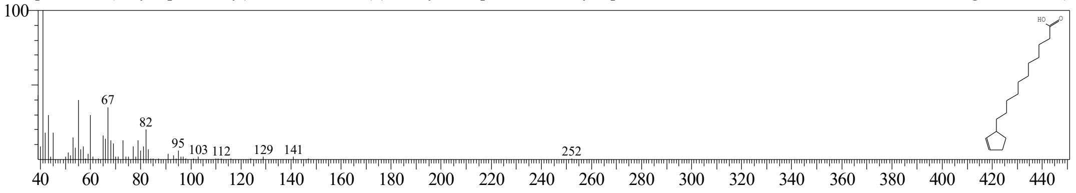


Hit#:2 Entry:31435 Library:NIST11.lib
SI:64 Formula:C10H12O3 CAS:54644-27-8 MolWeight:180 RetIndex:1580
CompName:1,2,4-Cyclopentanetrione, 3-(2-pentenyl)- $$ 3-[(2E)-2-Pentenyl]-1,2,4-cyclopentanetrione # $$

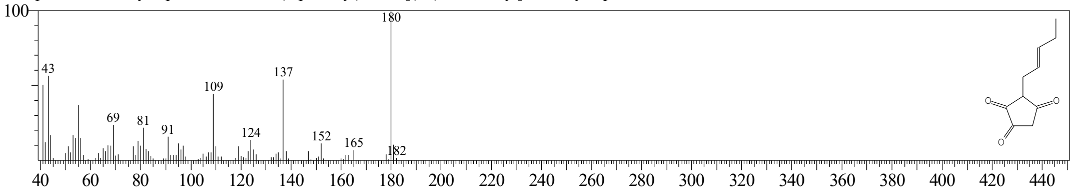


Hit#:3 Entry:14404 Library:NIST11s.lib
SI:64 Formula:C10H12O3 CAS:54644-27-8 MolWeight:180 RetIndex:1580
CompName:1,2,4-Cyclopentanetrione, 3-(2-pentenyl)- $$ 3-[(2E)-2-Pentenyl]-1,2,4-cyclopentanetrione # $$

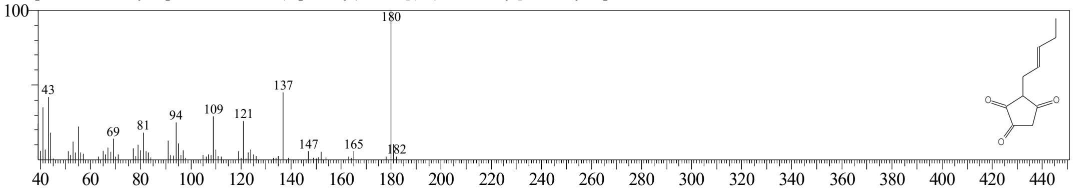


Hit#:4 Entry:31655 Library:NIST11.lib
SI:64 Formula:C12H20O CAS:77822-49-2 MolWeight:180 RetIndex:1157
CompName:Furan, 2,3-dihydro-2,2-dimethyl-3-(1-methylethenyl)-5-(1-methylethyl)- $$ 3-Isopropenyl-5-isopropyl-2,2-dimethyl-2,3-dihydrofuran # $$

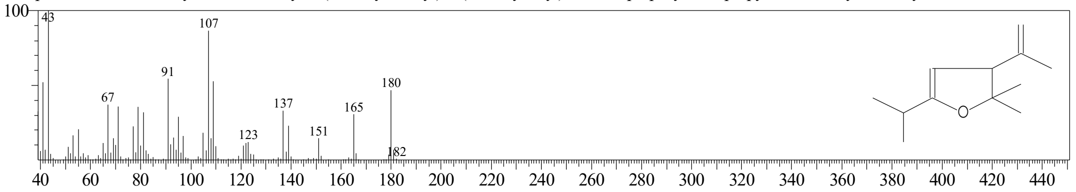


Hit#:5 Entry:31479 Library:NIST11.lib
SI:64 Formula:C11H16O2 CAS:15051-75-9 MolWeight:180 RetIndex:1345
CompName:3-Hydroxymethylene-1,7,7-trimethylbicyclo[2.2.1]heptan-2-one $$ (3E)-3-(Hydroxymethylene)-1,7,7-trimethylbicyclo[2.2.1]heptan-2-one # $$

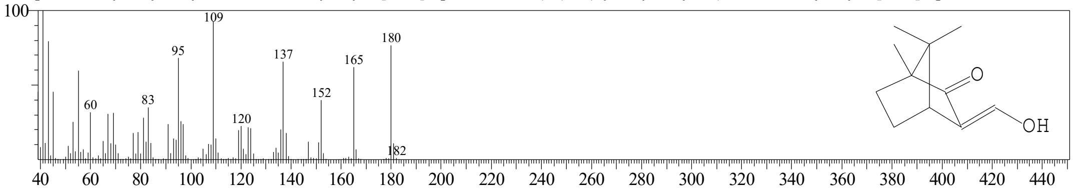

<< Target >>
Line#:10 R.Time:15.400(Scan#:1189) MassPeaks:125
RawMode:Single 15.400(1189) BasePeak:55.00(769118)
BG Mode:None Group 1 - Event 1

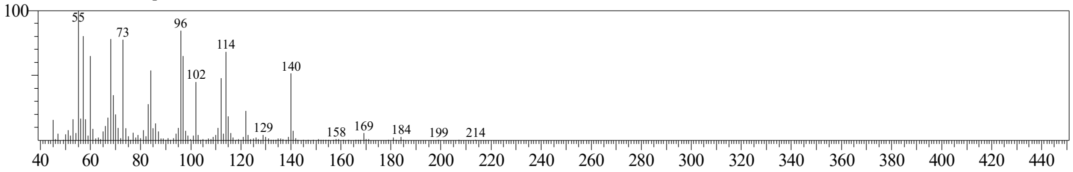


Hit#:1 Entry:19273 Library:NIST11.lib
SI:75 Formula:C8H14O3 CAS:15458-61-4 MolWeight:158 RetIndex:1347
CompName:9-Oxabicyclo[3.3.1]nonane-2,6-diol

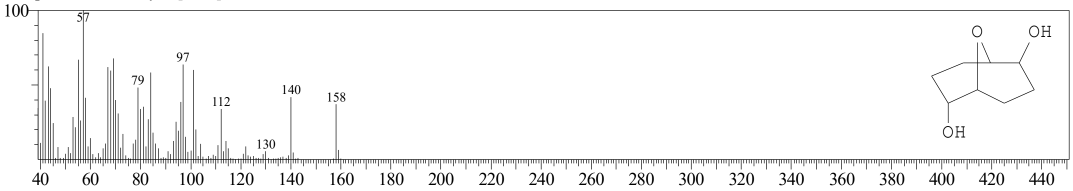


Hit#:2 Entry:4003 Library:NIST11.lib
SI:74 Formula:C6H10O2 CAS:0-00-0 MolWeight:114 RetIndex:982
CompName:cis-3-Hexenoic acid $$ (3Z)-3-Hexenoic acid # $$

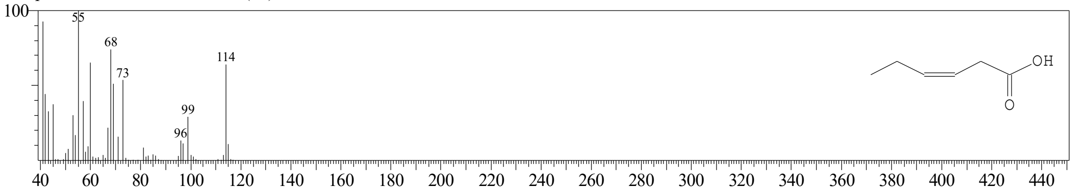


Hit#:3 Entry:33864 Library:NIST11.lib
SI:73 Formula:C11H20O2 CAS:4189-02-0 MolWeight:184 RetIndex:1479
CompName:2-Undecenoic acid $$ Undec-2-enoic acid $$

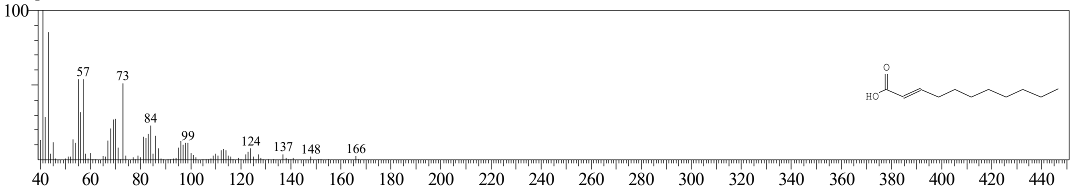


Hit#:4 Entry:11110 Library:NIST11.lib
SI:73 Formula:C8H12O2 CAS:19740-79-5 MolWeight:140 RetIndex:1142
CompName:9-Oxabicyclo[3.3.1]nonan-2-one

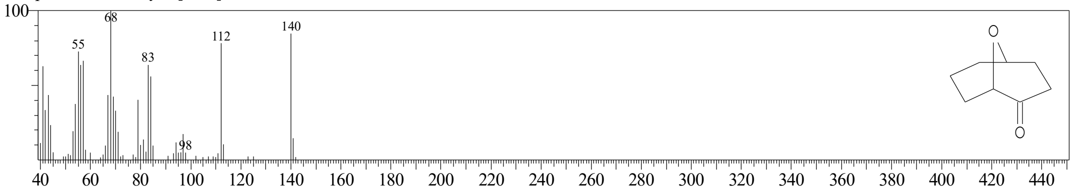


Hit#:5 Entry:33865 Library:NIST11.lib
SI:72 Formula:C11H20O2 CAS:15790-94-0 MolWeight:184 RetIndex:1479
CompName:trans-2-undecenoic acid $$ (2E)-2-Undecenoic acid # $$

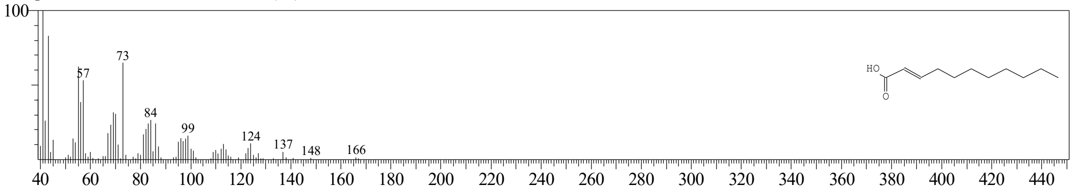

<< Target >>
Line#:11 R.Time:15.800(Scan#:1237) MassPeaks:136
RawMode:Single 15.800(1237) BasePeak:108.00(1405748)
BG Mode:None Group 1 - Event 1

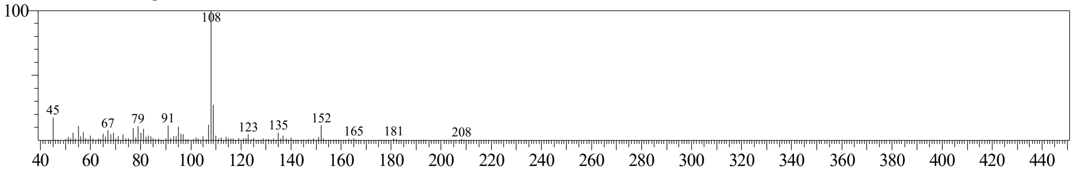


Hit#:1 Entry:49452 Library:NIST11.lib
SI:87 Formula:C13H20O2 CAS:52210-15-8 MolWeight:208 RetIndex:1627
CompName:2-Cyclohexen-1-one, 4-(3-hydroxy-1-butenyl)-3,5,5-trimethyl-, [R-[R*,R*-(E)]]- $$ 2-Cyclohexen-1-one, 4-[(1E,3R)-3-hydroxy-1-buten-1-yl]-3,5,

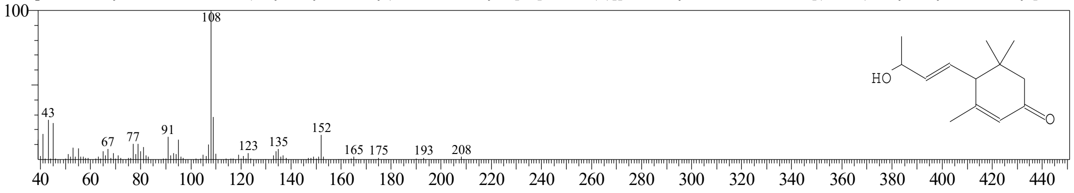


Hit#:2 Entry:49451 Library:NIST11.lib
SI:86 Formula:C13H20O2 CAS:34318-21-3 MolWeight:208 RetIndex:1627
CompName:2-Cyclohexen-1-one, 4-(3-hydroxy-1-butenyl)-3,5,5-trimethyl- $$ 3-Oxo-.alpha.-ionol $$ 4-(3-Hydroxy-1-butenyl)-3,5,5-trimethyl-2-cyclohexen-1-

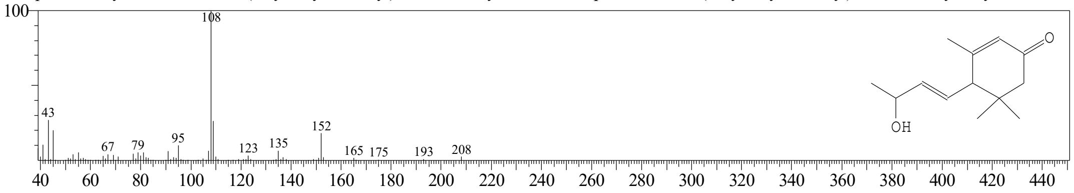


Hit#:3 Entry:9560 Library:NIST11s.lib
SI:81 Formula:C10H16O CAS:4501-58-0 MolWeight:152 RetIndex:1155
CompName:.alpha.-Campholenal $$ (R)-.alpha.-Campholene aldehyde $$ 3-Cyclopentene-1-acetaldehyde, 2,2,3-trimethyl- $$ .alpha.-Compholene aldehyde $$

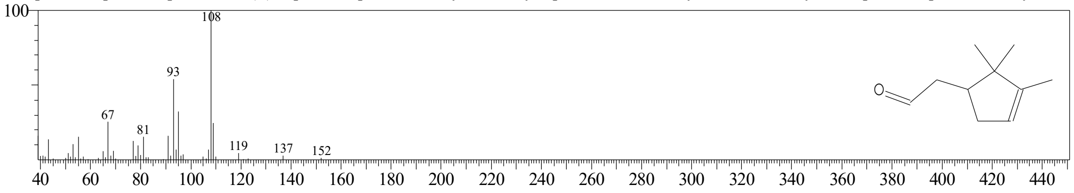


Hit#:4 Entry:16472 Library:NIST11.lib
SI:80 Formula:C10H16O CAS:4501-58-0 MolWeight:152 RetIndex:1155
CompName:.alpha.-Campholenal $$ (R)-.alpha.-Campholene aldehyde $$ 3-Cyclopentene-1-acetaldehyde, 2,2,3-trimethyl- $$ .alpha.-Compholene aldehyde $$

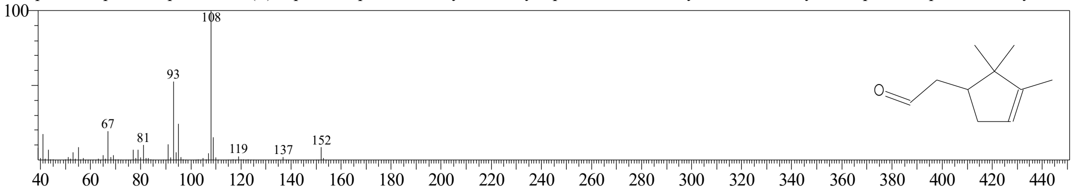


Hit#:5 Entry:9561 Library:NIST11s.lib
SI:78 Formula:C10H16O CAS:4501-58-0 MolWeight:152 RetIndex:1155
CompName:.alpha.-Campholenal $$ (R)-.alpha.-Campholene aldehyde $$ 3-Cyclopentene-1-acetaldehyde, 2,2,3-trimethyl- $$ .alpha.-Compholene aldehyde $$

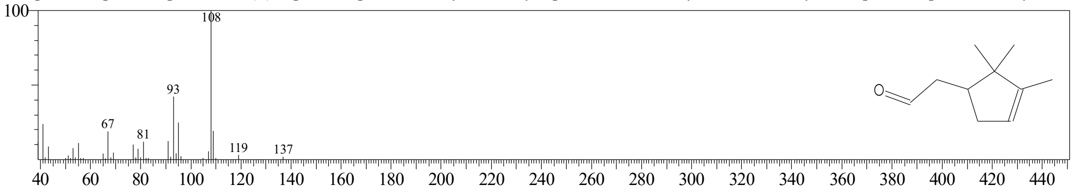

<< Target >>
Line#:12 R.Time:16.050(Scan#:1267) MassPeaks:137
RawMode:Single 16.050(1267) BasePeak:60.00(317902)
BG Mode:None Group 1 - Event 1

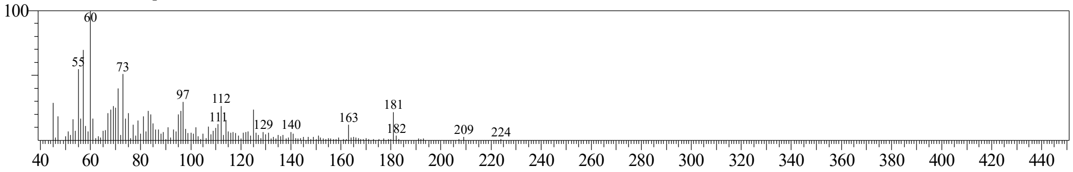


Hit#:1 Entry:51953 Library:NIST11.lib
SI:75 Formula:C11H16O4 CAS:117132-08-8 MolWeight:212 RetIndex:1610
CompName:9,9-Dimethoxybicyclo[3.3.1]nona-2,4-dione $$ 9,9-Dimethoxybicyclo[3.3.1]nonane-2,4-dione # $$

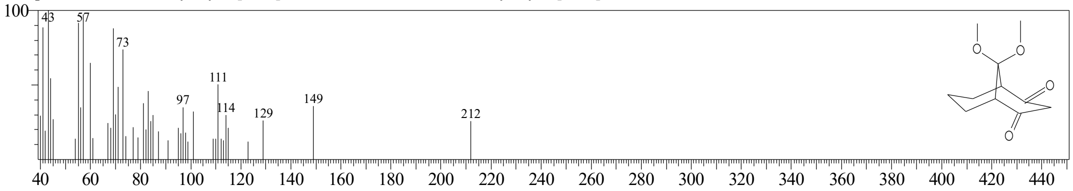


Hit#:2 Entry:25037 Library:NIST11s.lib
SI:73 Formula:C18H34O2 CAS:112-80-1 MolWeight:282 RetIndex:2175
CompName:Oleic Acid $$ 9-Octadecenoic acid (Z)- $$ .DELTA.9-cis-Oleic acid $$ cis-Oleic Acid $$ cis-9-Octadecenoic Acid $$ Emersol 211 $$ Emersol 220 \

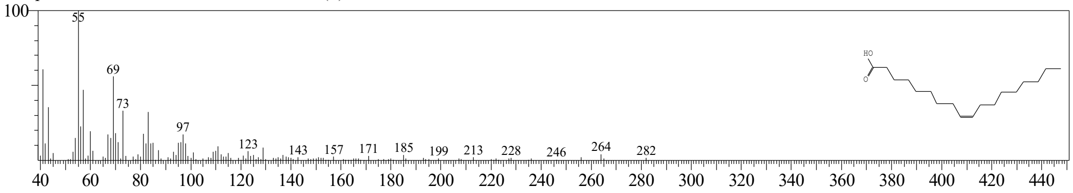


Hit#:3 Entry:205078 Library:NIST11.lib
SI:73 Formula:C18H32O16 CAS:597-12-6 MolWeight:504 RetIndex:4506
CompName:.alpha.-D-Glucopyranoside, O-.alpha.-D-glucopyranosyl-(1.fwdarw.3)-.beta.-D-fructofuranosyl $$ Glucopyranoside, O-.alpha.-D-glucopyranosyl-(1

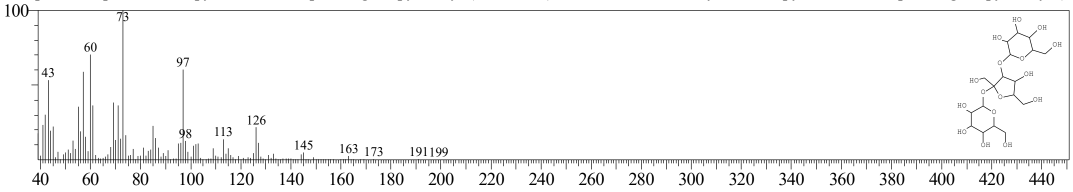


Hit#:4 Entry:84362 Library:NIST11.lib
SI:72 Formula:C16H32O2 CAS:57-10-3 MolWeight:256 RetIndex:1968
CompName:n-Hexadecanoic acid $$ Hexadecanoic acid $$ n-Hexadecoic acid $$ Palmitic acid $$ Pentadecanecarboxylic acid $$ 1-Pentadecanecarboxylic acid

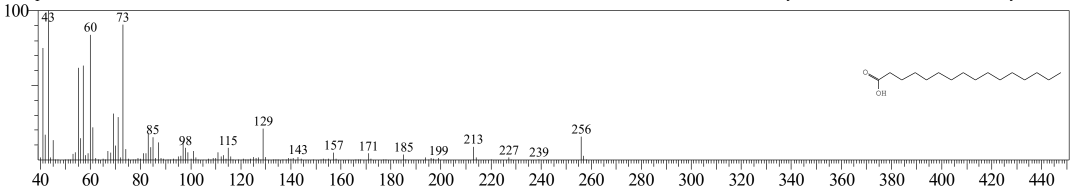


Hit#:5 Entry:10618 Library:NIST11s.lib
SI:72 Formula:C9H18O2 CAS:112-05-0 MolWeight:158 RetIndex:1272
CompName:Nonanoic acid $$ n-Nonanoic acid $$ n-Nonoic acid $$ n-Nonylic acid $$ Nonoic acid $$ Nonylic acid $$ Pelargic acid $$ Pelargonic acid $$ 1-Oc

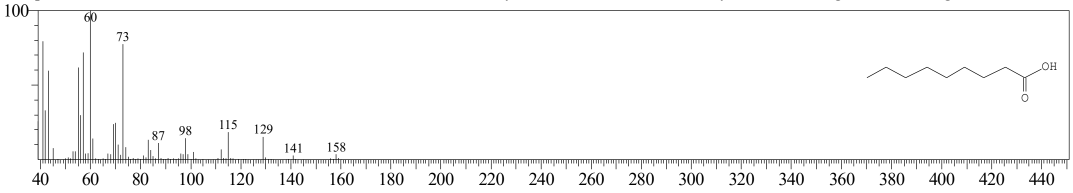

<< Target >>
Line#:13 R.Time:16.300(Scan#:1297) MassPeaks:134
RawMode:Single 16.300(1297) BasePeak:93.00(198199)
BG Mode:None Group 1 - Event 1

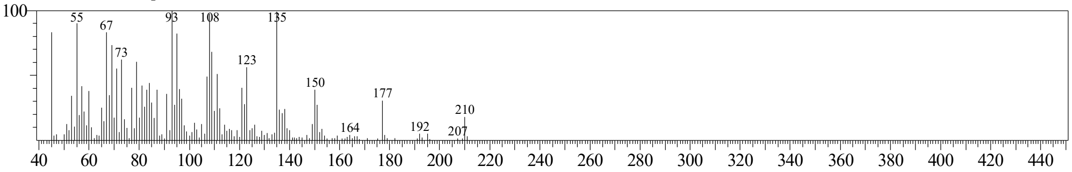


Hit#:1 Entry:50842 Library:NIST11.lib
SI:87 Formula:C13H22O2 CAS:36151-02-7 MolWeight:210 RetIndex:1619
CompName:2-Cyclohexen-1-one, 4-(3-hydroxybutyl)-3,5,5-trimethyl- $$ 3-Oxo-7,8-dihydro-.alpha.-ionol $$ Blumenol C $$ 4-(3-Hydroxybutyl)-3,5,5-trimethy

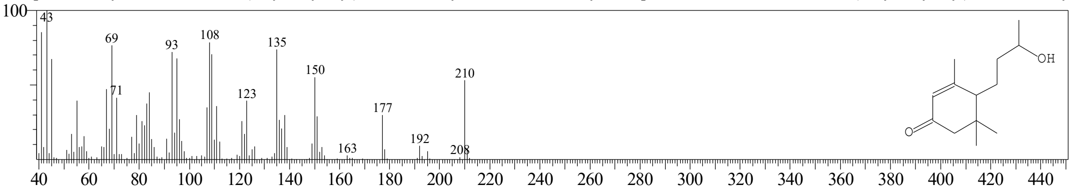


Hit#:2 Entry:48074 Library:NIST11.lib
SI:73 Formula:C15H26 CAS:62337-96-6 MolWeight:206 RetIndex:1369
CompName:Cyclohexane, 1,1,2-trimethyl-3,5-bis(1-methylethenyl)-, (2.alpha.,3.alpha.,5.beta.)- $$ 3,5-Diisopropenyl-1,1,2-trimethylcyclohexane, (2.alpha.,3.al

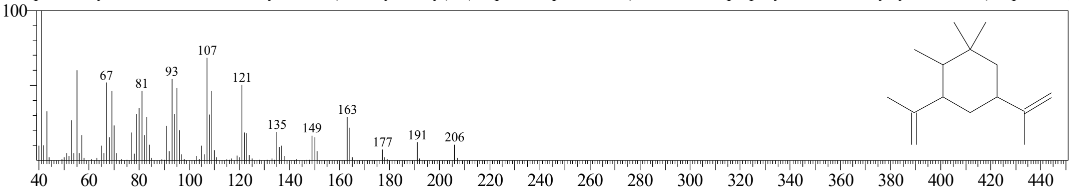


Hit#:3 Entry:24813 Library:NIST11.lib
SI:73 Formula:C11H20O CAS:0-00-0 MolWeight:168 RetIndex:1459
CompName:6-Methyl-cyclodec-5-enol $$ 6-Methyl-5-cyclodecen-1-ol # $$

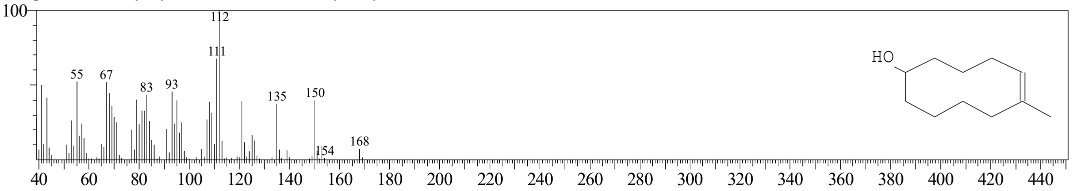


Hit#:4 Entry:24762 Library:NIST11.lib
SI:73 Formula:C11H20O CAS:54972-52-0 MolWeight:168 RetIndex:1363
CompName:1-Naphthalenol, decahydro-4a-methyl- $$ 4a-Methyldecahydro-1-naphthalenol # $$

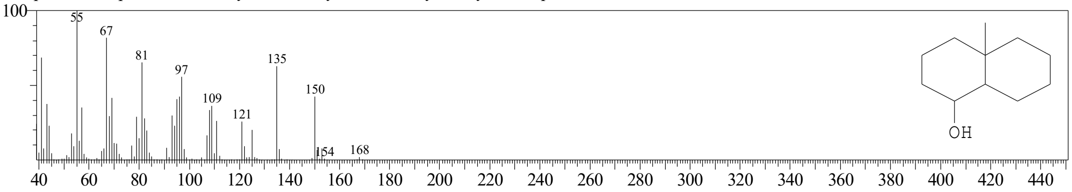


Hit#:5 Entry:24748 Library:NIST11.lib
SI:73 Formula:C11H20O CAS:78996-11-9 MolWeight:168 RetIndex:1266
CompName:(2,2,6-Trimethyl-bicyclo[4.1.0]hept-1-yl)-methanol

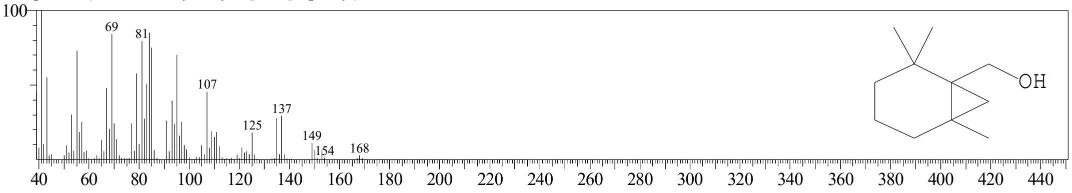

<< Target >>
Line#:14 R.Time:17.067(Scan#:1389) MassPeaks:149
RawMode:Single 17.067(1389) BasePeak:124.00(3408422)
BG Mode:None Group 1 - Event 1

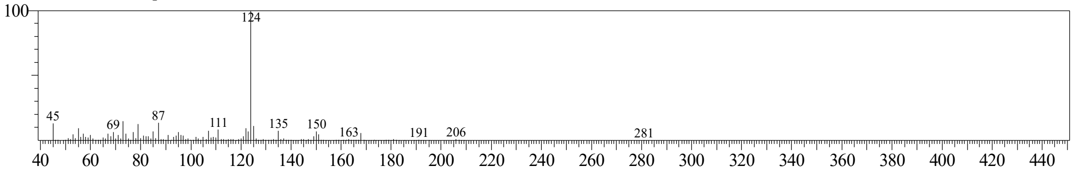


Hit#:1 Entry:31721 Library:NIST11.lib
SI:75 Formula:C12H20O CAS:54344-82-0 MolWeight:180 RetIndex:1309
CompName:5,5,8a-Trimethyl-3,5,6,7,8,8a-hexahydro-2H-chromene

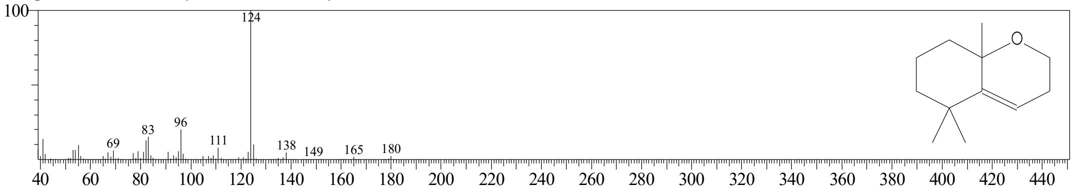


Hit#:2 Entry:30869 Library:NIST11.lib
SI:75 Formula:C12H21N CAS:0-00-0 MolWeight:179 RetIndex:1328
CompName:2,3-Bis(1-methylallyl)pyrrolidine

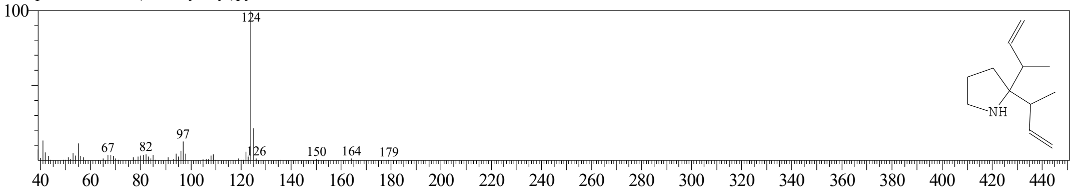


Hit#:3 Entry:47203 Library:NIST11.lib
SI:74 Formula:C14H23N CAS:20144-36-9 MolWeight:205 RetIndex:1703
CompName:Bis(cyclohex-3-enylmethyl)amine $$ 3-Cyclohexen-1-yl-N-(3-cyclohexen-1-ylmethyl)methanamine # $$

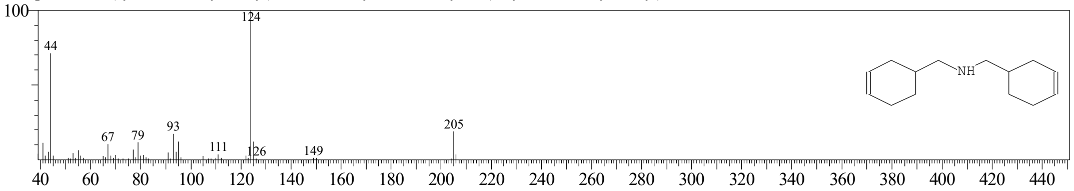


Hit#:4 Entry:32857 Library:NIST11.lib
SI:72 Formula:C11H18O2 CAS:0-00-0 MolWeight:182 RetIndex:1368
CompName:2,6,8-Trimethylbicyclo[4.2.0]oct-2-ene-1,8-diol

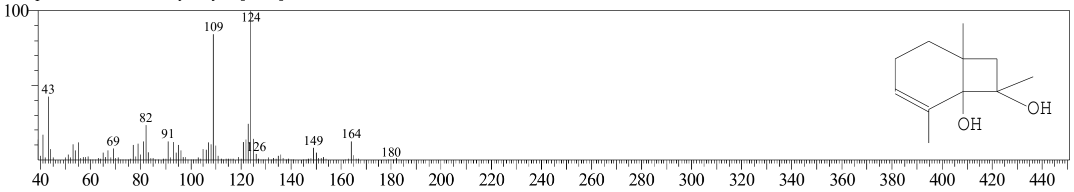


Hit#:5 Entry:24582 Library:NIST11s.lib
SI:71 Formula:C16H21NO3 CAS:87-00-3 MolWeight:275 RetIndex:2138
CompName:Homatropine $$ Benzeneacetic acid, .alpha.-hydroxy-, 8-methyl-8-azabicyclo[3.2.1]oct-3-yl ester, endo-(.+/-.)- $$ 1.alpha.H,5.alpha.H-Tropan-3-.al

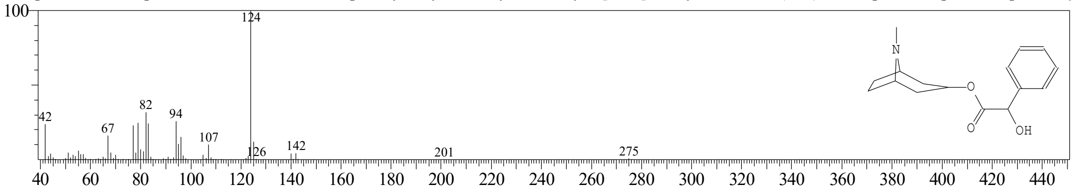

<< Target >>
Line#:15 R.Time:17.517(Scan#:1443) MassPeaks:152
RawMode:Single 17.517(1443) BasePeak:73.00(871140)
BG Mode:None Group 1 - Event 1

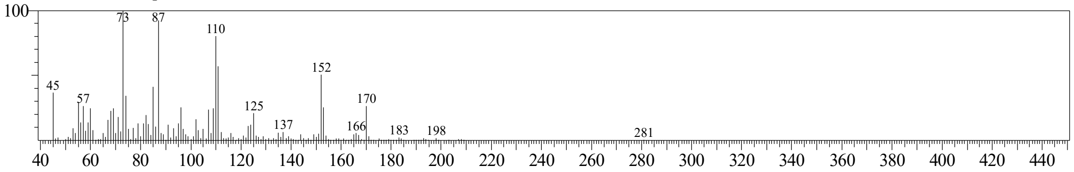


Hit#:1 Entry:135733 Library:NIST11.lib
SI:71 Formula:C21H38O2 CAS:56687-68-4 MolWeight:322 RetIndex:2203
CompName:[1,1'-Bicyclopropyl]-2-octanoic acid, 2'-hexyl-, methyl ester

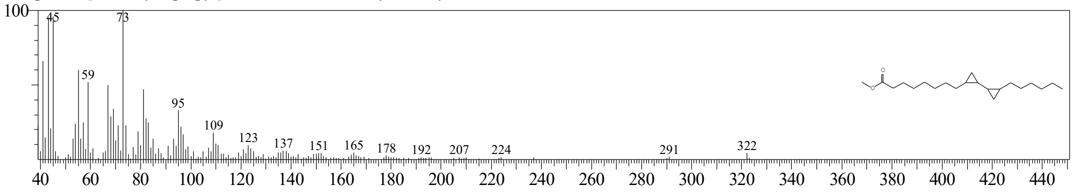


Hit#:2 Entry:33760 Library:NIST11.lib
SI:69 Formula:C10H16O3 CAS:26845-42-1 MolWeight:184 RetIndex:1417
CompName:Cyclohexanepropanoic acid, 3-oxo-, methyl ester $$ Methyl 3-(3-oxocyclohexyl)propanoate # $$

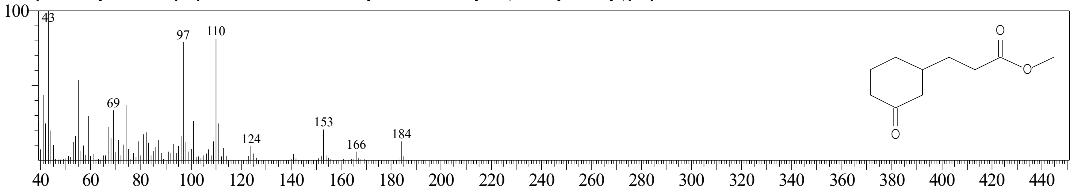


Hit#:3 Entry:33908 Library:NIST11.lib
SI:66 Formula:C11H20O2 CAS:0-00-0 MolWeight:184 RetIndex:1203
CompName:(S)-(-)-Citronellic acid, methyl ester

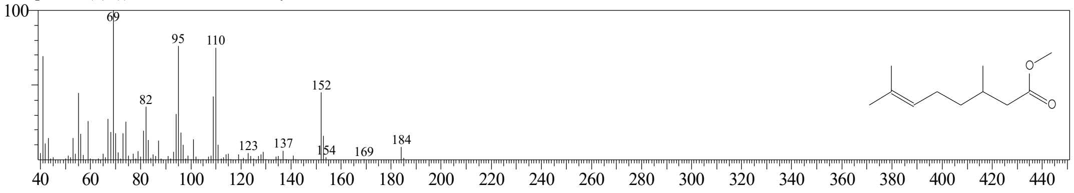


Hit#:4 Entry:24506 Library:NIST11.lib
SI:65 Formula:C10H16O2 CAS:86428-60-6 MolWeight:168 RetIndex:1407
CompName:4-Acetonylcycloheptanone $$ 4-(2-Oxopropyl)cycloheptanone # $$

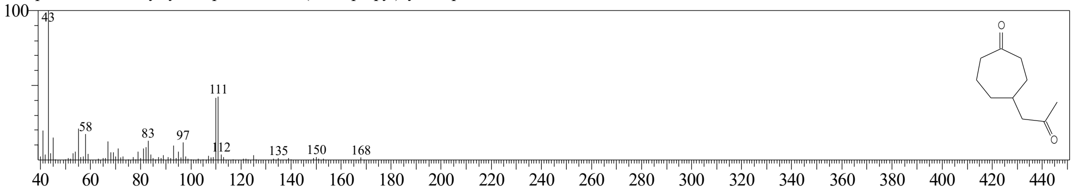


Hit#:5 Entry:62263 Library:NIST11.lib
SI:65 Formula:C14H26O2 CAS:0-00-0 MolWeight:226 RetIndex:1570
CompName:Butyl 9-decenoate

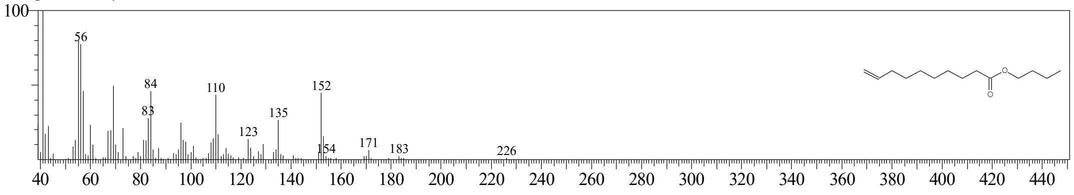

<< Target >>
Line#:16 R.Time:17.883(Scan#:1487) MassPeaks:146
RawMode:Single 17.883(1487) BasePeak:71.00(116306)
BG Mode:None Group 1 - Event 1

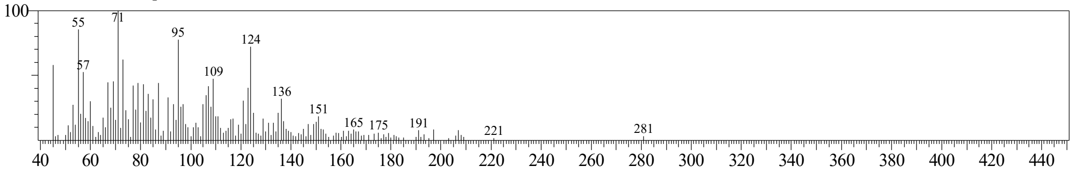


Hit#:1 Entry:135733 Library:NIST11.lib
SI:72 Formula:C21H38O2 CAS:56687-68-4 MolWeight:322 RetIndex:2203
CompName:[1,1'-Bicyclopropyl]-2-octanoic acid, 2'-hexyl-, methyl ester

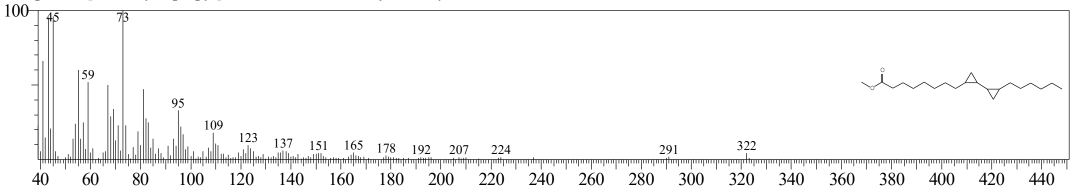


Hit#:2 Entry:126292 Library:NIST11.lib
SI:72 Formula:C18H30O4 CAS:0-00-0 MolWeight:310 RetIndex:2228
CompName:Acetic acid, 2-(2-acetoxy-2,5,5,8a-tetramethyldecalin-1-yl)- $$ [2-(Acetyloxy)-2,5,5,8a-tetramethyldecahydro-1-naphthalenyl]acetic acid # $$

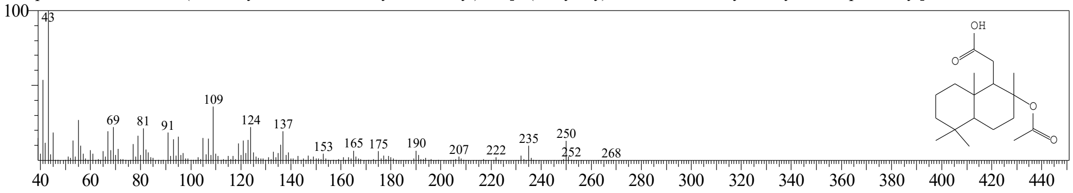


Hit#:3 Entry:49415 Library:NIST11.lib
SI:71 Formula:C13H20O2 CAS:0-00-0 MolWeight:208 RetIndex:1639
CompName:3-(1-Methylhept-1-enyl)-5-methyl-2,5-dihydrofuran-2-one $$ 5-Methyl-3-[(1E)-1-methyl-1-heptenyl]-2(5H)-furanone # $$

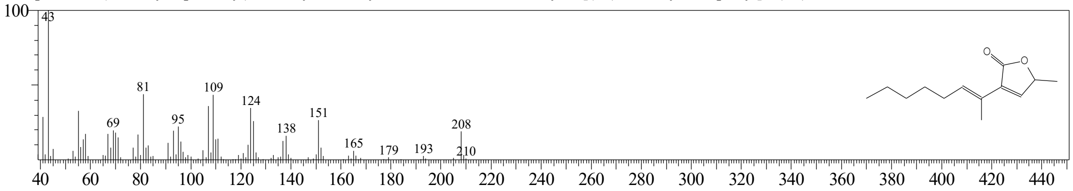


Hit#:4 Entry:57736 Library:NIST11.lib
SI:71 Formula:C15H24O CAS:0-00-0 MolWeight:220 RetIndex:1531
CompName:cis-Z-.alpha.-Bisabolene epoxide $$ 4-[(1Z)-1,5-Dimethyl-1,4-hexadienyl]-1-methyl-7-oxabicyclo[4.1.0]heptane # $$

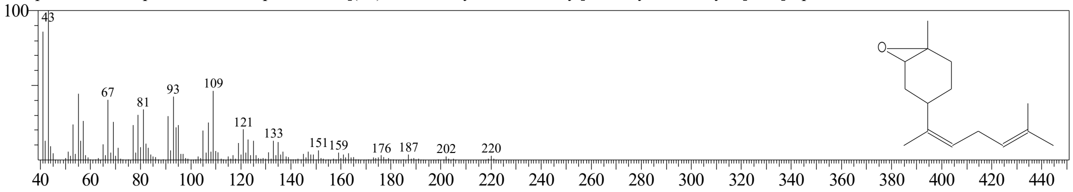


Hit#:5 Entry:69289 Library:NIST11.lib
SI:71 Formula:C15H24O2 CAS:61050-91-7 MolWeight:236 RetIndex:1626
CompName:Spiro[4.5]decan-7-one, 1,8-dimethyl-8,9-epoxy-4-isopropyl-

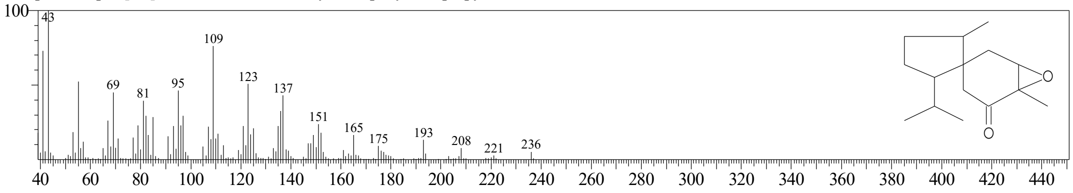

<< Target >>
Line#:17 R.Time:18.033(Scan#:1505) MassPeaks:168
RawMode:Single 18.033(1505) BasePeak:73.00(307407)
BG Mode:None Group 1 - Event 1

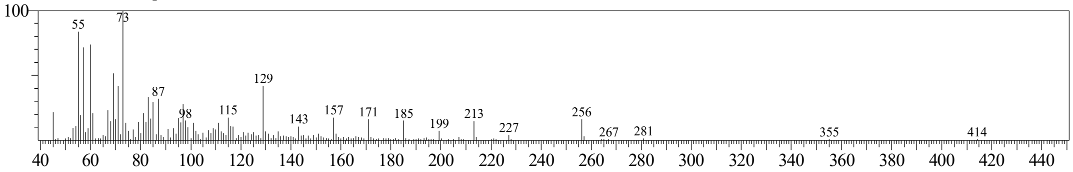


Hit#:1 Entry:23313 Library:NIST11s.lib
SI:88 Formula:C16H32O2 CAS:57-10-3 MolWeight:256 RetIndex:1968
CompName:n-Hexadecanoic acid $$ Hexadecanoic acid $$ n-Hexadecoic acid $$ Palmitic acid $$ Pentadecanecarboxylic acid $$ 1-Pentadecanecarboxylic acid

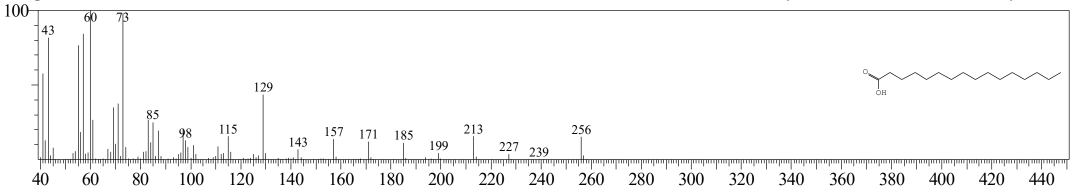


Hit#:2 Entry:128151 Library:NIST11.lib
SI:87 Formula:C20H40O2 CAS:506-30-9 MolWeight:312 RetIndex:2366
CompName:Eicosanoic acid $$ Arachic acid $$ Arachidic acid $$ Icosanoic acid $$ n-Eicosanoic acid $$ Arachidic acid (synthetic) $$

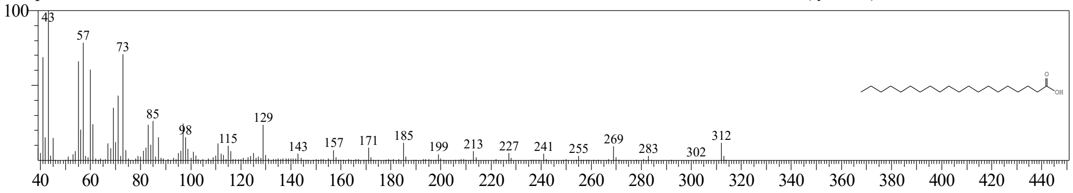


Hit#:3 Entry:211367 Library:NIST11.lib
SI:86 Formula:C38H68O8 CAS:28474-90-0 MolWeight:652 RetIndex:4765
CompName:l-(+)-Ascorbic acid 2,6-dihexadecanoate

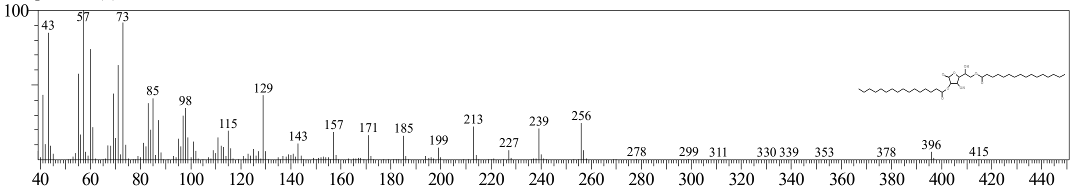


Hit#:4 Entry:73851 Library:NIST11.lib
SI:86 Formula:C15H30O2 CAS:1002-84-2 MolWeight:242 RetIndex:1869
CompName:Pentadecanoic acid $$ Pentadecylic acid $$ n-Pentadecanoic acid $$ n-Pentadecylic acid $$

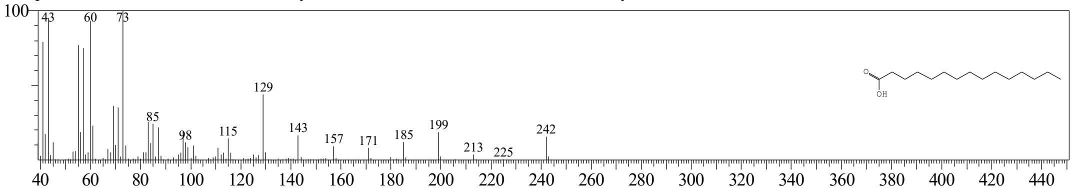


Hit#:5 Entry:23305 Library:NIST11s.lib
SI:85 Formula:C16H32O2 CAS:57-10-3 MolWeight:256 RetIndex:1968
CompName:n-Hexadecanoic acid $$ Hexadecanoic acid $$ n-Hexadecoic acid $$ Palmitic acid $$ Pentadecanecarboxylic acid $$ 1-Pentadecanecarboxylic acid

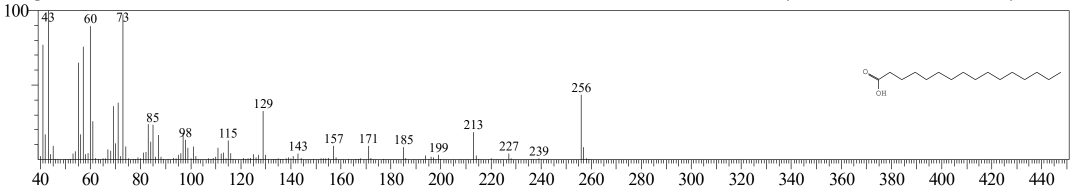

<< Target >>
Line#:18 R.Time:18.167(Scan#:1521) MassPeaks:171
RawMode:Single 18.167(1521) BasePeak:88.00(215396)
BG Mode:None Group 1 - Event 1

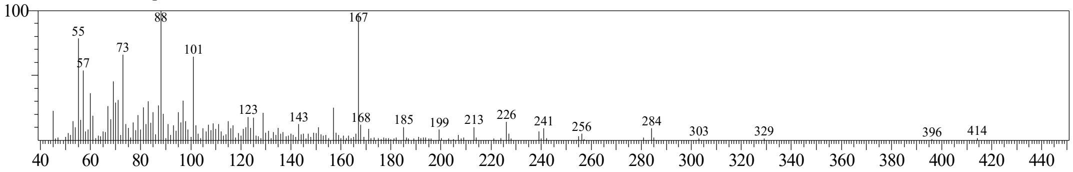


Hit#:1 Entry:117162 Library:NIST11.lib
SI:77 Formula:C19H38O2 CAS:0-00-0 MolWeight:298 RetIndex:2013
CompName:Ethyl 14-methyl-hexadecanoate

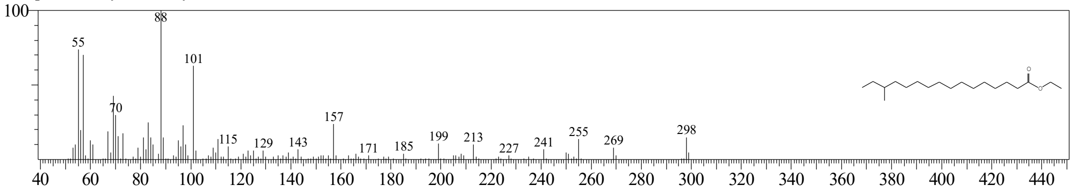


Hit#:2 Entry:52287 Library:NIST11.lib
SI:74 Formula:C13H24O2 CAS:692-86-4 MolWeight:212 RetIndex:1471
CompName:10-Undecenoic acid, ethyl ester $$ Ethyl undecenoate $$ Ethyl undecylenate $$ Ethyl 10-undecenoate $$ Undecenoic acid, ethyl ester $$ Ethyl 10-[illegible]

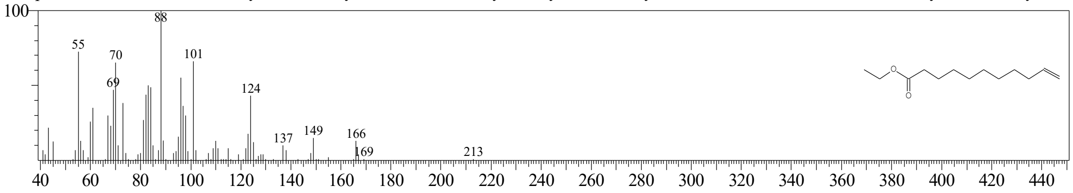


Hit#:3 Entry:25180 Library:NIST11s.lib
SI:73 Formula:C18H36O2 CAS:628-97-7 MolWeight:284 RetIndex:1978
CompName:Hexadecanoic acid, ethyl ester $$ Palmitic acid, ethyl ester $$ Ethyl hexadecanoate $$ Ethyl palmitate $$ Ethyl n-hexadecanoate $$

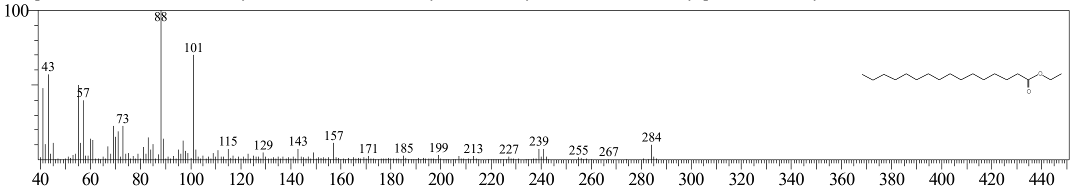


Hit#:4 Entry:43826 Library:NIST11.lib
SI:72 Formula:C11H20O3 CAS:3433-16-7 MolWeight:200 RetIndex:1470
CompName:Nonanoic acid, 9-oxo-, ethyl ester $$ Ethyl 9-oxononanoate # $$

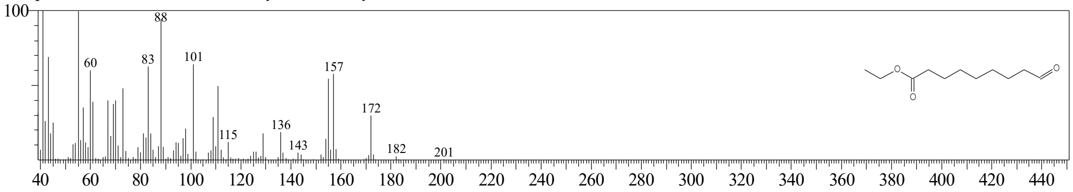


Hit#:5 Entry:166830 Library:NIST11.lib
SI:71 Formula:C24H48O2 CAS:5908-87-2 MolWeight:368 RetIndex:2574
CompName:Docosanoic acid, ethyl ester $$ Ethyl docosanoate # $$

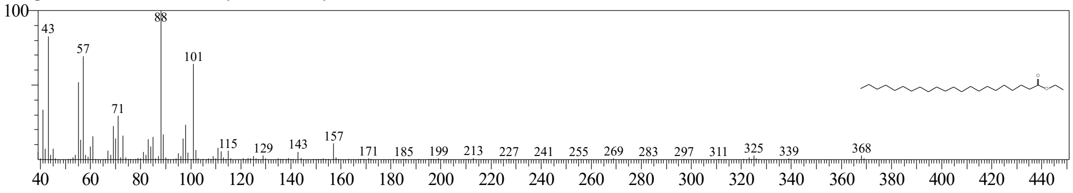

<< Target >>
Line#:19 R.Time:18.475(Scan#:1558) MassPeaks:182
RawMode:Single 18.475(1558) BasePeak:69.05(741782)
BG Mode:None Group 1 - Event 1

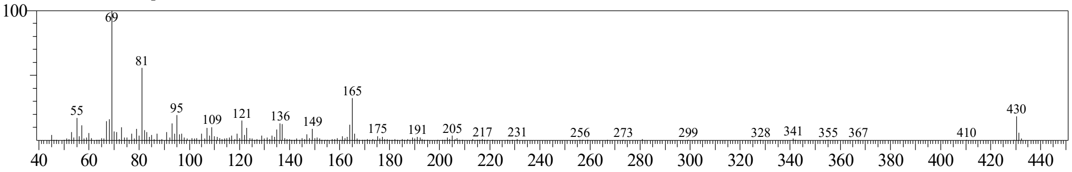


Hit#:1 Entry:30137 Library:NIST11s.lib
SI:67 Formula:C29H50O2 CAS:59-02-9 MolWeight:430 RetIndex:3149
CompName:Vitamin E $$ 2H-1-Benzopyran-6-ol, 3,4-dihydro-2,5,7,8-tetramethyl-2-(4,8,12-trimethyltridecyl)-, [2R-[2R*(4R*,8R*)]]- $$ .alpha.-Tocopherol $$

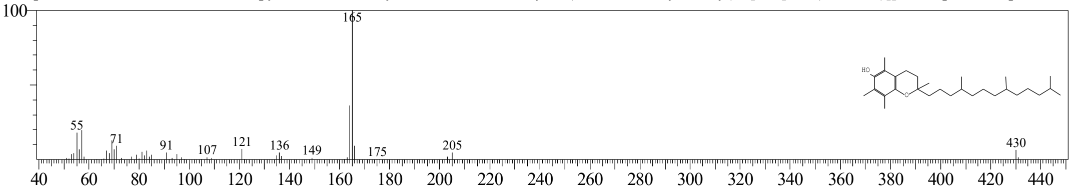


Cannot read library data

Hit#:3 Entry:30136 Library:NIST11s.lib
SI:62 Formula:C29H50O2 CAS:59-02-9 MolWeight:430 RetIndex:3149
CompName:Vitamin E $$ 2H-1-Benzopyran-6-ol, 3,4-dihydro-2,5,7,8-tetramethyl-2-(4,8,12-trimethyltridecyl)-, [2R-[2R*(4R*,8R*)]]- $$ .alpha.-Tocopherol $$

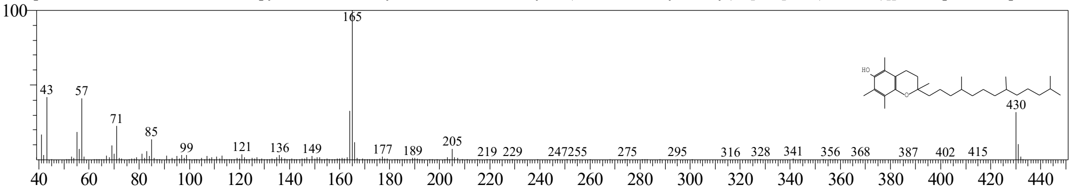


Hit#:4 Entry:192216 Library:NIST11.lib
SI:61 Formula:C29H50O2 CAS:4707-85-1 MolWeight:430 RetIndex:2721
CompName:Cholest-4-ene, 3.beta.-(methoxymethoxy)- $$ 3-(Methoxymethoxy)cholest-4-ene # $$

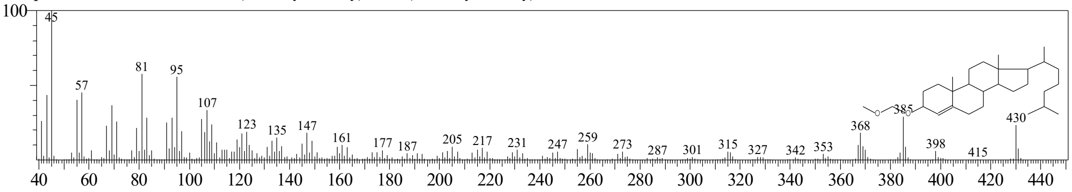


Hit#:5 Entry:192221 Library:NIST11.lib
SI:59 Formula:C29H50O2 CAS:10191-41-0 MolWeight:430 RetIndex:3149
CompName:dl-.alpha.-Tocopherol $$ (.+/-.)-.alpha.-Tocopherol $$ 2H-1-Benzopyran-6-ol, 3,4-dihydro-2,5,7,8-tetramethyl-2-(4,8,12-trimethyltridecyl)- $$ 6-Ch

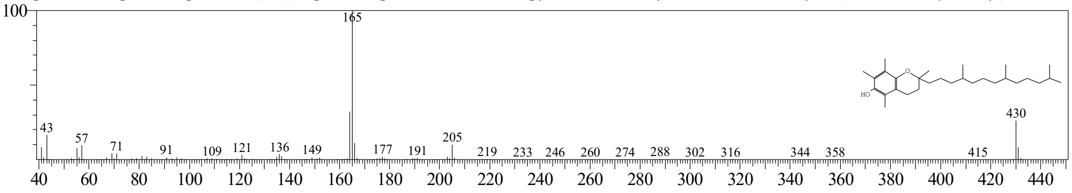

<< Target >>
Line#:20 R.Time:18.958(Scan#:1616) MassPeaks:153
RawMode:Single 18.958(1616) BasePeak:71.05(160629)
BG Mode:None Group 1 - Event 1

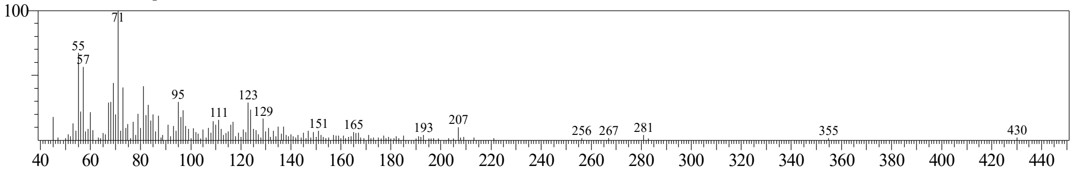


Hit#:1 Entry:25944 Library:NIST11s.lib
SI:79 Formula:C20H40O CAS:150-86-7 MolWeight:296 RetIndex:2045
CompName:Phytol $$ 2-Hexadecen-1-ol, 3,7,11,15-tetramethyl-, [R-[R*,R*-(E)]]- $$ trans-Phytol $$ 3,7,11,15-Tetramethyl-2-hexadecen-1-ol-, (2E,7R,11R)- $

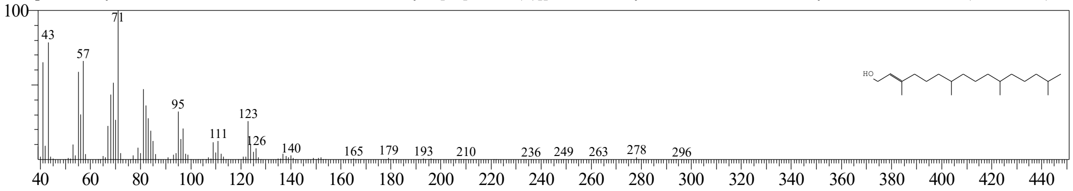


Hit#:2 Entry:135733 Library:NIST11.lib
SI:78 Formula:C21H38O2 CAS:56687-68-4 MolWeight:322 RetIndex:2203
CompName:[1,1'-Bicyclopropyl]-2-octanoic acid, 2'-hexyl-, methyl ester

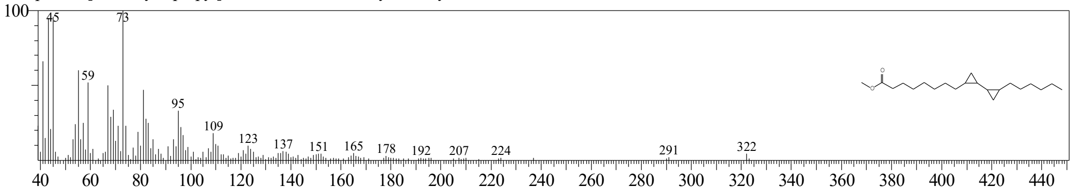


Hit#:3 Entry:25037 Library:NIST11s.lib
SI:76 Formula:C18H34O2 CAS:112-80-1 MolWeight:282 RetIndex:2175
CompName:Oleic Acid $$ 9-Octadecenoic acid (Z)- $$ .DELTA.9-cis-Oleic acid $$ cis-Oleic Acid $$ cis-9-Octadecenoic Acid $$ Emersol 211 $$ Emersol 220 \

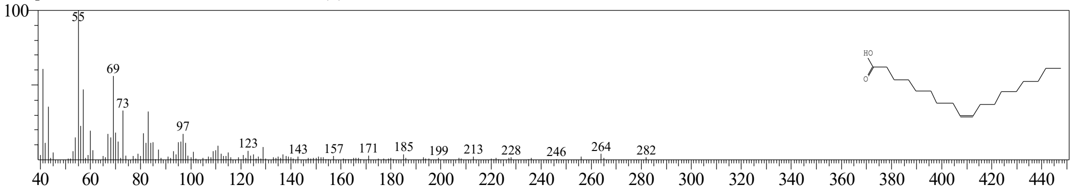


Hit#:4 Entry:115518 Library:NIST11.lib
SI:76 Formula:C20H40O CAS:102608-53-7 MolWeight:296 RetIndex:2045
CompName:3,7,11,15-Tetramethyl-2-hexadecen-1-ol $$ 2-Hexadecen-1-ol, 3,7,11,15-tetramethyl $$

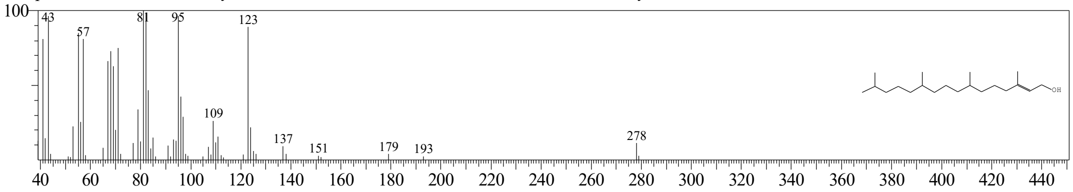


Hit#:5 Entry:102809 Library:NIST11.lib
SI:76 Formula:C18H32O2 CAS:34450-18-5 MolWeight:280 RetIndex:2165
CompName:17-Octadecynoic acid

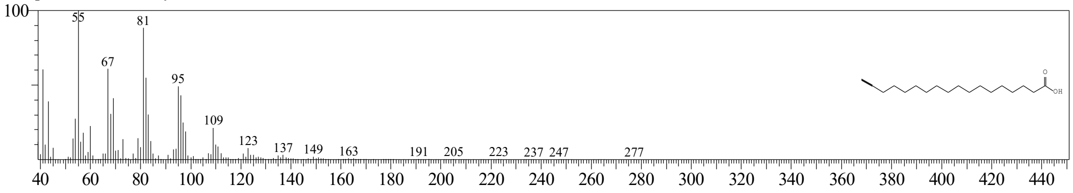

<< Target >>
Line#:21 R.Time:19.233(Scan#:1649) MassPeaks:170
RawMode:Single 19.233(1649) BasePeak:55.00(174562)
BG Mode:None Group 1 - Event 1

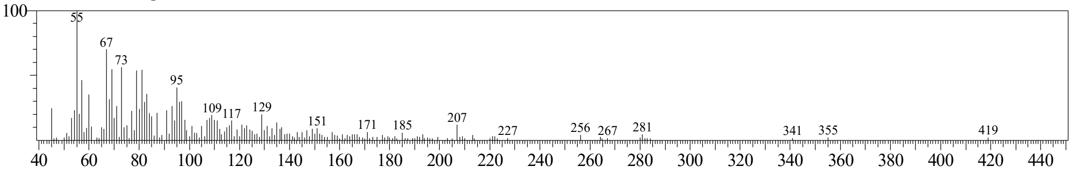


Hit#:1 Entry:24902 Library:NIST11s.lib
SI:84 Formula:C18H32O2 CAS:60-33-3 MolWeight:280 RetIndex:2183
CompName:9,12-Octadecadienoic acid (Z,Z)- $$ cis-9,cis-12-Octadecadienoic acid $$ cis,cis-Linoleic acid $$ Grape seed oil $$ Linoleic $$ Linoleic acid $$ Li

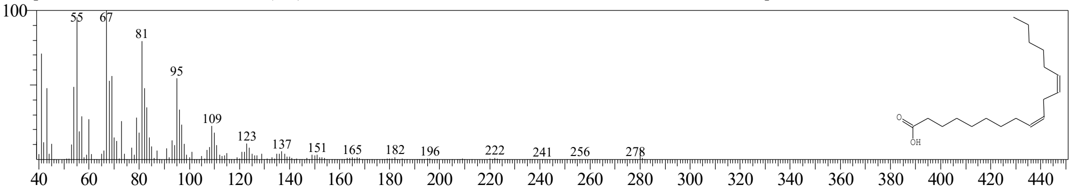


Hit#:2 Entry:144979 Library:NIST11.lib
SI:81 Formula:C22H38O2 CAS:10152-71-3 MolWeight:334 RetIndex:2266
CompName:Cyclopropaneoctanoic acid, 2-[[2-[(2-ethylcyclopropyl)methyl]cyclopropyl]methyl]-, methyl ester $$ Methyl 8-[2-((2-[(2-ethylcyclopropyl)methyl]c

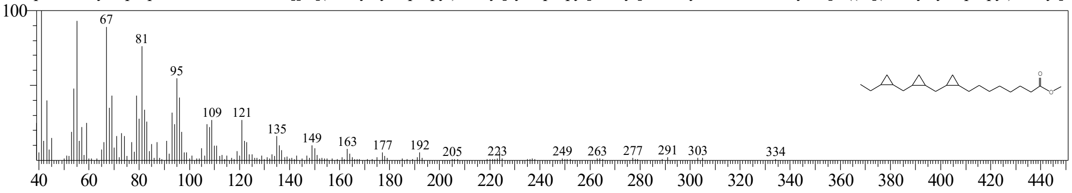


Hit#:3 Entry:102809 Library:NIST11.lib
SI:80 Formula:C18H32O2 CAS:34450-18-5 MolWeight:280 RetIndex:2165
CompName:17-Octadecynoic acid

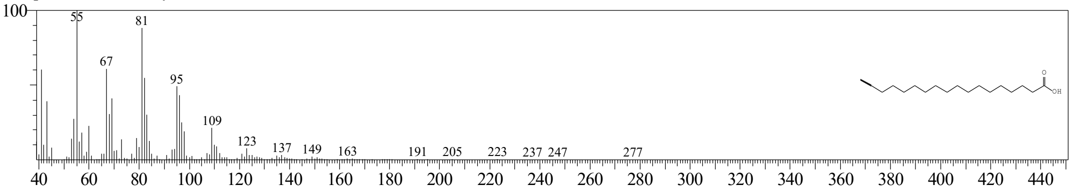


Hit#:4 Entry:26018 Library:NIST11s.lib
SI:80 Formula:C18H31ClO CAS:7459-33-8 MolWeight:298 RetIndex:2139
CompName:9,12-Octadecadienoyl chloride, (Z,Z)- $$ Linoleoyl chloride $$ Lineoleoyl chloride $$ Linoleic acid chloride $$ (9E,12E)-9,12-Octadecadienoyl ch

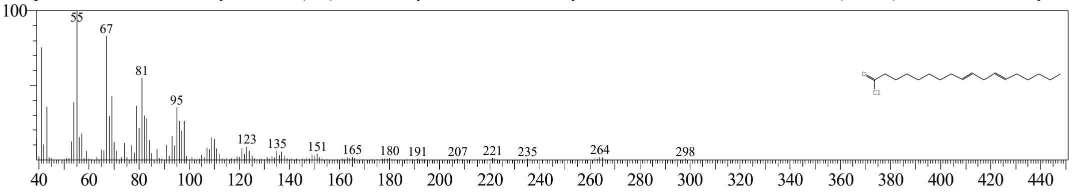


Hit#:5 Entry:91948 Library:NIST11.lib
SI:79 Formula:C18H34O CAS:0-00-0 MolWeight:266 RetIndex:2069
CompName:Z,Z-3,13-Octadecedien-1-ol $$ (4Z,13Z)-4,13-Octadecadien-1-ol # $$

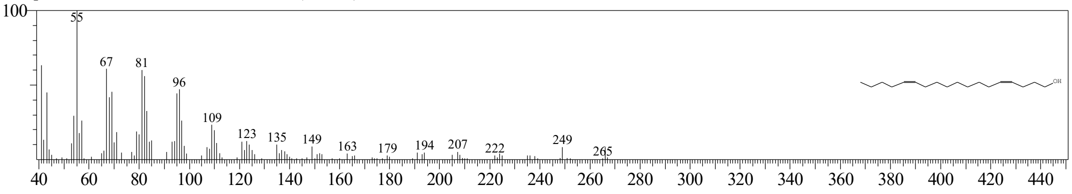

Supplement: Supplementary file 1 [file ijms-20-05913-s001.zip › ijms-580483 supplementary/ijms-580483 supplement 2.pdf]
